# Supplementary material for: Balanced networks under spike-time dependent plasticity
Source: PLoS Comput Biol. 2021 May 12;17(5):e1008958. doi: 10.1371/journal.pcbi.1008958 (PMC8143429; doi:10.1371/journal.pcbi.1008958)
Supplement: S1 Appendix — This supplementary text contains (1) a review of classical mean–field theory of firing rates and spike count covariances in balanced networks; (2) the derivation of the equation that describes mean synaptic weights, a derivation of conditions under which synaptic weights do not change signs when undergoing inhibitory STDP, and general remarks on how synaptic weights can be affected by changes in rates or covariances; and (3) supporting results on separation of timescales, synaptic weight transient dynamics, stability of weights under Kohonen’s rule, statistics and stability of synaptic weights under several STDP rules, the general impact of correlations in synaptic weights, a network undergoing iSTDP where synaptic weights change signs, and stability of iSTDP on EI and II synaptic weights. Fig A. STDP windows of different plasticity rules. a: Change in synaptic weights as a function of the relative timing of pre– and post–synaptic spikes in Classical Hebbian STDP (same as weight–dependent Hebbian). b: Same as a, but for inhibitory STDP. c: Same as a, but for Kohonen’s rule when weights are below parameter β. d: Same as c, but for the case when weights are above β. e: Same as a, but for Oja’s rule when weights are below parameter β. f: Same as e, but for the case when weights are above β. (PDF) [file pcbi.1008958.s001.pdf]

Supporting Information 1 for: Balanced Networks under Spike–Time Dependent Plasticity

Alan Eric Akil, Robert Rosenbaum, and Krešimir Josić

S1 Appendix. Review of mean–field theory in balanced networks and supporting results

Review of mean–field theory in balanced networks

We consider recurrent networks of N integrate–and–fire model neurons, N_e of which are excitatory and N_i inhibitory (with $N_\mathrm{e} = 0.8N$ and $N_\mathrm{i} = 0.2N$). This population receives feed–forward synaptic input from an external population of N_x excitatory neurons whose spike trains are modeled as Poisson processes, each having rate r_x (Unless otherwise stated, in all simulations: $r_\mathrm{x} = 10$ Hz). We use subscripts e, i and x for the local excitatory, local inhibitory, and external populations, respectively. The spike train of neuron $j = 1, \ldots, N_a$ in population $a = \mathrm{e}, \mathrm{i}, \mathrm{x}$ is represented as a sum of Dirac delta functions,

$$S_j^a(t) = \sum_n \delta(t - t_n^{a,j}) \tag{1}$$

where $t_n^{a,j}$ is the nth spike time of the neuron. The synaptic input current to neuron $j = 1, \ldots, N_a$ in population $a = \mathrm{e}, \mathrm{i}$ is given by

$$I_j^a(t) = R_j^a(t) + X_j^a(t) \tag{2}$$

where

$$R_j^a(t) = \sum_{b=\mathrm{e},\mathrm{i}} \sum_{k=1}^{N_b} J_{jk}^{ab} \sum_n \alpha_b(t - t_n^{b,k}) \tag{3}$$

is the recurrent input to the neuron from the excitatory ($b = \mathrm{e}$) and inhibitory ($b = \mathrm{i}$) neurons in the local circuit and

$$X_j^a(t) = \sum_{k=1}^{N_\mathrm{x}} J_{jk}^{a\mathrm{x}} \sum_n \alpha_\mathrm{x}(t - t_n^{\mathrm{x},k}) \tag{4}$$

is the feed–forward input from the external population. Here, J_{jk}^{ab} is the synaptic weight from presynaptic neuron k in population b to postsynaptic neuron j in population a, and $\alpha_b(t)$ is a postsynaptic current (PSC) waveform, and is defined by

$$\alpha_b(t) = \tau_b^{-1} e^{-t/\tau_b} H(t) \tag{5}$$

where $H(t)$ is the Heaviside step function, and τ_b is the synaptic timescale of neurons in population $b = \mathrm{e}, \mathrm{i}, \mathrm{x}$. In all simulations we take $\tau_\mathrm{x} = 10$ ms, $\tau_\mathrm{e} = 8$ ms, and $\tau_\mathrm{i} = 4$ ms [1]. Without loss of generality, we assume that $\int \alpha_b(t) = 1$.

The membrane potential of neuron $j = 1, \ldots, N_a$ in population $a = \mathrm{e}, \mathrm{i}$ obeys exponential integrate–and–fire dynamics [2],

$$C_m \frac{dV_j^a}{dt} = -g_L(V_j^a - E_L) + g_L \Delta_T e^{(V_j^a - V_T)/\Delta_T} + I_j^a(t). \tag{6}$$

See Table A for a description and values of the parameters of the voltage dynamics. In our simulations, this equation was integrated using Forward Euler's method with step size $dt = 0.1$ ms. We consider a random, blockwise–Erdős–Rényi connectivity structure with

$$J_{jk}^{ab} = \frac{1}{\sqrt{N}} \begin{cases} j_{ab} & \text{with prob. } p_{ab} \\ 0 & \text{otherwise,} \end{cases} \tag{7}$$

where connections are statistically independent and $j_{ab}, p_{ab} \sim \mathcal{O}(1)$ for $b = \mathrm{e}, \mathrm{i}, \mathrm{x}$ and $a = \mathrm{e}, \mathrm{i}$.

Mean field theory of firing rates in balanced networks

We denote by r_a the mean firing rate of neurons in population $a = \mathrm{e}, \mathrm{i}, \mathrm{x}$, and let the components of the 2×1 vector,

$$\boldsymbol{r} = \begin{bmatrix} r_\mathrm{e} \\ r_\mathrm{i} \end{bmatrix}, \tag{8}$$

describe the mean activity of both subpopulations. The mean–field synaptic inputs to neurons in populations $a = \mathrm{e}, \mathrm{i}$ are denoted by $\overline{U}_a = \langle U_j^a(t) \rangle_j$ for $a = \mathrm{e}, \mathrm{i}$ and $U = I, X, R$. In vector form,

$$\overline{\boldsymbol{U}} = \begin{bmatrix} \overline{U}_\mathrm{e} \\ \overline{U}_\mathrm{i} \end{bmatrix}. \tag{9}$$

We also define the recurrent and feed–forward mean–field connectivity matrices,

$$\overline{W} = \begin{bmatrix} \overline{w}_\mathrm{ee} & \overline{w}_\mathrm{ei} \\ \overline{w}_\mathrm{ie} & \overline{w}_\mathrm{ii} \end{bmatrix}, \quad \text{and} \quad \overline{W}_x = \begin{bmatrix} \overline{w}_\mathrm{ex} \\ \overline{w}_\mathrm{ix} \end{bmatrix}, \tag{10}$$

where $\overline{w}_{ab} = p_{ab} j_{ab} q_b \sim \mathcal{O}(1)$. Here, we have defined $q_b = N_b/N$ which are assumed to be $\mathcal{O}(1)$.

We review the mean–field analysis of firing rates in the balanced state [3]. The mean external input to each population is given by

$$\overline{\boldsymbol{X}} = \sqrt{N} \overline{W}_\mathrm{x} r_\mathrm{x}, \tag{11}$$

and the mean recurrent input by

$$\overline{\boldsymbol{R}} = \sqrt{N} \overline{W} \boldsymbol{r}. \tag{12}$$

The mean total synaptic input is therefore given by

$$\overline{\boldsymbol{I}} = \sqrt{N} \left[\overline{W} \boldsymbol{r} + \overline{W}_\mathrm{x} r_\mathrm{x} \right]. \tag{13}$$

In the balanced state, we have $\overline{\boldsymbol{I}}, \boldsymbol{r} \sim \mathcal{O}(1)$, which can only be obtained by a cancellation between external and recurrent synaptic inputs. This cancellation requires $\overline{W} \boldsymbol{r} + \overline{W}_\mathrm{x} r_\mathrm{x} \sim \mathcal{O}(1/\sqrt{N})$ so that

$$\lim_{N \to \infty} \boldsymbol{r} = -\overline{W}^{-1} \overline{W}_\mathrm{x} r_\mathrm{x} \tag{14}$$

in the balanced state, provided $\overline{X}_e / \overline{X}_i > \overline{w}_{ei} / \overline{w}_{ii} > \overline{w}_{ee} / \overline{w}_{ie}$ [3, 4]. The firing rates in Eq. (14) depend only on the recurrent structure and mean input, and are hence independent of the correlation structure in the network.

Generating correlated spike trains

In our simulations we generated spike trains, S_j^{x}, for the external population following [1, 5, 6]. Here, we review the Multiple Interaction Process (MIP) algorithm for generating correlated Poisson processes. To generate N_{x} processes, all having firing rate r_{x} and pairwise correlation c_{x}, we first generate a "mother" process with rate $r_m = r_{\mathrm{x}}/c_{\mathrm{x}}$. Then, to generate each of the N_{x} "daughter" processes, we first take all the spikes in the mother process and then delete each spike independently with probability $1 - c_{\mathrm{x}}$. In other words, each spike time from the mother process is included in each daughter process independently with probability c_{x}. The rates of the daughter processes are $r_m c_{\mathrm{x}} = r_{\mathrm{x}}$, as desired. Also, two daughter processes share a proportion c_{x} of spike times (since the probability that a spike time in one process also appears in the other process is c_{x}). This algorithm produces perfectly synchronous spikes. To make the spikes less synchronous, but still correlated, we "jitter" all of the daughter spike times by adding i.i.d. random variables to each daughter spike times [7].

The cross spectral density (CSD) between two stationary processes is the Fourier transform of their cross–covariance function:

$$\langle X, Y\rangle(f) := \int_{-\infty}^{\infty} c_{XY}(\tau) e^{-2\pi i f \tau} d\tau \tag{15}$$

where

$$c_{XY}(\tau) := \mathrm{cov}(X(t), Y(t+\tau)). \tag{16}$$

We also define the mean–field spectra,

$$\langle S_a, S_b\rangle = \mathrm{avg}_{j,k}\langle S_j^a, S_k^b\rangle \tag{17}$$

to be the average CSD between neurons in populations $a, b = \mathrm{e}, \mathrm{i}, \mathrm{x}$ in the network.

If the random variable (which is added to jitter spike times) has density $G(t)$ then the CSD is given by

$$\langle S_j^{\mathrm{x}}, S_k^{\mathrm{x}}\rangle(f) = \begin{cases} c_{\mathrm{x}} r_{\mathrm{x}} |\widetilde{G}(f)|^2 & j \neq k \\ r_{\mathrm{x}} & j = k \end{cases} \tag{18}$$

where $\widetilde{G}(f)$ is the Fourier transform of $G(t)$.

In our simulations, we use Gaussian–distributed jitters with standard deviation $\tau_{\mathrm{jitter}} = 5$ ms, and we get

$$\langle S_j^{\mathrm{x}}, S_k^{\mathrm{x}}\rangle = c_{\mathrm{x}} r_{\mathrm{x}} e^{-4f^2\pi^2\tau_{\mathrm{jitter}}^2} \tag{19}$$

if $j \neq k$.

Mean–field theory of covariances in non–plastic balanced networks

Here we just state results already derived in [1]. We define the mean–field spike count covariance matrix as,

$$C = \begin{bmatrix} C_{\mathrm{ee}} & C_{\mathrm{ei}} \\ C_{\mathrm{ie}} & C_{\mathrm{ii}} \end{bmatrix} \tag{20}$$

where C_{ab} is the mean spike count covariance between neurons in populations $a = \mathrm{e}, \mathrm{i}$ and $b = \mathrm{e}, \mathrm{i}$ respectively. In the balanced state for large enough N, this is given by

$$C \approx \frac{1}{N} T_{\mathrm{win}} \overline{W}^{-1} \Gamma \overline{W}^{-T} \tag{21}$$

where T_{win} is the size of the counting window used. The matrix Γ has the same structure as C and represents the covariance between external inputs.

If spike trains in the external layer are uncorrelated Poisson processes ($c_{\text{x}} = 0$) then

$$\Gamma = \overline{W}_{\text{x}} \overline{W}_{\text{x}}^T \frac{r_{\text{x}}}{q_{\text{x}}}. \tag{22}$$

so that $C \sim \mathcal{O}(1/N)$, and the network operates in the asynchronous state [3, 8]. If spike trains in the external network are correlated Poisson processes with pairwise correlation coefficient $c_{\text{x}} \neq 0$, then

$$\Gamma = N \overline{W}_{\text{x}} \overline{W}_{\text{x}}^T c_{\text{x}} r_{\text{x}} \tag{23}$$

so that $C \sim \mathcal{O}(1)$, and the network operates in a correlated state [1].

These equations describe the expected spike count covariances within and between populations in the network.

To simplify notation, we define the 2×2 matrix

$$\langle \boldsymbol{S}, \boldsymbol{S} \rangle = \begin{bmatrix} \langle S_{\text{e}}, S_{\text{e}} \rangle & \langle S_{\text{e}}, S_{\text{i}} \rangle \\ \langle S_{\text{i}}, S_{\text{e}} \rangle & \langle S_{\text{i}}, S_{\text{i}} \rangle \end{bmatrix}. \tag{24}$$

The generalization of the equations for spike count covariances to CSDs is

$$\langle \boldsymbol{S}, \boldsymbol{S} \rangle = W^{-1} W_{\text{x}} \langle S_{\text{x}}, S_{\text{x}} \rangle W_{\text{x}}^* W^{-*} \tag{25}$$

where W_{x}^* is the conjugate transpose, and W^{-*} is the inverse of the conjugate transpose,

$$W = \begin{bmatrix} w_{\text{ee}} & w_{\text{ei}} \\ w_{\text{ie}} & w_{\text{ii}} \end{bmatrix} \text{ and } W_x = \begin{bmatrix} w_{\text{ex}} \\ w_{\text{ix}} \end{bmatrix}, \tag{26}$$

$$w_{ab}(f) = p_{ab} j_{ab} q_b \widetilde{\alpha}_b(f) \sim \mathcal{O}(1) \tag{27}$$

and

$$\widetilde{\alpha}_b(f) = \frac{1}{1 + 2\pi i f \tau_b} \tag{28}$$

is the Fourier transform of $\alpha_b(t)$. Note that $W(f)$ and $W_{\text{x}}(f)$ depend on frequency. Recall from the previous section that spike trains in the external population are correlated Poisson processes generated with Gaussian–distributed jittering, hence

$$\langle S_{\text{x}}, S_{\text{x}} \rangle = c r_{\text{x}} e^{-4 f^2 \pi^2 \tau_{\text{j}itter}^2}. \tag{29}$$

To understand the relationship between these results and the spike count covariance results above, note that for stationary processes, the covariance between integrals over large time windows is related to the zero–frequency CSD according to

$$\lim_{T \to \infty} \frac{1}{T} \text{cov} \left(\int_0^T X(t) dt, \int_0^T Y(t) dt \right) = \langle X, Y \rangle(0) \tag{30}$$

so that for large T,

$$\text{cov} \left(\int_0^T X(t) dt, \int_0^T Y(t) dt \right) \approx T \langle X, Y \rangle(0). \tag{31}$$

Now note that a spike count over a window of size T is just an integral of spike trains over $[0, T]$ where $T = T_{\text{win}}$. We can write the zero–lag covariance between two stationary processes in terms of the CSD as

$$\mathrm{cov}(X(t),Y(t)) = \int_{-\infty}^{\infty} \langle X,Y\rangle(f)df. \tag{32}$$

This will be useful below in "Accounting for the effects of correlations in the general STDP rule" when deriving an expression for the evolution of mean synaptic weights. More generally, the cross–covariance function, $c(\tau) = \mathrm{cov}(X(t), Y(t+\tau))$, is the inverse Fourier transform of $\langle X,Y\rangle(f)$.

Some other useful properties of the cross–spectral operator are:

$$\langle aX + bZ, Y\rangle = a\langle X,Y\rangle + b\langle Z,Y\rangle \tag{33}$$

for $a, b \in \mathbb{C}$

$$\langle X,Y\rangle = \langle Y,X\rangle^* \tag{34}$$

where z^* is the complex conjugate of z,

$$\langle K * X, Y\rangle = \widetilde{K}\langle X,Y\rangle \tag{35}$$

where $*$ denotes convolution, $K(t)$ is an L^2 kernel, and $\widetilde{K}(f)$ is the Fourier transform. The power spectral density of X is given by $\langle X,X\rangle \in \mathbb{R}$.

It is often useful to define the cross–spectral matrix between two multivariate processes. Specifically, suppose $\vec{X}(t) \in \mathbb{R}^m$ and $\vec{Y}(t) \in R^n$ are multivariate, stationary processes then $\langle \vec{X}, \vec{Y}\rangle \in \mathbb{R}^{m\times n}$ with

$$[\langle \vec{X}, \vec{Y}\rangle]_{jk} = \langle \vec{X}_j, \vec{Y}_k\rangle. \tag{36}$$

This operator has essentially all the same properties elucidated above except that the complex conjugate turns into a conjugate–transpose.

Accounting for the effects of correlations in the general STDP rule

We now provide a detailed derivation of the mean synaptic weights accounting for the effects of firing rates and spike count covariances. We also explain how STDP rules were implemented, and show that any second order STDP rule can either shift the fixed point of the mean synaptic weights or modulate the speed of convergence to that equilibrium.

Recall that the eligibility trace, $x_j^a(t)$, of neuron j in population a evolves according to

$$\tau_{\mathrm{STDP}} \frac{dx_j^a(t)}{dt} = -x_j^a(t) + \tau_{\mathrm{STDP}} S_j^a(t), \tag{37}$$

for $a = \mathrm{e}, \mathrm{i}$, where $S_j^a(t) = \sum_n \delta(t - t_n^{a,j})$ is the sequence of spikes of neuron, j. The time constant, τ_{STDP}, defines the time over which concurrent spikes in two cells can lead to a change in synaptic weights.

We assume that all synapses can be subject to activity–dependent modulations. The synaptic weight from neuron $k = 1, \ldots, N_b$ in population $b = \mathrm{e}, \mathrm{i}$ to neuron $j = 1, \ldots, N_a$ in population $a = \mathrm{e}, \mathrm{i}$ changes according to a generalized spike–timing dependent plasticity (STDP) rule,

$$\frac{dJ_{jk}^{ab}}{dt} = \eta_{ab}\left(A_0 + \sum_{\alpha=\{a,j\},\{b,k\}} A_\alpha S_\alpha + \sum_{\alpha,\beta=\{a,j\},\{b,k\}} B_{\alpha,\beta} x_\alpha S_\beta\right) \tag{38}$$

where η_{ab} is the learning rate that defines the timescale of synaptic weight changes, $A_0, A_\alpha, B_{\alpha\beta}$ are functions of the synaptic weight J_{jk}^{ab} (since the change in the synaptic weight can depend on the current value of the synaptic weight in a linear or nonlinear manner), and $a, b = \mathrm{e}, \mathrm{i}$. For instance, the term $B_{(e,k),(i,j)} x_k^{\mathrm{e}} S_j^{\mathrm{i}}$ describes the contribution due to a spike in post–synaptic cell j in the inhibitory subpopulation, at the given value of the eligibility trace in the pre–synaptic cell k in the excitatory subpopulation.

We assumed that changes in synaptic weights occur on longer timescales than the dynamics of the eligibility trace and the correlation timescale, *i.e.* $1/\eta_{ab} \gg T_{\mathrm{win}}, \tau_{\mathrm{STDP}}$ [9–14]. Let ΔT be a time larger than the timescale of eligibility traces, τ_{STDP}, and T_{win}, but smaller than $1/\eta_{ab}$, so that the time differential of the weights satisfies [14]:

$$\frac{\Delta J_{jk}^{ab}}{\Delta T} = \frac{\eta_{ab}}{\Delta T} \int_0^{\Delta T} \Big[A_0 + \sum_{\alpha=\{a,j\},\{b,k\}} A_\alpha S_\alpha + \sum_{\alpha,\beta=\{a,j\},\{b,k\}} B_{\alpha,\beta} x_\alpha S_\beta \Big] dt. \tag{39}$$

We expand the sums and take split the integral over the terms in the sums,

$$\begin{aligned} \frac{\Delta J_{jk}^{ab}}{\Delta T} = \frac{\eta_{ab}}{\Delta T} \bigg(& \Delta T A_0 + A_{a,j} \int_0^{\Delta T} S_j^a dt + A_{b,k} \int_0^{\Delta T} S_k^b dt \\ & + B_{\{a,j\},\{a,j\}} \int_0^{\Delta T} x_j^a S_j^a dt + B_{\{a,j\},\{b,k\}} \int_0^{\Delta T} x_j^a S_k^b dt \\ & + B_{\{b,k\},\{a,j\}} \int_0^{\Delta T} x_k^b S_j^a dt + B_{\{b,k\},\{b,k\}} \int_0^{\Delta T} x_k^b S_k^b dt \bigg). \end{aligned} \tag{40}$$

Consider each integral that is multiplied by $1/\Delta T$ as a sample of joint statistics. Then:

$$\begin{aligned} \frac{\Delta J_{jk}^{ab}}{\Delta T} = \eta_{ab} \bigg(& A_0 + A_{a,j} \mathrm{E}[S_j^a] + A_{b,k} \mathrm{E}[S_k^b] + B_{\{a,j\},\{a,j\}} \mathrm{E}[x_j^a S_j^a] \\ & + B_{\{a,j\},\{b,k\}} \mathrm{E}[x_j^a S_k^b] + B_{\{b,k\},\{a,j\}} \mathrm{E}[x_k^b S_j^a] + B_{\{b,k\},\{b,k\}} \mathrm{E}[x_k^b S_k^b] \bigg) \end{aligned} \tag{41}$$

where $\mathrm{E}[\cdot]$ denotes expectation over time.

We provide a detailed derivation for the term $B_{\{a,j\},\{b,k\}} \mathrm{E}[x_j^a S_k^b]$. The other terms are derived in the same way. First note that

$$\mathrm{E}[x_j^a S_k^b] = \mathrm{E}[(K * S_j^a)(t)(S_k^b(t)] = \mathrm{cov}(K * S_j^a, S_k^b) + \mathrm{E}[K * S_j^a] \mathrm{E}[S_k^b] \tag{42}$$

where $K(t) = e^{-t/\tau_{\mathrm{STDP}}} H(t)$ and $H(t)$ is the Heaviside function. The second term on the right hand side can be written in terms of the rates,

$$\mathrm{E}[K * S_j^a] \mathrm{E}[S_k^b] = \tau_{STDP} r_j^a r_k^b \tag{43}$$

where r_j^a is the rate of neuron j in population a and we used the fact that $\int_{-\infty}^{\infty} K(t) dt = \tau_{STDP}$. We showed earlier in "Mean–field theory of covariances in non–plastic balanced networks" that,

$$\mathrm{cov}(K * S_j^a, S_k^b) = \int_{-\infty}^{\infty} \langle K * S_j^a, S_k^b \rangle (f) df. \tag{44}$$

This can be simplified by recalling that convolutions interact nicely with CSDs

$$\langle K * S_j^a, S_k^b \rangle = \widetilde{K} \langle S_j^a, S_k^b \rangle \tag{45}$$

where $\widetilde{K}(f)$ is the Fourier transform of the exponential kernel $K(t)$. This gives

$$\mathrm{cov}(K * S_j^a, S_k^b) = \int_{-\infty}^{\infty} \widetilde{K}(f) \langle S_j^a, S_k^b \rangle (f) df. \tag{46}$$

Therefore,

$$\mathrm{E}[x_j^a S_k^b] = \int_{-\infty}^{\infty} \widetilde{K}(f) \langle S_j^a, S_k^b \rangle (f) df + \tau_{STDP} r_j^a r_k^b. \tag{47}$$

The first term in the right hand side depends on the spike count covariance between spike trains of populations a and b. The second term depends on firing rates of populations a, b. Following the procedure demonstrated here and applying it to each term in Eq. (41), we arrived to the following equation describing the evolution of weights,

$$\begin{aligned}
\frac{\Delta J_{jk}^{ab}}{\Delta T} = \eta_{ab} \Bigg(& A_0 + A_{a,j} r_j^a + A_{b,k} r_k^b \\
& + B_{\{a,j\},\{a,j\}} \left(\int_{-\infty}^{\infty} \widetilde{K}(f) \langle S_j^a, S_j^a \rangle (f) df + \tau_{STDP} r_j^a r_j^a \right) \\
& + B_{\{a,j\},\{b,k\}} \left(\int_{-\infty}^{\infty} \widetilde{K}(f) \langle S_j^a, S_k^b \rangle (f) df + \tau_{STDP} r_j^a r_k^b \right) \\
& + B_{\{b,k\},\{a,j\}} \left(\int_{-\infty}^{\infty} \widetilde{K}(f) \langle S_k^b, S_j^a \rangle (f) df + \tau_{STDP} r_k^b r_j^a \right) \\
& + B_{\{b,k\},\{b,k\}} \left(\int_{-\infty}^{\infty} \widetilde{K}(f) \langle S_k^b, S_k^b \rangle (f) df + \tau_{STDP} r_k^b r_k^b \right) \Bigg).
\end{aligned} \tag{48}$$

Averaging both sides of the equation over all neurons j and k in populations a and b, respectively, we obtained:

$$\begin{aligned}
\frac{\Delta J_{ab}}{\Delta T} = \eta_{ab} \Bigg(& A_0 + A_{a,j} r_a + A_{b,k} r_b \\
& + B_{\{a,j\},\{a,j\}} \left(\int_{-\infty}^{\infty} \widetilde{K}(f) \langle S_a, S_a \rangle (f) df + \tau_{STDP} r_a r_a \right) \\
& + B_{\{a,j\},\{b,k\}} \left(\int_{-\infty}^{\infty} \widetilde{K}(f) \langle S_a, S_b \rangle (f) df + \tau_{STDP} r_a r_b \right) \\
& + B_{\{b,k\},\{a,j\}} \left(\int_{-\infty}^{\infty} \widetilde{K}(f) \langle S_b, S_a \rangle (f) df + \tau_{STDP} r_b r_a \right) \\
& + B_{\{b,k\},\{b,k\}} \left(\int_{-\infty}^{\infty} \widetilde{K}(f) \langle S_b, S_b \rangle (f) df + \tau_{STDP} r_b r_b \right) \Bigg).
\end{aligned} \tag{49}$$

Lastly, we rearranged terms and arrived at a compressed expression dependent on rates and spike count covariances:

$$\frac{dJ_{ab}}{dt} = \eta_{ab} \left(A_0 + \sum_{\alpha,\beta=\{a,b\}} \mathrm{Rate}_{\alpha,\beta} + \mathrm{Cov}_{\alpha,\beta} \right), \tag{50}$$

where

$$\begin{aligned} \mathrm{Rate}_{\alpha,\beta} &= A_\alpha r_\alpha/2 + B_{\alpha,\beta}\tau_{STDP} r_\alpha r_\beta \\ \mathrm{Cov}_{\alpha,\beta} &= B_{\alpha,\beta}\int_{-\infty}^{\infty} \widetilde{K}(f)\langle S_\alpha, S_\beta\rangle(f)df, \end{aligned} \tag{51}$$

where all the coefficients remained the same, but changed notation, e.g. $B_{\{a,j\},\{b,k\}} = B_{a,b}$.

Implementation of inhibitory plasticity in numerical simulations

We describe how we implemented the plasticity rules using the example of homeostatic inhibitory–to–excitatory STDP as in [15], which is a special case of our general STDP rule. Note that for the implementation of this rule, we will use the convention that $J^{\mathrm{ei}}_{jk} < 0$ for inhibitory weights. After each presynaptic spike, $t^{\mathrm{i}}_{k,n}$, of inhibitory neuron k, we make the update:

$$J^{\mathrm{ei}}_{jk}(t+\Delta t) = J^{\mathrm{ei}}_{jk}(t) - \eta_{\mathrm{ei}}\frac{J^{\mathrm{ei}}_{jk}(t)}{J_{\mathrm{norm}}}(x^{\mathrm{e}}_j(t) - \alpha_{\mathrm{e}}) \tag{52}$$

and after each postsynaptic spike, $t^{\mathrm{e}}_{j,m}$, of excitatory neuron j, we make the update:

$$J^{\mathrm{ei}}_{jk}(t+\Delta t) = J^{\mathrm{ei}}_{jk}(t) - \eta_{\mathrm{ei}}\frac{J^{\mathrm{ei}}_{jk}(t)}{J_{\mathrm{norm}}}x^{\mathrm{i}}_k(t) \tag{53}$$

where $J_{\mathrm{norm}} \sim \mathcal{O}(1/\sqrt{N})$ is a normalization constant. Note that $J_{\mathrm{norm}} < 0$, in order to yield a positive fraction in the previous two equations. Other STDP rules were implemented similarly.

The rule from [15] was slightly modified to prevent I to E weights from becoming positive. In particular, the right hand side of the updates was multiplied by $\frac{J^{\mathrm{ei}}_{jk}(t)}{J_{\mathrm{norm}}}$, in order to create an unstable zero fixed point and enforce that weights will remain negative. See "Supporting results and discussion" below for an example where inhibitory weights change signs if the zero fixed point is not present.

This modification guarantees that EI weights will remain negative in continuous–time rate–dynamics. However, EI weights could still change sign, since in network simulations, changes in synaptic weights occur in spike–based discrete–time updates. We thus proceed to derive a condition under which this modification ensures that synaptic weights will not change signs. Consider updates due to post–synaptic spikes, since pre–synaptic spikes always strengthen the connection. We would like for the weights to always be negative:

$$J^{ei}_{jk} = J^{ei}_{jk} - \frac{J^{ei}_{jk}}{J_{\mathrm{norm}}}\eta_{\mathrm{ei}}(x^e_j - \alpha_{\mathrm{e}}) < 0. \tag{54}$$

Dividing through by J^{ei}_{jk} and rearranging,

$$-\frac{1}{J_{\mathrm{norm}}}\eta_{\mathrm{ei}}x^e_j + \alpha_{\mathrm{e}}\eta_{\mathrm{ei}}\frac{1}{J_{\mathrm{norm}}} > -1. \tag{55}$$

Since $J_{\mathrm{norm}} < 0$, it suffices to have:

$$\alpha_{\mathrm{e}}\eta_{\mathrm{ei}}\frac{1}{|J_{\mathrm{norm}}|} < 1 \tag{56}$$

Therefore, as long as this condition, which depends on network and plasticity parameters, is satisfied, I to E weights will not turn positive.

Remarks on the general STDP rule

We would like to know what the impact of rates and covariances is in the synaptic weights. We estimate the magnitude of the two terms in the section below "General impact of correlations in synaptic weights". The remaining question is: given the magnitude of each term, what is the qualitative effect that increasing rates or covariances will have on synaptic weights? Here, we show that changes in rates or covariances has two distinct effects: they can shift the location of the fixed point, or they can modulate the speed of convergence to that equilibrium.

Consider a general STDP rule that involves synapses between neurons of different populations. In this case, both the rates and covariances determine the location of the fixed point of the synaptic weights. In particular, increasing firing rates or spike count covariances can shift the location of the fixed point of the mean synaptic weight.

For simplicity, assume the STDP only involves second order terms (these give rise to covariance terms in the mean–field equation of the weights),

$$\frac{dJ_{jk}^{ab}}{dt} = \eta_{ab}\Big(B_{\{a,j\},\{a,j\}}x_j^a S_j^a + B_{\{a,j\},\{b,k\}}x_j^a S_k^b + B_{\{b,k\},\{a,j\}}x_k^b S_j^a + B_{\{b,k\},\{b,k\}}x_k^b S_k^b\Big). \tag{57}$$

As shown before, the mean synaptic weight evolves according to:

$$\begin{aligned}\frac{dJ_{ab}}{dt} = \eta_{ab}\Big(&B_{\{a,j\},\{a,j\}}\Big(\int_{-\infty}^{\infty}\widetilde{K}(f)\langle S_a,S_a\rangle(f)df + \tau_{STDP}r_ar_a\Big)\\ &+ B_{\{a,j\},\{b,k\}}\Big(\int_{-\infty}^{\infty}\widetilde{K}(f)\langle S_a,S_b\rangle(f)df + \tau_{STDP}r_ar_b\Big)\\ &+ B_{\{b,k\},\{a,j\}}\Big(\int_{-\infty}^{\infty}\widetilde{K}(f)\langle S_b,S_a\rangle(f)df + \tau_{STDP}r_br_a\Big)\\ &+ B_{\{b,k\},\{b,k\}}\Big(\int_{-\infty}^{\infty}\widetilde{K}(f)\langle S_b,S_b\rangle(f)df + \tau_{STDP}r_br_b\Big)\Big).\end{aligned} \tag{58}$$

By assumption, $a \neq b$, and re–grouping terms, we get:

$$\begin{aligned}\frac{dJ_{ab}}{dt} = \eta_{ab}\Big(&B_{\{a,j\},\{a,j\}}\tau_{STDP}r_ar_a + (B_{\{a,j\},\{b,k\}} + B_{\{b,k\},\{a,j\}})\tau_{STDP}r_ar_b\\ &+ B_{\{b,k\},\{b,k\}}\tau_{STDP}r_br_b + B_{\{a,j\},\{a,j\}}\int_{-\infty}^{\infty}\widetilde{K}(f)\langle S_a,S_a\rangle(f)df\\ &+ (B_{\{a,j\},\{b,k\}} + B_{\{b,k\},\{a,j\}})\int_{-\infty}^{\infty}\widetilde{K}(f)\mathrm{Re}[\langle S_a,S_b\rangle(f)]df\\ &+ B_{\{b,k\},\{b,k\}}\int_{-\infty}^{\infty}\widetilde{K}(f)\langle S_b,S_b\rangle(f)df\Big).\end{aligned} \tag{59}$$

Grouping the first three terms and the last three terms we obtained:

$$\frac{dJ_{ab}}{dt} = \eta_{ab}\big(\mathrm{Rate}_{a,b} + \mathrm{Cov}_{a,b}\big). \tag{60}$$

Thus rates and spike count covariances come additively into dJ_{ab}/dt. This implies that both rates and covariances determine the location of the fixed point of J_{ab}. Thus, increases in any of the two terms can lead to a shift in the location of the fixed point. In our framework, rates and covariances can be controlled independently through the r_{x}

and c_x, respectively. Therefore, increasing c_x can put the network in regimes where $\mathrm{Rate}_{a,b}$ and $\mathrm{Cov}_{a,b}$ are similar in magnitude and hence increasing covariances can moderately shift the location of the fixed point J_{ab}. (This is the case for Kohonen's rule – see Results in the main text).

Let us now consider a general STDP rule that changes the weights of synapses between neurons of the same population, and these changes are only due to order 2 interactions. In this case, the rates and covariances do not determine the fixed point of the synaptic weights. However, rates and covariances can modulate the speed of convergence of the synaptic weights to steady state.

By assumption, the STDP only involves second order terms. Then:

$$\frac{dJ_{jk}^{ab}}{dt} = \eta_{ab}\bigg(B_{\{a,j\},\{a,j\}}x_j^a S_j^a + B_{\{a,j\},\{b,k\}}x_j^a S_k^b + B_{\{b,k\},\{a,j\}}x_k^b S_j^a + B_{\{b,k\},\{b,k\}}x_k^b S_k^b\bigg). \tag{61}$$

As shown before, the mean synaptic weight evolves according to Eq. (58). Since, $a = b$, and re–grouping terms, we get:

$$\begin{aligned}\frac{dJ_{aa}}{dt} = \eta_{ab}\bigg(B_{\{a,j\},\{a,j\}} + B_{\{a,j\},\{b,k\}} + B_{\{b,k\},\{a,j\}} + B_{\{b,k\},\{b,k\}}\bigg)\times \\ \bigg(\tau_{STDP}r_a^2 + \int_{-\infty}^{\infty}\widetilde{K}(f)\langle S_a, S_a\rangle(f)df\bigg)\end{aligned} \tag{62}$$

Note that

$$\tau_{STDP}r_a^2 + \int_{-\infty}^{\infty}\widetilde{K}(f)\langle S_a, S_a\rangle(f)df > 0 \tag{63}$$

therefore, the only fixed point of J_{aa} is determined by the roots of

$$B_{\{a,j\},\{a,j\}} + B_{\{a,j\},\{b,k\}} + B_{\{b,k\},\{a,j\}} + B_{\{b,k\},\{b,k\}} = 0. \tag{64}$$

Thus the location of the fixed point of J_{ab} is independent of rates and spike count covariances. However, since they come in multiplicatively in the derivative of J_{ab} (*i.e.*, the derivative is proportional to the sum of rate and covariance contributions), they can modulate the speed of convergence of the synaptic weights to the fixed point. This can happen through changes in r_x or c_x.

Supporting results and discussion

Here we include some results that support certain claims made throughout the paper. In particular, we show how the theory breaks down as the assumption of separated timescales is relaxed. We also show that our theory can also describe transient dynamics of synaptic weights fairly well and test if weights reach steady state. Next, we demonstrate mathematically and empirically that weights under Kohonen's rule undergo a saddle–node bifurcation as the parameter β varies. In addition, we analyze distribution of rates, weights, and correlations under Kohonen's, weight–dependent Hebbian, and inhibitory STDP rules. We show that weight–dependent Hebbian leads to a stable balanced state well described by our theory. We then show how one can use our general framework to stabilize dynamics of otherwise unstable rules such as Classical Hebbian STDP. We also show that in weight–dependent Hebbian STDP increasing spike count covariances dramatically increases the speed of convergence to equilbrium, however the general impact of correlations in weights is still small relative to that of

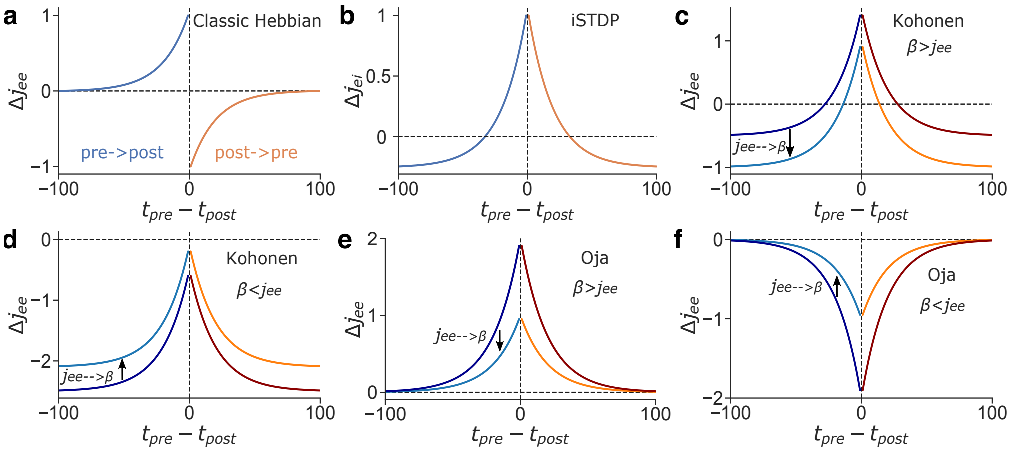

Fig A. STDP windows of different plasticity rules. **a:** Change in synaptic weights as a function of the relative timing of pre– and post–synaptic spikes in Classical Hebbian STDP (same as weight–dependent Hebbian). **b:** Same as **a**, but for inhibitory STDP. **c:** Same as **a**, but for Kohonen's rule when weights are below parameter β. **d:** Same as **c**, but for the case when weights are above β. **e:** Same as **a**, but for Oja's rule when weights are below parameter β. **f:** Same as **e**, but for the case when weights are above β.

rates. Lastly, we provide an example of when synaptic weights can change signs in iSTDP unless the rule is modified, and we show how dynamics of weights undergoing iSTDP in *EI,II* connections can be destabilized at certain plasticity timescales.

What happens when timescales are not separated?

As mentioned in the main text, our theory requires a separation of timescales between spiking dynamics and synaptic weight changes. How are dynamics of balanced networks affected when these timescales are not well separated? Here we consider three example STDP rules: weight–dependent Hebbian, Kohonen, and inhibitory (See Table 1 in main text and Fig. A). We simulate these networks with varying learning rate, and found that when synaptic weights updates occur on a fast timescale, large fluctuations in weights and deviations from theory emerge (Figs. Ba–Bc).

Transient dynamics of synaptic weights

In much of this work we focused on steady state behavior. However, the system of equations (Eqs. 14,21,50) relating rates, covariances, and weights can also be solved iteratively over time, provided the timescales are well separated. Here, we show three examples: weight–dependent Hebbian STDP, Kohonen's rule, and iSTDP. In all three cases, the observed trajectory of synaptic weights closely follows the theoretical predictions (Figs. Ca–Cc).

Determining when synaptic weights reach equilibrium in simulations. To determine if synaptic weights had reached steady state we sampled 1000 synaptic weights from the whole population (of plastic weights) at times $t = 4000$ sec and $t = 5000$ sec (end of simulation). We then compared the two distributions using the Kolmogorov–Smirnov 2–sample test. We found that in the three networks simulated in Figs. F–H, the distributions were not distinguishable, and hence had reached equilibrium by the end of the simulation (Fig. Da–Dc)

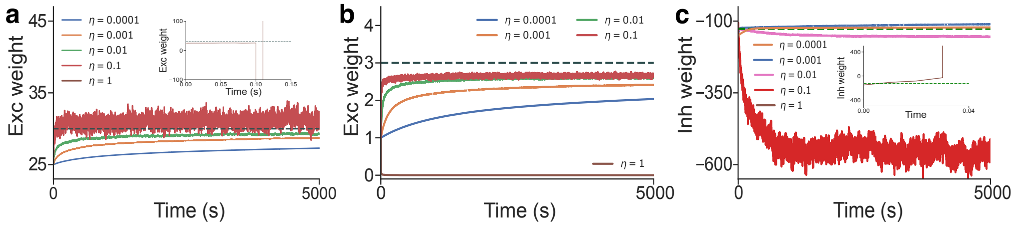

Fig B. Timescale of weight changes in plastic balanced networks. a: Evolution of mean excitatory synaptic weight subject to weight–dependent Hebbian STDP in a balanced network. Fast weight timescales induce large fluctuations and deviations from the fixed point. Inset: When $\eta = 1$ weight updates are so large that the network becomes unstable. **b:** Same as **a:**, but for excitatory weights evolving according to Kohonen's rule. Convergence to the fixed point is very slow when $\eta < 0.01$, and the approximation breaks down for $\eta = 1$. **c:** Same as **a:**, but only inhibitory weights are plastic and follow inhibitory STDP updates. Deviations from the theory appear when timescales are not well separated ($\eta \geq 1/1000$). In all panels, dashed lines represent theory (Eqs. (14,21,66,68), see Table 1 in main text for the equation of mean weights under iSTDP), and solid lines represent numerical results. Relevant simulation parameters: $N = 5000$, $c_{\mathrm{x}} = 0$. Others as in Table A.

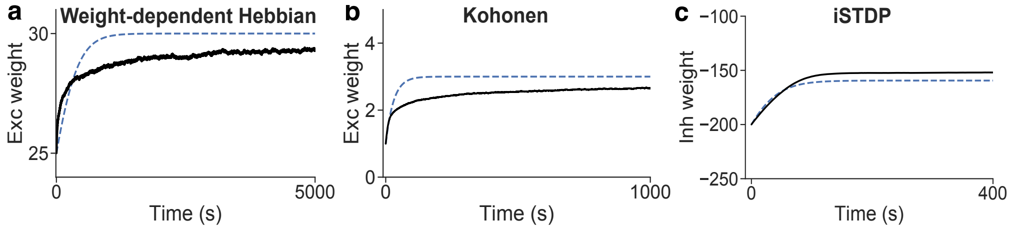

Fig C. Transient dynamics of synaptic weights. a: Mean synaptic weight in a network subject to "weight–dependent" Hebbian STDP. **b:** Same as **a**, but for Kohonen STDP. **c:** Same as **a**, but for inhibitory STDP. In all panels, dashed lines represent the theory (Eqs. (14,21,66,68), see Table 1 in main text for the equation of mean weights under iSTDP); solid lines represent numerical simulations. Simulation parameters as in Figs. F,G,H.

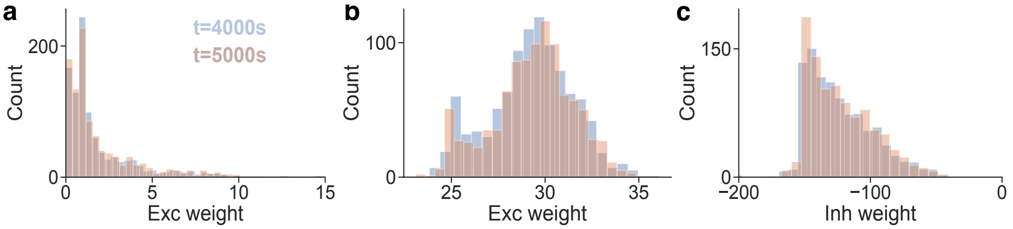

Fig D. Distributions of synaptic weights are stationary. a: Distributions of EE synaptic weights for a balanced network with excitatory weights evolving according to Kohonen's rule at times $t = 4000$sec and $t = 5000$sec. The two distributions are not different according to the Kolmogorov–Smirnov 2–sample test ($D_n = 0.029$, p–value= 0.8). Hence, EE weights are at steady state. **b:** Same as **a**, but for a network with weights that follow weight–dependent Hebbian STDP showing excitatory weights are in equilibrium ($D_n = 0.045$, p–value= 0.26). **c:** Same as **a**, but for a network where EI weights are subject to iSTDP. Inhibitory weights are in equilibrium ($D_n = 0.024$, p–value= 0.94). All simulation parameters as in Figs. F,G,H.

Saddle–node bifurcation of excitatory weights in Kohonen's STDP rule

Assume that EE weights evolve according to Kohonen's STDP rule [16, 17]:

$$\frac{dJ^{\mathrm{ee}}_{jk}}{dt} = \eta_{\mathrm{ee}}\big(\beta x^{\mathrm{e}}_j S^{\mathrm{e}}_k - J^{\mathrm{ee}}_{jk} S^{\mathrm{e}}_j\big). \tag{65}$$

Averaging gives the mean–field dynamics of the excitatory weights:

$$\frac{dJ_{\mathrm{ee}}}{dt} = \eta_{\mathrm{ee}}\left(\beta\tau_{STDP} r_{\mathrm{e}}^2 - J_{\mathrm{ee}} r_{\mathrm{e}} + \beta\int_{-\infty}^{\infty}\widetilde{K}(f)\langle S_{\mathrm{e}}, S_{\mathrm{e}}\rangle df\right). \tag{66}$$

In the asynchronous state, the fixed point of this equation is $J^*_{\mathrm{ee}} = \beta\tau_{\mathrm{STDP}} r_{\mathrm{e}}$.

How does the stability of the synaptic weights depend on β? Our mean-field theory shows that the stable network state disappears in a saddle–node bifurcation (Fig. Ea). For the chosen set of parameters (See Table A), the theory predicts a bifurcation at $\beta \approx 8$ (Fig. Ea). We confirmed the presence of a saddle–node bifurcation in numerical simulations (Fig. Eb). However, the bifurcation occurs at a lower value of β due to divergence of individual synapses (Fig. Ec–Ef). This is not unexpected, as fluctuations can destabilize a system close to a saddle–node bifurcation by pushing it across the unstable fixed point.

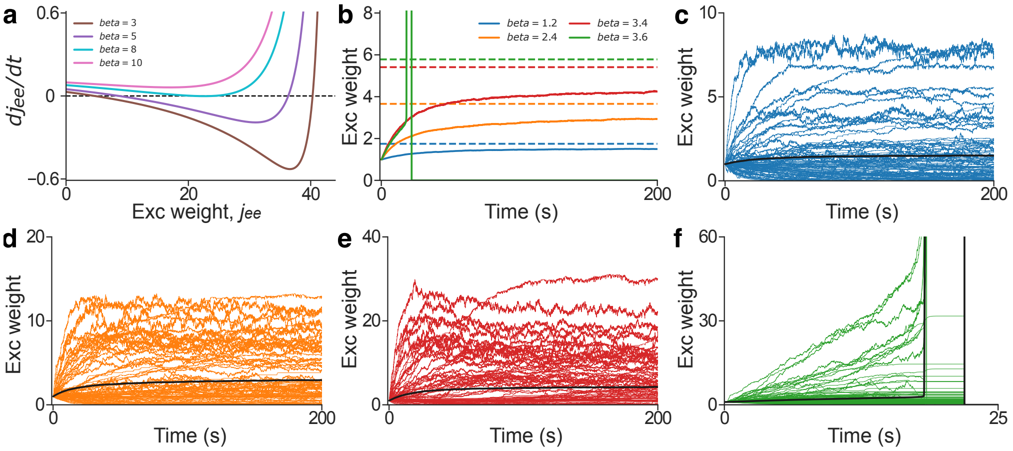

Fig E. Stability of weights in Kohonen's rule. a: The derivative of the mean excitatory weight for different values of excitatory weights subject to Kohonen's STDP rule obtained from Eq. 66. The excitatory weight dynamics undergo a saddle–node bifurcation near $\beta \approx 8$. **b:** Evolution of mean synaptic weights over a range of values of β. A bifurcation occurs in simulations at $\beta \approx 3.6$, as noise pushes the system past the critical point earlier than the mean–field theory predicts. Dashed lines represent theoretical values (Eqs. (14,21,66)), solid lines were obtained from simulations. **c:** Evolution of individual synaptic weights for $\beta = 1.2$. Mean synaptic weight in black. **d:** Same as **c:** for $\beta = 2.4$. **e:** Same as **c:** for $\beta = 3.4$. Variance of individual synaptic weight increases with β. **f:** Same as **c:** for $\beta = 3.6$. The mean, and some individual excitatory weights diverge. In panels **c–f**, solid lines were obtained from numerical simulations. Relevant simulation parameters: $N = 5000$, $c_{\mathrm{x}} = 0$. Others as in Table A.

Statistics and dynamics of balanced networks under pairwise STDP rules

Our theoretical results describe the evolution of averages (See Materials & Methods in main text). However, it is also important to understand changes in higher order

statistics of individual, and collective activity under plasticity. Although we do not go beyond a mean–field theory, we provide the results of corresponding numerical simulations in three example balanced networks where: (1) excitatory weights were subject to weight–dependent Hebbian STDP; (2) EE weights changed according to Kohonen's rule; and (3) inhibitory weights changed according to an iSTDP rule (See Table 1 in main text for equations describing each rule).

Under weight–dependent Hebbian STDP individual synaptic weights converge to a unimodal distribution. This is in agreement with experimental results [18] (Fig. Fa and Fb). Balance is preserved throughout the simulation (Fig. Fc). Distributions of rates are unimodal and skewed to the right (Fig. Fd and inset), also agreeing with experimental findings [18]. Spike count covariances and correlations are distributed around zero in the asynchronous state (Fig. Fe and Ff).

EE weights under Kohonen's rule also converge to a unimodal distribution (Fig. Ga and Gb). Again, the network is stable and balance is preserved (Fig. Gc). The distributions of rates is again unimodal and skewed to the right (Fig. Gd), and covariances and correlations are close to zero (Fig. Ge and Gf). Lastly, EI weights subject to an inhibitory plasticity rule also follow a unimodal distribution (Fig. Ha and Hb), distributions of I rates are unimodal and skewed to the right, and covariances and correlations are centered at zero in the balanced, asynchronous state (Fig. Hc–Hf). However, the E rates are concentrated at the target rate (Fig. Hd inset), in disagreement with experimental findings [18]. In short, all three STDP rules show realistic distributions of weights, rates, and covariances, except for the iSTDP's distribution of E rates.

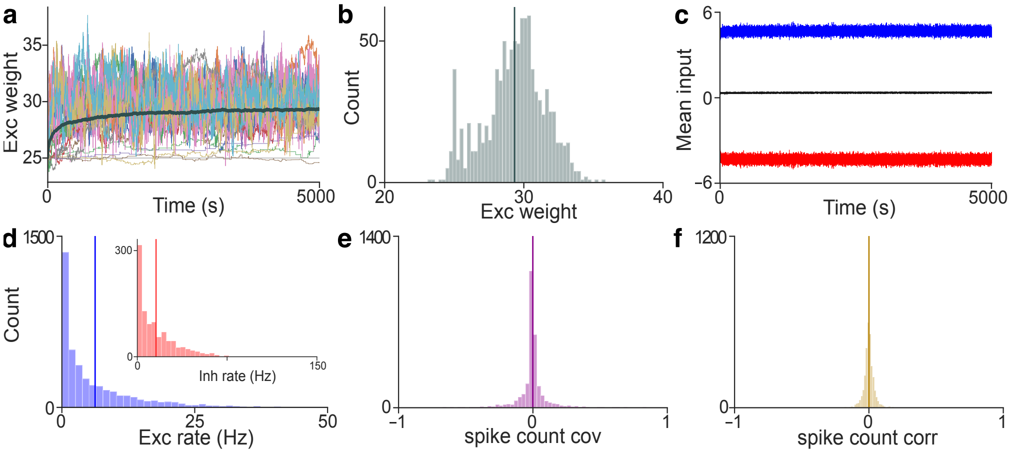

Fig F. Statistics of weights and activity under weight–dependent Hebbian STDP. **a:** Evolution of individual synaptic weights and their mean (black). **b:** EE synaptic weights follow a unimodal distribution at steady state. **c:** Evolution of synaptic currents showing that balance is achieved and maintained. Color scheme as in Fig. 1 in text: blue denotes mean excitatory input, and red the mean inhibitory input. The total mean input is shown in black. **d:** Steady state distribution of excitatory and inhibitory (inset) firing rates. In equilibrium most neurons fire at low rates, and a few neurons fire at high rates. **e:** Distribution of spike count covariances. The mean is close to zero indicating an asynchronous state. **f:** Same as **e:**, but for spike count correlations, again showing a mean that is close to 0. Relevant simulation parameters: $N = 5000$, $c_{\mathrm{x}} = 0$. Others as in Table A.

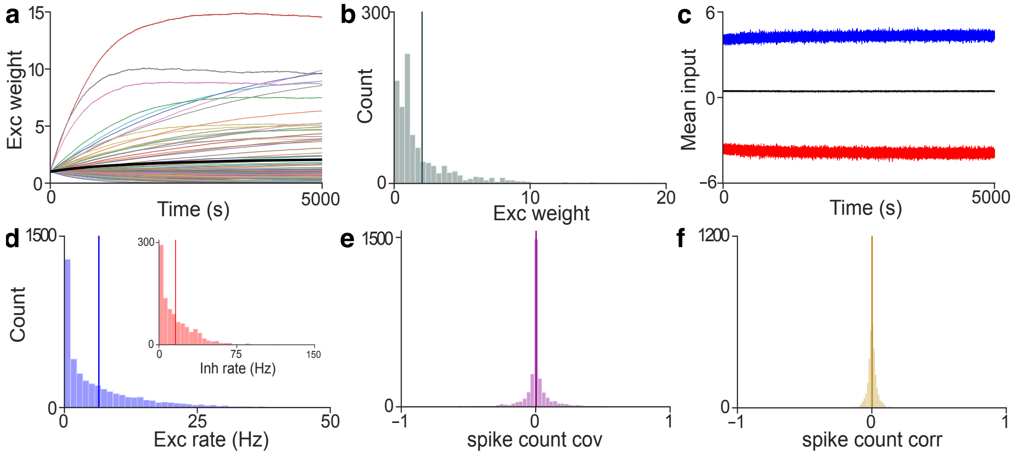

Fig G. Statistics and dynamics of balanced networks undergoing Kohonen's STDP rule. **a:** Evolution of mean synaptic weight subject to Kohonen's STDP rule with mean synaptic weight in black. **b:** The distribution of EE synaptic weights at steady state is unimodal. **c:** Evolution of synaptic currents in the network. Balance is achieved and maintained. **d:** Equilibrium distribution of excitatory and inhibitory (inset) firing rates. Most neurons fire at low rates, and a few neurons fire at high rates. **e:** Distribution of spike count covariances shows that the mean is close to zero (asynchronous state). **f:** Same as **e:**, but for spike count correlations. The mean is close to zero, suggesting that the network is in an asynchronous state. Color scheme and simulation parameters as in Fig. F.

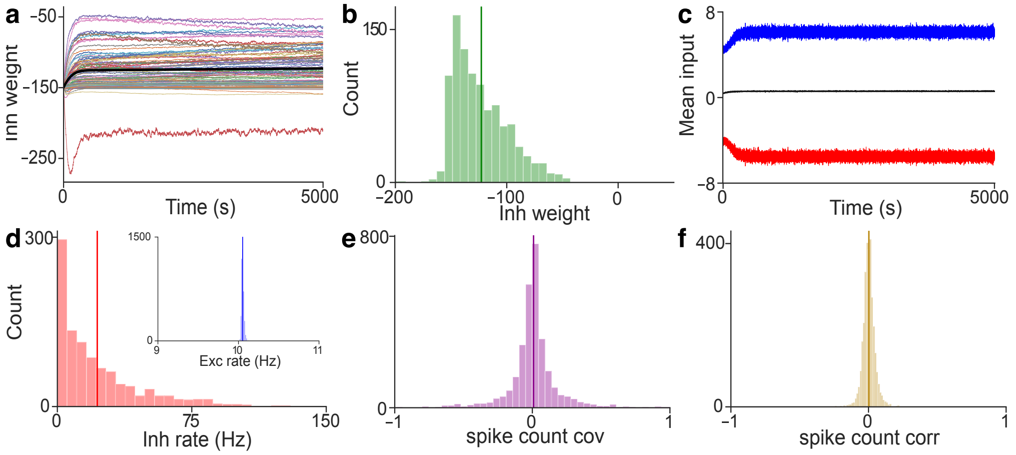

Fig H. Statistics and dynamics of balanced networks experiencing inhibitory STDP. **a:** Evolution of EI synaptic weights subject to iSTDP. Mean synaptic weight in black. **b:** Distribution of EI synaptic weights at steady state. **c:** Evolution of synaptic currents in the network. Balance is achieved and maintained. **d:** Distribution of excitatory (inset) and inhibitory firing rates at equilibrium. Most inhibitory neurons fire at low rates, and a few neurons fire at high rates. All excitatory neurons fire at rates close to the 10Hz target rate. **e:** Distribution of spike count covariances with mean near zero (asynchronous state). **f:** Same as **e:**, but for spike count correlations. The mean is close to zero, suggesting that the network is in an asynchronous state. Color scheme and simulation parameters as in Fig. F.

Asymptotic behavior in weight–dependent Hebbian STDP

Assume that EE synaptic weights evolve according to a Classical Hebbian STDP rule [19–21] (See Table 1 in main text),

$$\frac{dJ_{jk}^{\text{ee}}}{dt} = \eta_{\text{ee}}\left(J_{\max}x_k^{\text{e}}S_j^{\text{e}} - J_{jk}^{\text{ee}}x_j^{\text{e}}S_k^{\text{e}}\right). \tag{67}$$

This rule yields the classical behavior original proposed by Hebb: a presynaptic spike followed by a postsynaptic spike potentiates the synapse. This equation also enforces that a postsynaptic spike followed by a presynaptic spike depresses the synapse.

On average, the EE synaptic weights evolve as:

$$\frac{dJ_{\text{ee}}}{dt} = \eta_{\text{ee}}\left(J_{\max} - J_{\text{ee}}\right)\left(\tau_{\text{STDP}}r_{\text{e}}^2 + \int_{-\infty}^{\infty}\widetilde{K}(f)\langle S_{\text{e}}, S_{\text{e}}\rangle(f)df\right). \tag{68}$$

The fixed point of Eq. (68) is simply $J_{\text{ee}}^* = J_{\max}$ because rate and covariances equations (Eqs. (14,21)) show that the second factor on the right hand side is strictly positive. In any case, we still solve for the firing rates and spike count covariances through their mean-field equations using the fixed point $J_{\text{ee}}^* = J_{\max}$.

Our theoretical framework predicts that the network attains a stable balanced state, and gives the location of the fixed point in terms of weights, rates, and covariances. We confirm our predictions with numerical simulations of the network in a correlated state (Fig. I).

Our theory (Eqs. (14,21,68)) predicted that spike count covariances are order 1 and this is confirmed by numerical simulations (Fig. Ic). As N grows, firing rates and synaptic weights converge to the predicted values (Fig. Ia and Ib).

We have now shown, that for N large enough, the dynamics of a plastic balanced network can be well approximated by our theoretical framework for a range of plasticity rules derived from our general STDP rule, where the weights satisfy the balance condition.

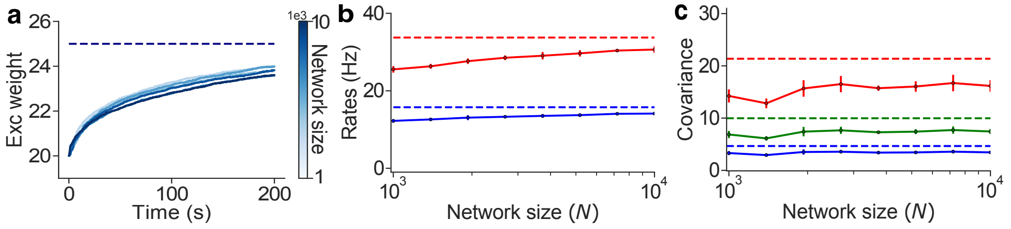

Fig I. Weight–dependent Hebbian STDP yields a stable balanced state. a: Mean excitatory synaptic weight, j_{ee}, evolving over time for increasing network sizes. **b:** Mean E and I firing rates for increasing network sizes. **c:** Mean spike count covariance between E–E, E–I, and I–I spike trains. Dashed lines represent the theory (Eqs. (14,21,68)). Solid lines and dots are obtained from numerical simulations.

Stabilizing Excitatory Hebbian STDP

Classical Hebbian STDP leads to unstable dynamics. If we assume that the EE weights change according to:

$$\frac{dJ_{jk}^{\text{ee}}}{dt} = \eta_{\text{ee}}\left(x_k^{\text{e}}S_j^{\text{e}} - x_j^{\text{e}}S_k^{\text{e}}\right) \tag{69}$$

Mean–field theory predicts stable dynamics when the system has a stable fixed point determined by $dJ_{ee}/dt = 0$. However, simulations show that network dynamics are

unstable due to the divergence of individual weights to $\pm\infty$ (Fig. Ja–Jd), so that the mean stays finite while the variance across the population diverges. Therefore, it is possible that the mean–field theory predicts stable dynamics, despite instabilities in higher moments that can cause weights to blow up. On the other hand, network dynamics will be unstable if the mean-field theory predicts that the first moment diverges.

In simulations Hebbian STDP is frequently stabilized by imposing hard lower and upper bounds on synaptic weights (Fig. Je and Jf). With appropriately chosen bounds, the network is stabilized and balance is achieved (Fig. Jg and Jh). We can use mean–field theory to find alternative ways to modify learning rules in order to stabilize network dynamics: One such modification is to multiply the LTP term in Eq. (69) by a constant ($J_{\max} \sim \mathcal{O}(J_{jk}^{ee})$) and the LTD term by the current value of the synaptic weight, J_{jk}^{ee}, in what we call the "Weight–dependent Hebbian STDP rule" (See Table 1 in main text). This can be interpreted as a constraint on synaptic weights due to limitations on the size and strength of a synapse. Excitatory synaptic weights then converge to a fixed point and the network remains in the balanced regime (Fig. Ji–Jl). This approach can be generalized to other plasticity rules while maintaining the underlying dependence on spike timing.

General impact of correlations in weight dynamics

We have shown that in Kohonen's rule, increasing correlations mildly shifts the location of the fixed point. We now show a different effect of correlations in weight dynamics. Here, we use our theoretical framework to show that in a balanced network with EE weights that change according to Eq. (67), increasing correlations does not affect the location of the fixed point, but increases the speed of convergence to that equilibrium.

As mentioned before, the fixed point of synaptic weights does not change with increasing correlations (Eq. (68)) (Fig. Ka). Since synaptic weights are not affected by these changes in correlations, rates also remain unchanged (Fig. Kb). This is confirmed by numerical simulations. To measure how the speed of convergence is increased by larger correlations, we compute t_{50} which is defined as the time it takes for the mean synaptic weight to reach 50% convergence to the fixed point for increasing values of input correlations, and found that t_{50} is dramatically reduced as correlations become larger, hence the mean weights converge faster and faster to equilibrium (Fig. Kc).

We found that, as predicted in "Remarks on the general STDP rule", the effect of correlations in the weight dynamics is different to that seen in Kohonen's rule. In this case, our theory predicts that increasing covariances (through our parameter c_{x}), speeds up the convergence to equilibrium, while maintaining the location of the fixed point unchanged.

We have used our theory to explore the interaction between weights, rates, and covariances for a network with plastic synapses that followed Kohonen's rule and weight–dependent EE Hebbian STDP, and found that increasing correlations mildly impact the dynamics of the synaptic weights. We would now like to generalize the impact of correlations in the synaptic weights.

To do this, we estimate the covariance terms in our equation for the mean weights (Eq. (50)) and compare them to the rate terms in that same equation. We assume there are interactions of order 2 only, and that these have constant coefficient. In other words, the synaptic weights evolve according to:

$$\frac{dJ_{jk}^{ab}}{dt} = \eta_{ab}\left(x_j^a S_j^a + x_j^a S_k^b + x_k^b S_j^a + x_k^b S_k^b\right) \tag{70}$$

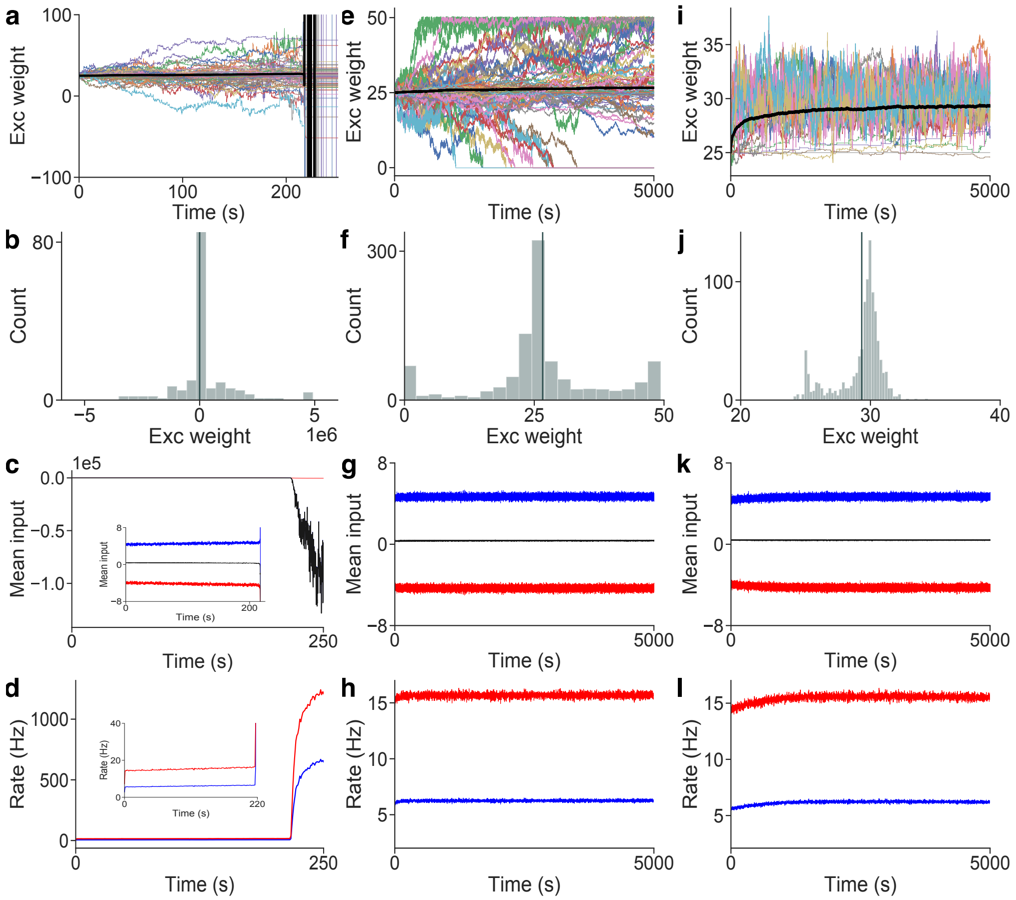

Fig J. Stabilization of Hebbian STDP. a: Excitatory synaptic weights subject to classical Hebbian STDP (See Table 1). The mean synaptic weight is shown in black. Individual weights diverge to $\pm\infty$, destabilizing network dynamics. **b:** Distribution of synaptic weights right before the simulation is terminated. **c:** The mean synaptic input diverges as individual weights diverge. **d:** Positive feedback loops due to unconstrained Hebbian STDP lead to uncontrolled activity in the network. The simulation is terminated when input and rates grow with no bounds. **e–h:** Same as **a–d**, but for Classical Hebbian plasticity with hard constraints at $j_{\mathrm{ee}} = 0$ and $j_{\mathrm{ee}} = 50$. Mean synaptic weight (in black) remains unchanged. Network remains balanced and stable throughout the simulation. **i–l:** Same as **a–d**, but for weight dependent Hebbian STDP (See Table A). The network maintains a stable, balanced state. Relevant simulation parameters: $N = 5000$, $c_{\mathrm{x}} = 0$. Others as in Table A.

where we set all nonzero coefficients equal to one. We then obtain an equation for the mean synaptic weights depending on the rates and covariances:

$$\frac{dJ_{ab}}{dt} = \eta_{ab} \sum_{\alpha,\beta=\{a,b\}} \mathrm{Rate}_{\alpha,\beta} + \mathrm{Cov}_{\alpha,\beta}, \tag{71}$$

where $\mathrm{Rate}_{\alpha,\beta} = \tau_{STDP} r_\alpha r_\beta$, $\mathrm{Cov}_{\alpha,\beta} = \int_{-\infty}^{\infty} \widetilde{K}(f)\langle S_\alpha, S_\beta\rangle(f)df$. The fact that all nonzero coefficients are set to be equal allows us to estimate the raw values of the covariances and rates for each synapses (for $a, b = \mathrm{e}, \mathrm{i}$), and compare them.

We found that for a plasticity rule that satisfies our general STDP rule Eq. (50)

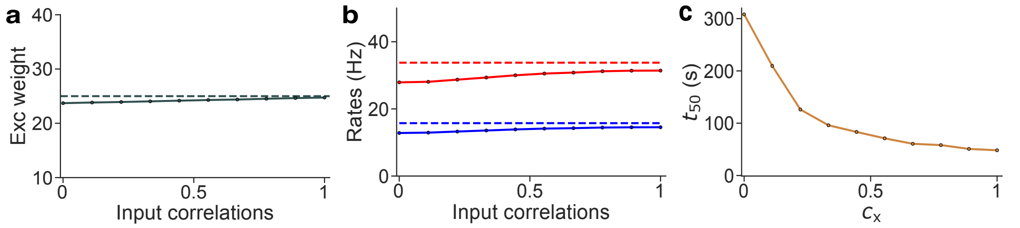

Fig K. Increasing input correlations modulates speed of convergence to equilibrium in weight–dependent Hebbian STDP. **a:** Mean excitatory synaptic weight, j_{ee}, obtained at different values of input correlations. **b:** Mean E and I firing rates for increasing input correlations. **c:** Half time between initial condition and equilibrium for networks with increasing input correlations. Dashed lines represent the theory (Eq. (14,21,68)). Solid lines and dots are obtained from numerical simulations.

acting on *any* synapse, the impact of correlations in the synaptic weights is at least one order of magnitude smaller than the contribution of the rates to the dynamics of the weights (Fig. La–Lc). This implies that changes in the firing rates will usually have a stronger effect on the weights than changes in spike count covariances. However, increasing spike count covariances can still have a mild impact in synaptic weight dynamics when rates remain fixed. This last point is illustrated in our previous examples of networks undergoing excitatory plasticity (Kohonen and Hebbian).

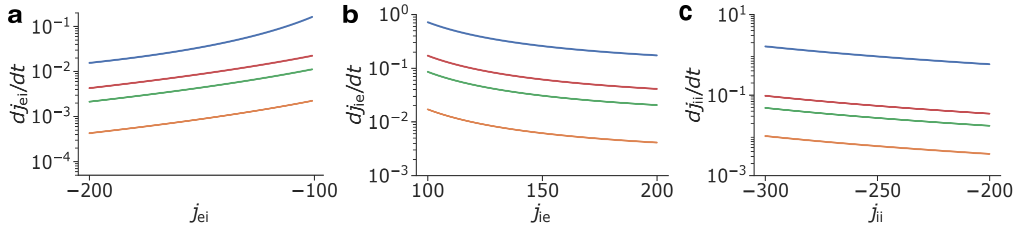

Fig L. General impact of correlations in synaptic weights. **a:** Plot of the time derivative of the mean weight as a function of j_{ei} assuming interactions of order 2 only. **b**, **c:** Same as **a** but for IE and II synapses, respectively. The contribution of rates to the evolution of synaptic weights is always larger than that of the covariances. Solid lines represent the theory (Eqs. (14,21,71)).

Modification to the inhibitory STDP rule

We mentioned that the inhibitory STDP in [15] was slightly modified to include a zero unstable fixed point in the synaptic weights. This was done because under certain relevant conditions, the synaptic weights can change sign in an effort to maintain E rates at the target.

One simple case in which this happens, is when all I neurons are stimulated (Fig. M). The network is put in a semibalanced regime [22] where neurons receive excess inhibition on average (Fig. Ma inset). The inhibitory rates increase, which would effectively decrease E rates. However, I to E synaptic weights decrease such that the E rates are mantained at the target (Fig. Ma and Mb). Since EI weights are not controlled, excess inhibition causes some synapses to switch signs in order to maintain E rates at the target (Fig. Mc). This violates Dale's law, so in all other simulations of inhibitory STDP, the learning rule is modified to incorporate a zero unstable fixed

point, which prevents synaptic weights to switch signs (as proven in "Implementation of inhibitory plasticity in numerical simulations").

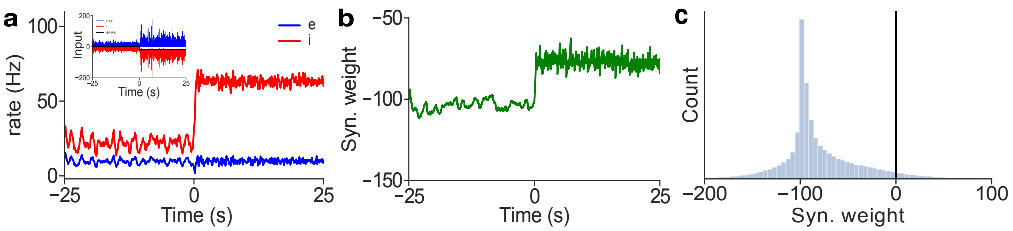

Fig M. Homeostatic nature of inhibitory STDP can break Dale's Law. a: Excitatory and inhibitory firing rates over time. External stimulation starting at time 0 sec increases the firing rate of I cells. Inset: Average excitatory, inhibitory, and total inputs. Total input is negative when I cells are stimulated. **b:** Mean EI synaptic weight over time. After stimulation onset, EI weights decrease in magnitude. **c:** Distribution of EI synaptic weights. A number of inhibitory synapses have turned positive. All plots obtained from a single simulation, where added external input to all inhibitory neurons starts at time 0 sec. $N = 5000$, $c_{\mathrm{x}} = 0$; all other parameters as in Table A.

Stability of iSTDP in EI and II connections

Consider a network with inhibitory STDP in EI and II connections. As we showed in the main text, the theory predicts one stable fixed point for the weights. However, convergence to that stable fixed point depends on individual timescales of synaptic weights. In particular, if $\eta_{\mathrm{ei}} \geq \eta_{\mathrm{ii}}$, then the network realizes a stable balanced state (Fig. Na–Nc and Ng–Ni). However, if EI weights change at least an order of magnitude slower than II weights, then the network is destabilized in attempt to control excitation (Fig. Nd–Nf): Early in the simulation, II weights change fast in an attempt to push I cells to the target rate (Fig. Nd). As a consequence, inhibitory feedback increases and E neurons are silenced (Fig. Ne), since EI STDP works on a slower timescale and is not able to catch up with II STDP to push E rates to the target. At this point, EI STDP makes relatively large updates since the E rates are far from target, and due to its slow timescale, it strongly overshoots and the rates rise very fast past the target. This also increases I rates away from target, and so II weights change very fast to pull I rates towards their target. As a result both E and I rates drop below target (Fig. Ne – first oscillation). The cycle then repeats. These oscillations cause weights and inputs to grow unrealistically large, and push the network out of balance (Fig. Nf).

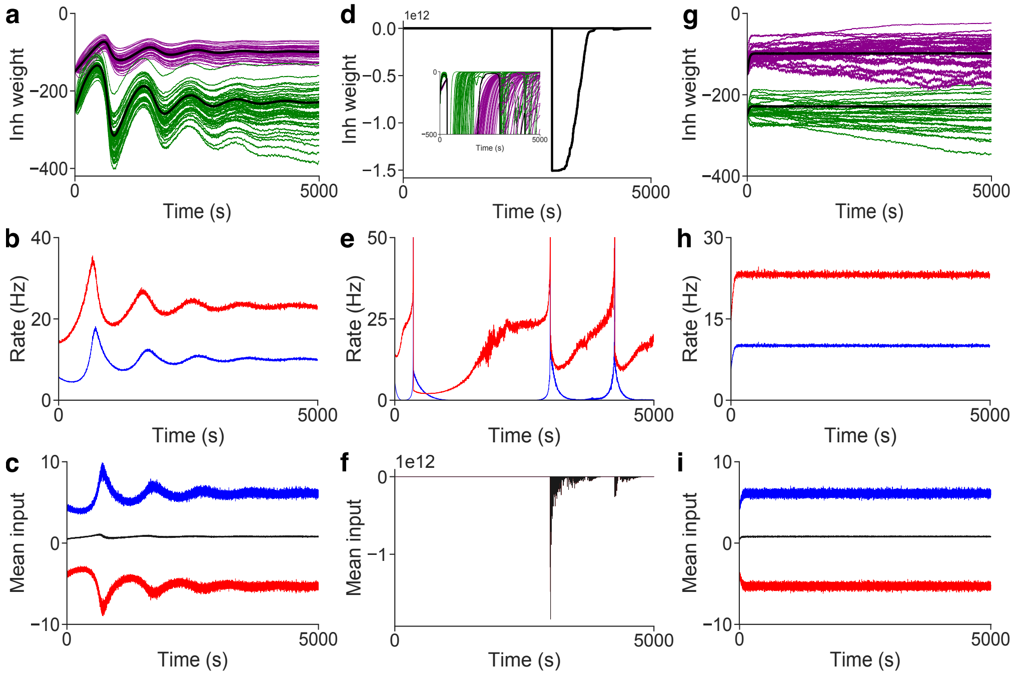

Fig N. Inhibitory STDP: different timescales on *EI*,*II* synapses lead to different behavior. **a:** Individual *EI* (green) and *II* (dark magenta) synaptic weights. Average of each type in black. **b:** Mean firing rates of E and I populations. **c:** Mean excitatory (blue), inhibitory (red) and total (black) input. Network is in balanced state. In **a–c**, $\eta_{\text{ei}} = \eta_{\text{ii}} = 10^{-4}$. **d–f:** Same as **a–c**, but here $\eta_{\text{ei}} = 10^{-4}$ and $\eta_{\text{ii}} = 10^{-3}$. Weights and consequently the mean input grow uncontrollably. Rates oscillate and undergo periods of quiescence of E neurons. **g–i:** Same as **a–c**, but now $\eta_{\text{ei}} = 10^{-3}$ and $\eta_{\text{ii}} = 10^{-4}$. Activity in the network is now balanced and stable. Relevant parameters: $N = 5000$, $c_{\text{x}} = 0$. Other parameters as in Table A.

Technical details of simulations

Neural networks described in the Materials & Methods were simulated numerically using the set of parameters shown in Table A. Code is available at `https://github.com/alanakil/PlasticBalancedNetsPackage`.

Connectivity		
**Parameter**	**Value**	**Description**
p_{ab}	0.1	Probability of connection for all $a, b = \mathrm{e}, \mathrm{i}$
j_{ee}/C_m	25 mV	Weight of E to E synapses
j_{ei}/C_m	−100 mV	Weight of E to I synapses
j_{ie}/C_m	112.5 mV	Weight of I to E synapses
j_{ii}/C_m	−250 mV	Weight of I to I synapses

Neuron Model		
**Parameter**	**Value**	**Description**
C_m	1	Membrane capacitance
g_L	$C_m/15$	Leak conductance
E_L	−72 mV	Resting potential
V_{th}	−50 mV	Spiking threshold
V_{re}	−75 mV	Reset potential
Δ_T	1 mV	'Sharpness' parameter
V_T	−55 mV	Threshold

Plasticity Model		
**Parameter**	**Value**	**Description**
η_{ab}	10^{-4}	Learning rate of synaptic weights
τ_{STDP}	200 ms	Decay constant of eligibility traces
ρ_{e}	10 Hz	Target rate of E cells
ρ_{i}	20 Hz	Target rate of I cells

Table A. Summary of simulation parameters.

References

1. Baker C, Ebsch C, Lampl I, Rosenbaum R. Correlated states in balanced neuronal networks. Phys Rev E. 2019;99:052414.

2. Fourcaud-Trocme N, Hansel D, van Vreeswijk C, Brunel N. How spike generation mechanisms determine the neuronal response to fluctuating inputs. J Neurosci. 2003;23(37):11628–11640.

3. van Vreeswijk C, Sompolinsky H. Chaotic Balanced State in a Model of Cortical Circuits. Neural Computation. 1998;10(6):1321–1371.

4. van Vreeswijk C, Sompolinsky H. Chaos in Neuronal Networks with Balanced Excitatory and Inhibitory Activity. Science. 1996;274(5293):1724–1726.

5. Kuhn A, Aersten A, Rotter S. Higher order statistics of input ensembles and the response of simple model neurons. Neural Comput. 2003;15(1):67–101.

6. Trousdale J, Hu Y, Shea-Brown E, Josic K. Impact of Network Structure and Cellular Response on Spike Time Correlations. PLOS Computational Biology. 2012;8(3):e1002408.

7. Trousdale J, Hu Y, Shea-Brown E, Josic K. A generative spike train model with time-structured higher order correlations. Frontiers in Computational Neuroscience. 2013;7(84):1–21.

8. Renart A, De La Rocha J, Bartho P, Hollender L, Praga N, Reyes A, et al. The Asynchronous State in Cortical Circuits. Science. 2010;327(2):587–590.

9. Gilson M, Burkitt A, Grayden D, Thomas D, van Hemmen J. Emergence of network structure due to spike-timing-dependent plasticity in recurrent neuronal networks. I. Input selectivity-strengthening correlated input pathways. Biol Cybernetics. 2009;101(2):81–102.

10. Gilson M, Burkitt A, Grayden D, Thomas D, van Hemmen J. Emergence of network structure due to spike-timing-dependent plasticity in recurrent neuronal networks. II. Input selectivity-symmetry breaking. Biol Cybernetics. 2009;101(2):103–114.

11. Gilson M, Burkitt A, Grayden D, Thomas D, van Hemmen J. Emergence of network structure due to spike-timing-dependent plasticity in recurrent neuronal networks. III. Partially connected neurons driven by spontaneous activity. Biol Cybernetics. 2009;101(5):411–26.

12. Gilson M, Burkitt A, Grayden D, Thomas D, van Hemmen J. Emergence of network structure due to spike-timing-dependent plasticity in recurrent neuronal networks IV. Biol Cybernetics. 2009;101(5):427–444.

13. Gilson M, Burkitt A, Grayden D, Thomas D, van Hemmen J. Emergence of network structure due to spike-timing-dependent plasticity in recurrent neuronal networks. V: self-organization schemes and weight dependence. Biol Cybernetics. 2010;103(5):365–386.

14. Kempter R, Gerstner W, Van Hemmen J. Hebbian learning and spiking neurons. Physical Review E. 1999;59(4):4498–4514.

15. Vogels TP, Sprekeler H, Zenke F, Clopath C, Gerstner W. Inhibitory Plasticity Balances Excitation and Inhibition in Sensory Pathways and Memory Networks. Science. 2011;334(6062):1569–1573.

16. Kohonen T. Self-Organization and Associative Memory. Springer-Verlag; 1984.

17. Gerstner W, Kistler W, Naud R, Paninski L. Neuronal Dynamics: From single neurons to networks and models of cognition and beyond. Cambridge University Press; 2014.

18. Song S, Sjöström P, Reigl M, Nelson S, Chklovskii D. Highly Nonrandom Features of Synaptic Connectivity in Local Cortical Circuits. PLOS Biology. 2005;3(3):e68.

19. Hebb D. The Organization of Behavior. Wiley; 1949.

20. Markram H, Lübke J, Frotscher M, Sakmann B. Regulation of synaptic efficacy by coincident postsynaptic aps and epsps. Science. 1997;275(5297):213–5.

21. Bi G, Poo M. Synaptic modification of correlated activity: Hebb's postulate revisited. Annu Rev Neurosci. 2001;24(1):139–66.

22. Baker C, Zhu V, Rosenbaum R. Nonlinear stimulus representations in neural circuits with approximate excitatory–inhibitory balance. PLoS Comput Biol. 2020;16(9):e1008192.
