# Supplementary material for: Balanced networks under spike-time dependent plasticity
Source: PLoS Comput Biol. 2021 May 12;17(5):e1008958. doi: 10.1371/journal.pcbi.1008958 (PMC8143429; doi:10.1371/journal.pcbi.1008958)
Supplement: Attachment — Submitted filename: rebuttal_letter_stdp_bns_final.pdf [file pcbi.1008958.s002.pdf]

Summary

We thank the referees for their careful reading of the manuscript and helpful suggestions. Detailed below are modifications made to the manuscript based on the reviewers' comments.

The reviewers made important points about the need to better compare our work with existing literature, as well as better support several claims we make in the manuscript. We have changed and extended the text and figures, and provided additional details in the Supplementary Material to address these concerns. We hope that the updated manuscript is now suitable for publication in PLoS Computational Biology.

Response to Reviewer's General Comments

Reviewer comments are in italics. Our responses are in plain text. New text added to the manuscript is in blue. The corresponding new text in the manuscript is also highlighted in blue.

Editors

As an example, the Introduction currently does not place the work in the context of other published work, and 2/3 of the introduction is basically an extended abstract.

We agree with this comment, and have rewritten the introduction to better relate our work to the existing literature on balanced networks and synaptic plasticity.

> Cortical neuronal activity is typically irregular, correlated, and can be dominated by a low dimensional component [1–6]. Cortical activity is also characterized by a balance between excitation and inhibition [1,7–12]. Such balance is now a widely thought to give rise to stable, irregular neural activity [1,7,12–18]. Early theoretical work has focused on irregular asynchronous dynamics, with large networks exhibiting vanishing correlations [13,19]. However, more recent extensions have shown how correlated dynamics can be generated both endogenously and exogenously, while preserving irregular single cell activity [20–28], showing the existence of both asynchronous and correlated states in balanced networks.
>
> Correlated activity can also produce changes in synaptic weights [29, 30]. For instance spike–time dependent plasticity (STDP), is driven by patterns in the timing of pre– and post–synaptic spikes [31, 32]. However, we still lack a general theory that relates STDP to changes in neural activity, and the resulting neural computations. Hence, much of the theoretical analysis of the effects of STDP relies on simulations [29, 33–35]. Analytical treatments have been proposed for a number of cases, initially describing the impact of STDP on

individual synaptic weights [36]. These results have been extended to small networks [34], and networks of Poisson neurons [37–40]. Other work provided analytical treatments of specific plasticity rules, such as homeostatic inhibitory plasticity [41, 42]. Using linear response and motif resuming techniques [43], Ocker et al. developed a theory describing the evolution of mean weights in recurrent neural networks of noisy integrate–and–fire neurons under STDP [44]. This approach relies on the assumption that the input to individual cells is dominated by white noise and that local synaptic input is comparatively weak. Related results were obtained by treating neural firing as a Poisson process [37–39, 45].

Here, we develop a complementary theory describing the evolution of synaptic weights and associated mean rates in tightly balanced networks in both correlated and asynchronous states. We combine the mean–field theory of firing rates and correlations in balanced networks [13, 14, 23, 24, 46–48] with an averaging approach assuming a separation of timescales between changes in spiking activity, and the evolution of synaptic weights [30]. We show how the weights and the network dynamics co–evolve under different classical rules, such as Hebbian plasticity, Kohonen's rule, and a form of inhibitory plasticity [31, 32, 41, 49, 50]. In general, the predictions of our theory agree well with empirical simulations. We also explain when the mean–field theory fails, leading to disagreements with simulations, and we develop a semi–analytic extension of the theory that explains these disagreements. Our theory thus allows us to understand mean synaptic weight dynamics, and predicts how synaptic plasticity impacts network dynamics.

We find that spike train correlations, in general, have a mild effect on the synaptic weights and firing rates, in agreement with previous work [44, 51]. We also show that for some STDP rules, synaptic competition can introduce correlations between synaptic weights and firing rates, resulting in the formation of a stable manifold of fixed points in weight space, and hence asymptotic weight distributions that depend on the initial state. Finally, we apply this theory to show how inhibitory STDP [41] can lead to a reestablishment of an asynchronous, balanced state that is broken by optogenetic stimulation of a neuronal subpopulation [52]. We thus extend the classical theory of balanced networks to understand how synaptic plasticity shapes their dynamics.

Referee A

Akil et al. develop a mean field theory (with some important semi analytical corrections) for the co-evolution of synaptic weights and first and second order firing rate statistics in spiking neuronal networks. This is potentially an important contribution to both theoretical and experimental neuroscience, where the understanding of the effects various

plasticity rules have at the network level are lacking. This paper puts our understanding of these effects on more firm analytical grounds. However, I think that the results could be strengthened significantly if the authors leverage the theory they developed to address more fundamental problems than the applications included in the current version. Beyond that, the current version of the manuscript is missing some key references to and discussion of both theoretical and experimental literature that would help readers understand the importance of the results and their limitations.
A fundamental problem in theoretical neuroscience is the stability of neuronal network activity subject to activity dependent plasticity. It is clear–and the authors mention it in their paper (l. 217)– that Hebbian excitatory plasticity leads to instabilities. The theory that the authors develop is exactly the analytical tool needed to understand what are minimal and/or realistic modifications of this rule which do lead to stable dynamics. More specifically– can the reduced, mean-field, description of the network+plasticity rule be leveraged to understand under what circumstances a Hebbian EE STDP rule could be stabilized? Alternatively, are there families of plasticity rules which are guaranteed to not stabilize the dynamics?

Thank you for the comments. We have now expanded the Discussion and the Appendix to bring out these points better. The following text now appears in the Discussion:

> One of the most important issues in understanding neural dynamics is establishing conditions under which the network remains active, yet stable as synaptic weights change. The theory we developed can help us address this questions, but it does have limitations. Since we used a mean–field approach, we can only capture first moments. While mean weight stability may not imply stable network dynamics (consider the case when weight variance diverges in Classical Hebbian STDP in S1 Appendix), instability in the mean weights does imply that the network is also unstable.

We have also expanded further on these results in S1 Appendix.

> **Classical Hebbian STDP leads to unstable dynamics.** If we assume that the EE weights change according to:
>
> $$\frac{dJ^{\mathrm{ee}}_{jk}}{dt} = \eta_{\mathrm{ee}}\left(x^{\mathrm{e}}_k S^{\mathrm{e}}_j - x^{\mathrm{e}}_j S^{\mathrm{e}}_k\right) \tag{1}$$
>
> Mean–field theory predicts stable dynamics when the system has a stable fixed point determined by $dJ_{ee}/dt = 0$. However, simulations show that network dynamics are unstable due to the divergence of individual weights to $\pm\infty$ (Supporting Fig. 10 **A–D**), so that the mean stays finite while the variance across the population diverges. Therefore, it is possible that the mean–field theory predicts stable dynamics, despite instabilities in higher moments that can cause weights to blow up. On the other hand, network dynamics will be unstable if the mean-field theory predicts that the first moment diverges.

In simulations Hebbian STDP is frequently stabilized by imposing hard lower and upper bounds on synaptic weights (Supporting Fig. 10 **E,F**). With appropriately chosen bounds, the network is stabilized and balance is achieved (Supporting Fig. 10 **G,H**). We can use mean–field theory to find alternative ways to modify learning rules in order to stabilize network dynamics: One such modification is to multiplying the LTP term in Eq. (1) by a constant ($J_{\max} \sim \mathcal{O}(J^{ee}_{jk})$) and the LTD term by the current value of the synaptic weight, J^{ee}_{jk}, in what we call the "Weight–dependent Hebbian STDP rule" (See Table 1 in main text). This can be interpreted as a constraint on synaptic weights due to limitations on the size and strength of a synapse. Excitatory synaptic weights then converge to a fixed point and the network remains in the balanced regime (Supporting Fig. 10 **I–L**). This approach can be generalized to other plasticity rules while maintaining the underlying dependence on spike timing.

Does EE STDP combined with a specific form of inhibitory plasticity to do this job? (similar to the numerical work in Litwin–Kumar Doiron 2014 which is mentioned in the discussion)?

This is a good point, and we have now addressed it in the following paragraph that we added to the Discussion:

As we mentioned, our theory can be used to show that small modifications to weight updates can stabilize different STDP rules. The question remains whether Hebbian EE plasticity can be stabilized through an interaction with STDP rules at different synapses? For instance, Litwin–Kumar and Doiron used a triplet voltage STDP rule that was stabilized by hard constraints and weight normalization to produce network assemblies [35]. This rule by itself lead to stable but pathological behavior, and they introduced iSTDP to restore a balanced, asynchronous network state. While such voltage–based triplet rules are outside the scope of the present study, we could use extensions of the mean–field theory to describe the impact of second and higher order moments on the evolution of weights, and network dynamics [53]. Our theory suggests that the classical pairwise Hebbian STDP cannot be stabilized by other STDP rules such as iSTDP.

Kohonen's rule that the authors investigate is stable– but I am not aware of this rule having an electrophysiological basis. The authors do not cite literature to that effect, and so I do not agree that the current results support their statement (l. 440): "Therefore, this framework provides mathematical tractability and biological relevance to a model of excitatory STDP [25], without the need for unrealistically fast inhibitory STDP."

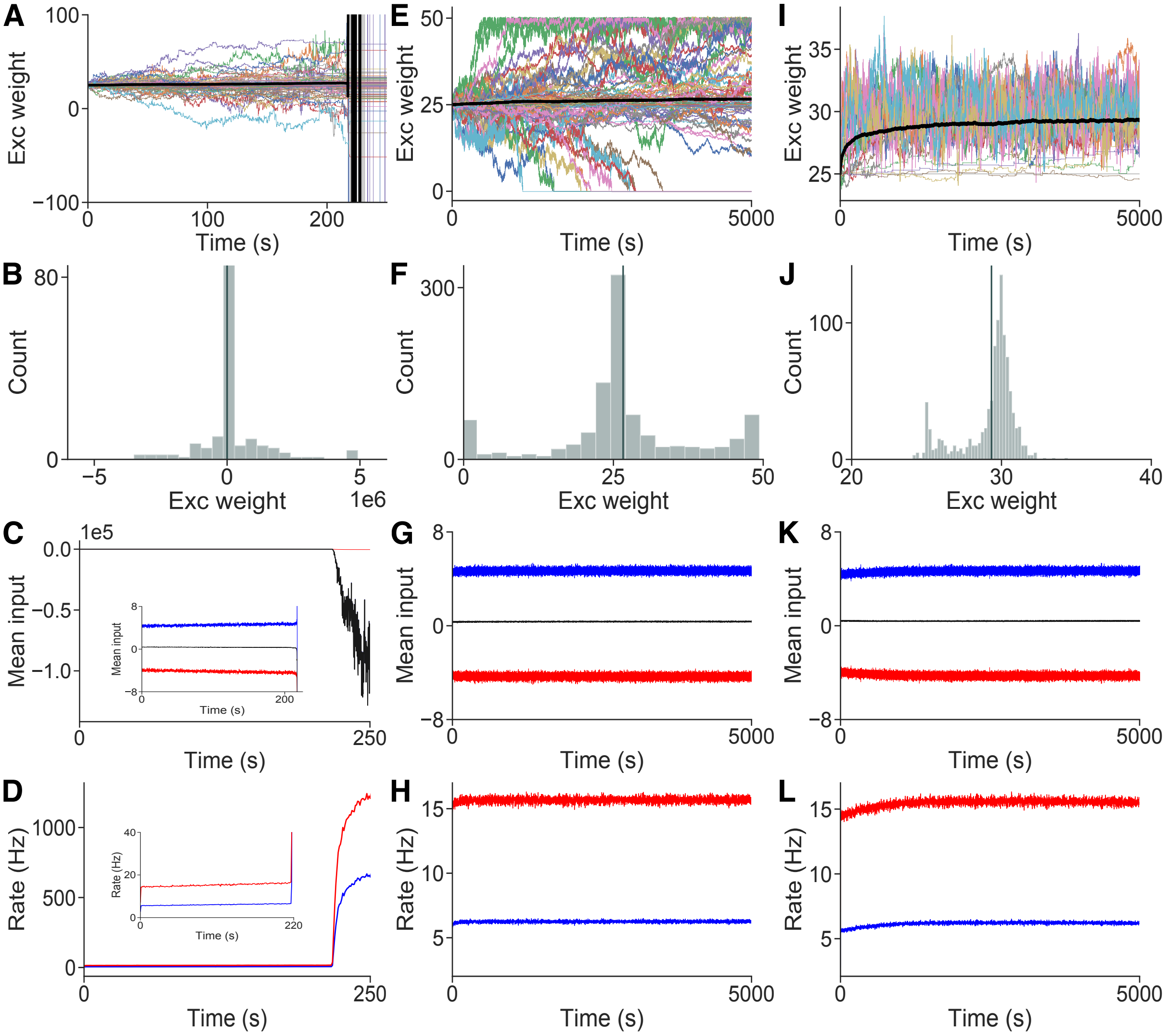

Supporting Figure 10: **Stabilization of Hebbian STDP. A:** Excitatory synaptic weights subject to classical Hebbian STDP (See Table 1). The mean synaptic weight is shown in black. Individual weights diverge to $\pm\infty$, destabilizing network dynamics. **B:** Distribution of synaptic weights right before the simulation is terminated. **C:** The mean synaptic input diverges as individual weights diverge. **D:** Positive feedback loops due to unconstrained Hebbian STDP lead to uncontrolled activity in the network. The simulation is terminated when input and rates grow with no bounds. **E:** Same as **A–D**, but for Classical Hebbian plasticity with hard constraints at 0 and 50. Mean synaptic weight (in black) remains unchanged. Network remains balanced and stable throughout the simulation. **F:** Same as **A–D**, but for weight dependent Hebbian STDP (See Table 1). The network maintains a stable, balanced state.

We agree with this comment. Our point here was to highlight the mathematical tractability of a *nontrivial* excitatory STDP rule such as Kohonen's. We are also not aware of electrophysiological evidence of such a rule, although it has been argued that it is biolog-

ically plausible [54]. However, we still highlight generally desirable features of this rule such as stability and competition. We have now added the following statement to the revised manuscript.

> Therefore, this framework allows us to treat analytically a nontrivial, plausible model of excitatory STDP [50, 54–56], without the need for unrealistically rapid compensatory processes (RCP's) [57, 58].

108 "only pairwise interactions are meaningful". Please clarify what "meaningful" means here, since a few lines afterwards the authors say they will consider more general examples in the future (115 "the BCM rule... depend on interactions beyond second order ..."). Another class of plasticity rules that cannot immediately be written in terms of the current theory are calcium based rules (Shouval et al 2002, Graupner Brunel 2012)

We agree that this statement was unclear, and have rephrased it. What we meant is that in our approach only pairwise interactions are accounted for. As the reviewer points out, higher order interactions can be essential in determining changes in synaptic weights. We have now rephrased this statement in the new version:

> Higher order interactions are at the heart of triplet rules [59–61], and other types of interactions may also be important, *e.g.* for calcium–based update rules [62, 63]. For simplicity we here focus on pairwise interactions between spikes and eligibility traces, and leave extensions to more complex rules for future work.

124 Timescale assumption. The separation of timescales assumption should be introduced more clearly, on a number of levels:

1. *Some readers may appreciate being given approximate values for the different timescales in the theory, perhaps in relation to the specific examples in Table 1.*

   We have now extended our analysis to better illustrate the importance of the separation of timescales in different STDP rules. This is now shown in the section entitled "Separation of timescales" in the Appendix. We also agree that it will be useful for readers to see the actual values of different timescales and we have now included this in the text.

   > We used $1/\eta_{ab} = 10^5 ms$, and $\tau_{\text{STDP}} = 200ms$, with correlation time window width $T_{\text{win}} = 250ms$. Our derivations require $\tau_{\text{STDP}} \ll \Delta T \ll 1/\eta_{ab}$, however an exact numerical value for ΔT is neither used nor needed.

2. *The authors should discuss whether these assumptions are consistent with available data. For example Petersen, Malenka, ... Hopfield 1998, Froemke, Tsay, et al 2006, where 10 repeats of a plasticity protocol are sufficient to induce significant changes or even saturation.*

   The precise agreement can depend on the example. We used values for which the analytical expression agreed with numerical simulations qualitatively and quantitatively. As the separation of timescales is relaxed, both quantitative and qualitative agreements break down as the network becomes unstable due to increased fluctuations in the weights (See new Supplementary Figure 2). We now address this point in the Discussion.

   > The present theory relies on a separation of timescales between spiking dynamics and weight changes. Such timescale separation is supported by a number of experiments [30–32, 58, 64]. We show in the Appendix (see "S1 Appendix: What happens when timescales are not separated?") that reducing this timescale separation, and increasing weight updates leads to a breakdown of the theory, and can result in network instability.
   >
   > In mammalian brains, timescales of weight changes may not always be separated from rate and correlation timescales. The size and timescale of weight updates is likely to depend on many factors that can modulated STDP, such as spiking patterns, synapses type, brain area, network state, neuromodulation, and others. Separation of timescales may not be pronounced in certain non–cortical areas, such as the hippocampus, which can be rapidly modified [58]. For example, Petersen et al., 1998 and Froemke et al., 2006 found significant changes in putative synaptic weights over short timescales in hippocampal CA1/CA3 slices [65] and in visual cortical slices subject to multispike pre–and post–synaptic bursts [66], respectively. However, it is possible that the rate of change of synaptic weights may be overestimated *in vitro* [58].

3. *some computational studies indicate that the separation of timescales assumption must be violated for network stability, at least in relation to homeostatic plasticity (Zenke Gerstner Ganguli 2017). Does the theory provide a path for getting around these issues?*

   This is a good point to raise that needed clarification. We have added a paragraph addressing this in the Discussion.

   > How is our separation of timescales assumption affected when rapid compensatory processes are needed for homeostasis, given that experiments

show that homeostasis is a process that is even slower than the timescale of STDP? Experimental evidence suggests that homeostatic processes can take hours or days [57,58,64]. On the other hand, theoretical models show that synaptic plasticity can be unstable in the presence of such slow homeostasis, and needs to be coupled with rapid compensatory processes such as inhibitory STDP [57, 58]. The separation of timescales in our theory still puts synaptic dynamics on the "fast" side of the spectrum, as it separates network dynamics that occur over milliseconds from weight dynamics that take place over seconds or minutes. Hence, the assumption of timescale separation is still valid in our implementation of homeostatic inhibitory plasticity.

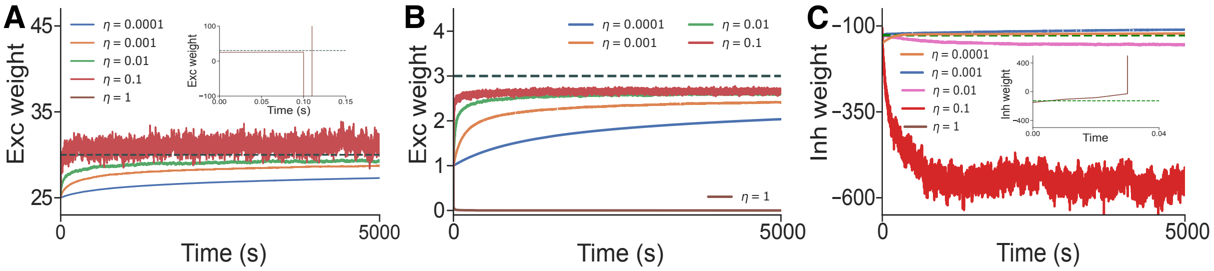

Supporting Figure 2: **Timescale of weight changes in plastic balanced networks.** **A:** Evolution of mean excitatory synaptic weight subject to weight–dependent Hebbian STDP in a balanced network. Fast weight timescales induce large fluctuations and deviations from the fixed point. Inset: When $\eta = 1$ weight updates are so large that the network becomes unstable. **B:** Same as **A:**, but for excitatory weights evolving according to Kohonen's rule. Convergence to the fixed point is very slow when $\eta < 0.01$, and the approximation breaks down for $\eta = 1$. **C:** Same as **A:**, but only inhibitory weights are plastic and follow inhibitory STDP updates. Deviations from the theory appear when timescales are not well separated ($\eta \geq 1/1000$). In all panels, dashed lines represent theory (Eqs. (3,5,12)), and solid lines represent numerical results.

154 Intuitively I expect Neumann's approximation to break down for sparse matrices because the inverse $\overline{W}_0^{-1}$ *is not well defined when the rank is not full. Is the 10% connectivity sufficiently large to avoid this issue? If yes, how small can the connection probability be before the approximation (and consequently the theoretical analysis) breaks down? Guzman, Schlogl, et al 2016 showed 1% connectivity in hippocampal region CA3– a region where recurrent connectivity and plasticity are thought to play important roles. The authors should comment on whether they expect their theory to be applicable in such a scenario.*

This is a good point. It is crucial to keep in mind the difference between J (full connectivity matrix of size $N \times N$, sparse) and $\overline{W}$ (mean–field connectivity matrix of size 2×2, full). We only assumed that $\overline{W}$ is invertible, which is generically true because $\overline{W}$ is full.

Previous work has considered mean–field theory of balance with multiple populations. In such cases $\overline{W}$ larger than 2×2, but still not sparse. In such cases it was shown that a singular connectivity matrix, $\overline{W}$, can break balance. In the cases we consider, our assumption that $\overline{W}$ is non-singular is generic. We clarified these points with the following paragraph:

> The 2×2 mean–field connectivity matrix, $\overline{W}_0$, must be non-singular for the balanced state to exist and for Neumann's approximation to hold [13]. While the non–singularity of $\overline{W}_0$ is a non–restrictive condition for two neural populations, $\overline{W}_0$ can become singular in some models with several neural subpopulations [47, 52].

158 ΔW can be defined such that the perturbation is done separately on EE, EI, IE, II connections. Does doing so within the current theory lead to the same results as Mongillo, Rumpel, Loewenstein 2018?

Mongillo et al., 2018 describe the remarkable stability of firing rate patterns to perturbations in excitatory, but not inhibitory synaptic weights. To understand the reason behind this observation they developed a numerical and theoretical approach to study the impact of *randomly* perturbing EE, EI, IE, and II weights. The fact that these perturbations are randomly generated, and not activity driven, is an important difference between their study and ours: While Mongillo et al. were agnostic about the particular rules that change the weights, we focused on modeling particular rules that drive changes in synaptic weights.

This difference in the approaches also makes it difficult to compare our results directly. For instance, the effect discussed by Mongillo et al. would likely be even stronger under the iSTDP rule that we discuss in the manuscript: The sensitivity of activity patterns to a change (rewiring in their case) of I-to-I and I-to-E, but not E-to-E connections is a result of the wider distribution of the I firing rates. Since the effect of iSTDP in our case is to reduce the variance in E firing rates further, we would expect such difference in sensitivity to be amplified in our network. On the other hand, applying the iSTDP rule to II synapses would reduce the distribution of I firing rates, as we discuss in the manuscript. We expect that this in turn would increase the sensitivity of firing rate patterns to perturbations in II synapses. It is possible that other plasticity rules, and their combination increase the variance of firing rates in either or both population resulting in different changes in sensitivity to perturbations. The answer to this question therefore depends on the learning rules, and their effect on the patterns of activity in the network.

We now mention this point in the Discussion where we write:

> We have shown that different plasticity rules can result in distinct firing rate distributions in different subpopulations. As shown by Mongillo et al. this can result in an increase or decrease in sensitivity of activity patterns and memories to perturbation of different synapse classes [67].

170 The writing in the first results paragraph should be improved to clarify this is an outline of the rest of the paper. Currently the different sections and applications of the theory come as a surprise. Otherwise I like the structure of the paper where introduction is mixed with development of the theory.

This is a good point. We have now rewritten this paragraph to make it clear that this is just an overview of the remainder of the paper.

> In the following sections, we use the theory described in the Methods to show how synaptic weights and correlated activity co–evolve in tightly balanced networks. We first apply our theory to a balanced network with plastic excitatory synapses that evolve according to Kohonen's STDP rule [50]. The theory predicts that the network is in a stable, balanced state, and provides asymptotic values for rates, spike count covariances, and synaptic weights. We also show that correlated activity can have a moderate impact on the dynamics of synaptic weights. Next we show that the theory does not accurately predict weight dynamics for certain STDP rules for which heterogeneity in rates can lead to competition between synaptic weights. The resulting correlations between rates and weights are inconsistent with classical balanced network theory. We show how our framework can be extended to describe such networks. Finally, we demonstrate that our theory can describe the robust response of iSTDP to targeted optogenetic input to subsets of neurons.

212 How does the stability depend on beta?

This is an important question that we have now addressed in the Appendix. Under Kohonen's rule, the dynamics undergo a saddle–node bifurcation as β increases. We have now included these results in "S1 Appendix: Saddle–node bifurcation of excitatory weights in Kohonen's STDP rule", where we show the presence of a saddle–node bifurcation theoretically and confirmed it with simulations.

> **Saddle–node bifurcation of excitatory weights in Kohonen's STDP rule.** Assume that EE weights evolve according to Kohonen's STDP rule [50, 56]:
>
> $$\frac{dJ_{jk}^{\mathrm{ee}}}{dt} = \eta_{\mathrm{ee}}\big(\beta x_j^{\mathrm{e}} S_k^{\mathrm{e}} - J_{jk}^{\mathrm{ee}} S_j^{\mathrm{e}}\big). \tag{2}$$
>
> Averaging gives the mean–field dynamics of the excitatory weights:
>
> $$\frac{dJ_{\mathrm{ee}}}{dt} = \eta_{\mathrm{ee}}\left(\beta\tau_{STDP} r_{\mathrm{e}}^2 - J_{\mathrm{ee}} r_{\mathrm{e}} + \beta\int_{-\infty}^{\infty} \widetilde{K}(f)\langle S_{\mathrm{e}}, S_{\mathrm{e}}\rangle df\right). \tag{3}$$
>
> In the asynchronous state, the fixed point of this equation is $J_{\mathrm{ee}}^{*} = \beta\tau_{\mathrm{STDP}} r_{\mathrm{e}}$.

How does the stability of the synaptic weights depend on β? Our mean-field theory shows that the stable network state disappears in a saddle–node bifurcation (Supporting Fig. 5 **A**). For the chosen set of parameters (See Supporting Table 1), the theory predicts a bifurcation at $\beta \approx 8$ (Supporting Fig. 5 **A**). We confirmed the presence of a saddle–node bifurcation is confirmed in numerical simulations (Supporting Fig. 5 **B**). However, the bifurcation occurs at a lower value of β due to divergence of individual synapses (Supporting Fig. 5 **C–F**). This is not unexpected, as fluctuations can destabilize a system close to a saddle–node bifurcation by pushing it across the unstable fixed point.

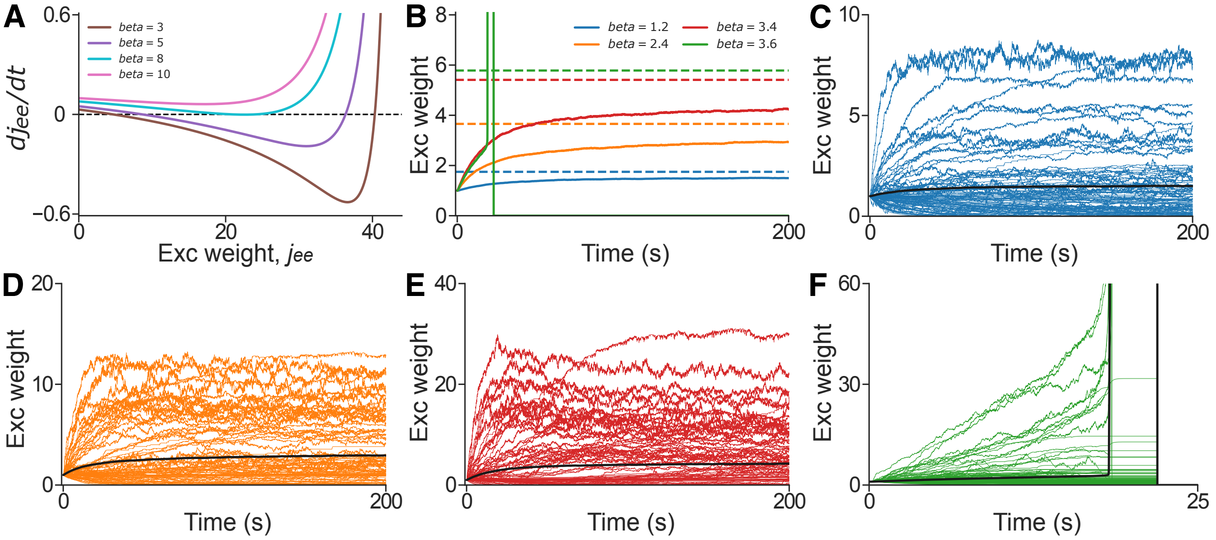

Supporting Figure 5: **Stability of weights in Kohonen's rule.** **A:** The derivative of the mean excitatory weight for different values of excitatory weights subject to Kohonen's STDP rule obtained from Eq. 3. The excitatory weight dynamics undergo a saddle–node bifurcation near $\beta \approx 8$. **B:** Evolution of mean synaptic weights over a range of values of β. A bifurcation occurs in simulations at $\beta \approx 3.6$, as noise pushes the system past the critical point earlier than the mean–field theory predicts. Dashed lines represent theoretical values, solid lines were obtained from simulations. **C:** Evolution of individual synaptic weights for $\beta = 1.2$. Mean synaptic weight in black. **D:** Same as **C:** for $\beta = 2.4$. **E:** Same as **C:** for $\beta = 3.4$. Variance of individual synaptic weight increases with β. **F:** Same as **C:** for $\beta = 3.6$. The mean, and some individual excitatory weights diverge. In panels **C–F**, solid lines were obtained from numerical simulations.

Fig. 1 B, C: are these rasters of only E neurons? what about I?

Yes, they were only E cells. We have now included rasters of the full population color coding I cells as red and E cells as blue.

Fig. 1 D–I: what do the distributions of weights/rates/covariances look like? Kohonen's

rule has the advantage that synaptic weights do not have a hard upper bound. Rather, β plays the role of a soft upper bound. So the networks in these simulations have the potential of maintaining a realistic unimodal synaptic weight distribution, as seen in experiments, in contrast to previous theoretical studies where synaptic weights quickly go to their upper and lower bound (e.g. Rubin Lee Sompolinsky 2001). Is this indeed the case in these simulations?

The authors should indicate and discuss whether their simulations capture the corresponding experimentally observed properties of plastic neuronal networks beyond the averages.

We have now included more statistics and shown the dynamics of different realizations of networks under plasticity rules such as Kohonen's, iSTDP, and weight–dependent Hebbian in Supplementary Figs. 6–8. For the case of Kohonen's rule, we have shown this in "S1 Appendix: Statistics and dynamics of balanced networks undergoing pairwise STDP rules". The *EE* weights in Kohonen's rule follow a unimodal distribution. Distributions of rates are unimodal and asymmetric (Supporting Fig. 7 **A–B**) as seen in experiments. The distribution of covariances and that of correlations are unimodal centered close to zero in the asynchronous state (Supporting Fig. 7 **E,F**). We have added the following section to "S1 Appendix: Statistics and dynamics of balanced networks under pairwise STDP rules", and have included distributions of rates, covariances, correlations, and weights for networks undergoing weight–dependent Hebbian STDP, Kohonen's STDP, and iSTDP:

> **Statistics and dynamics of balanced networks under pairwise STDP rules.** Our theoretical results describe the evolution of averages (See Methods in main text). However, it is also important to understand changes in higher order statistics of individual, and collective activity under plasticity. Although we do not go beyond a mean–field theory, we provide the results of corresponding numerical simulations in three example balanced networks where: (1) excitatory weights were subject to weight–dependent Hebbian STDP; (2) *EE* weights changed according to Kohonen's rule; and (3) inhibitory weights changed according to an iSTDP rule (See Table 1 in main text for equations describing each rule).
>
> Under weight–dependent Hebbian STDP individual synaptic weights converge to a unimodal distribution. This is in agreement with experimental results [68] (Supporting Fig. 6 **A,B**). Balance is preserved throughout the simulation (Supporting Fig. 6 **C**). Distributions of rates are unimodal and skewed to the right (Supporting Fig. 6 **D** and inset), also agreeing with experimental findings [68]. Spike count covariances and correlations are distributed around zero in the asynchronous state (Supporting Fig. 6 **E,F**).
>
> *EE* weights under Kohonen's rule also converge to a unimodal distribution (Supporting Fig. 7 **A,B**). Again, the network is stable and balance is preserved (Supporting Fig. 7 **C**). The distributions of rates is again unimodal

and skewed to the right (Supporting Fig. 7 **D**), and covariances and correlations are close to zero (Supporting Fig. 7 **E,F**). Lastly, EI weights subject to an inhibitory plasticity rule also follow a unimodal distribution (Supporting Fig. 8 **A,B**), distributions of I rates are unimodal and skewed to the right, and covariances and correlations are centered at zero in the balanced, asynchronous state (Supporting Fig. 8 **C–F**). However, the E rates are concentrated at the target rate (Supporting Fig. 8 **D** – inset), in disagreement with experimental findings [68]. In short, all three STDP rules show realistic distributions of weights, rates, and covariances, except for the iSTDP's distribution of E rates.

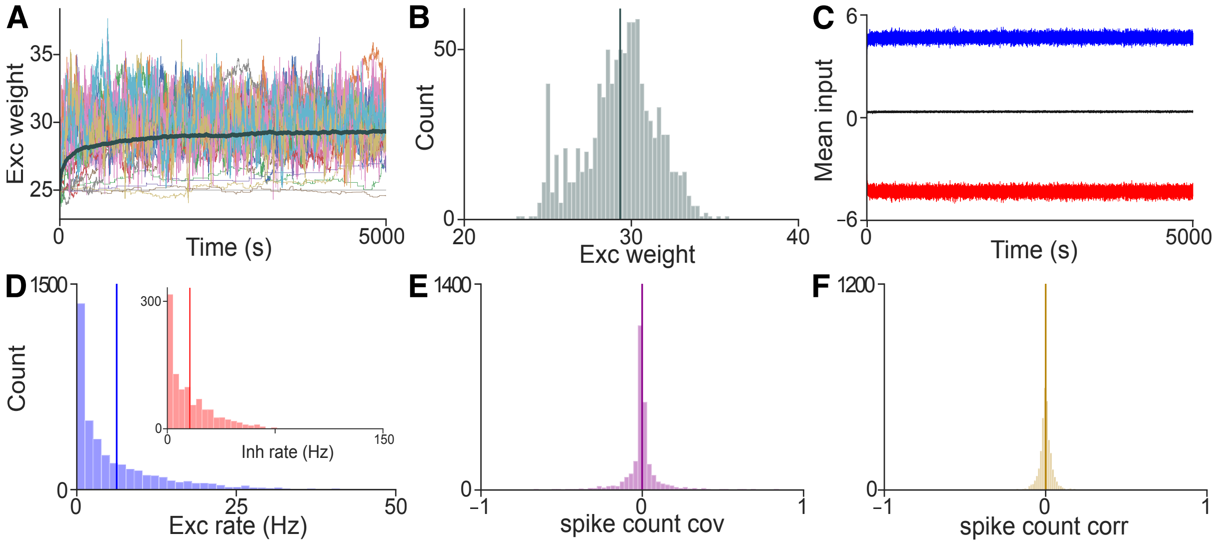

Supporting Figure 6: **Statistics of weights and activity under weight–dependent Hebbian STDP. A:** Evolution of individual synaptic weights and their mean (black). **B:** EE synaptic weights follow a unimodal distribution at steady state. **C:** Evolution of synaptic currents showing that balance is achieved and maintained. Color scheme as in Fig. 1 in text: blue denotes mean excitatory input, and red the mean inhibitory input. The total mean input is shown in black. **D:** Steady state distribution of excitatory and inhibitory (inset) firing rates. In equilibrium most neurons fire at low rates, and a few neurons fire at high rates. **E:** Distribution of spike count covariances. The mean is close to zero indicating an asynchronous state. **F:** Same as **E:**, but for spike count correlations, again showing a mean that is close to 0.

Fig. 2 A, D: It seems that these plots can be used to estimate the characteristic time it takes the synaptic weights to reach steady state values. I think the authors should expand the discussion of these results (l. 232-241). What is the relationship between the slope of dJ/dt *and* τ_{STDP}*? in other words, how many plasticity time constants does the synaptic weight transient last?*

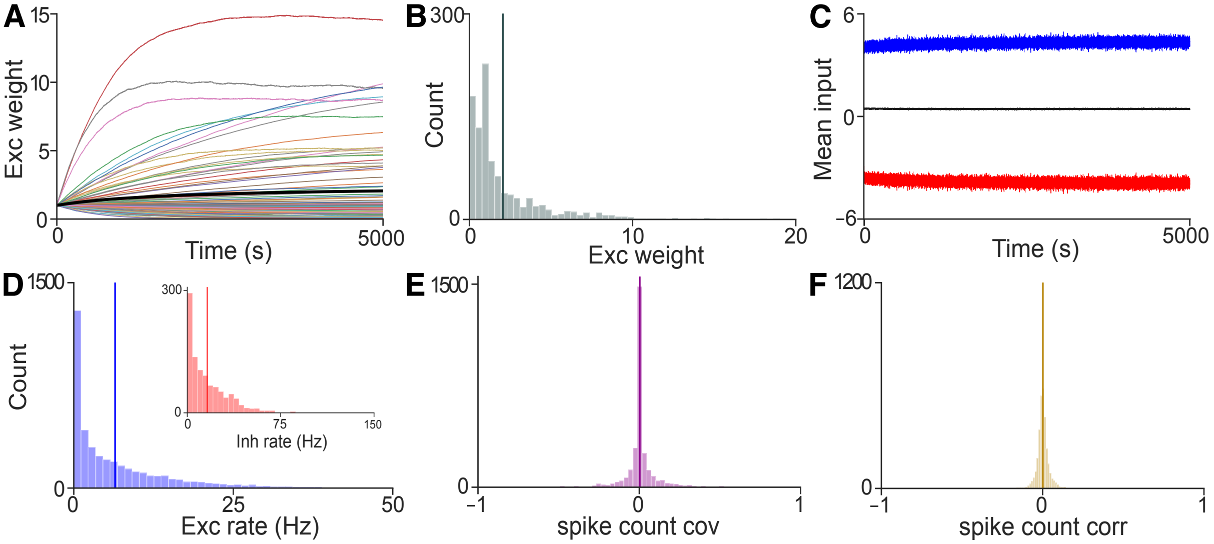

Supporting Figure 7: **Statistics and dynamics of balanced networks undergoing Kohonen's STDP rule.** **A:** Evolution of mean synaptic weight subject to Kohonen's STDP rule with mean synaptic weight in black. **B:** The distribution of EE synaptic weights at steady state is unimodal. **C:** Evolution of synaptic currents in the network. Balance is achieved and maintained. **D:** Equilibrium distribution of excitatory and inhibitory (inset) firing rates. Most neurons fire at low rates, and a few neurons fire at high rates. **E:** Distribution of spike count covariances shows that the mean is close to zero (asynchronous state). **F:** Same as **E:**, but for spike count correlations. The mean is close to zero, suggesting that the network is in an asynchronous state. Color scheme as in Supporting Fig. 6.

We believe there might be some confusion here between τ_{STDP} and $1/\eta_{ab}$. On one hand, τ_{STDP} is the time constant of eligibility traces, which determines the time window over which the eligibility traces, $x_j^a(t)$, are affected by past spikes. It does not substantially affect the slope of dJ_{ab}/dt. On the other hand, η_{ab} is the learning rate of the synaptic weights, and hence $1/\eta_{ab}$ is the time constant of STDP. The learning rules have the form $dJ_{ab}/dt = \eta_{ab}\cdots$. It then follows that the slope of dJ_{ab}/dt is proportional to $1/\eta_{ab}$.

There is a very strong slowing down as a function of input correlations (panel D). Does this mean that if rates are small, we should expect synaptic weights to always be away from steady state for correlated inputs? If not, can an experimental prediction be extracted from this or will the transient timescale always be swamped by firing rates?

Fig. 2 **D** shows the rate contribution to the time derivative of the synaptic weights ('Rate term' line) and the contribution of the correlation–dependent term to dJ_{ab}/dt at three different values of input correlations ('Corr' lines). For each $c_{\mathrm{x}} = 0.1, 0.5, 1$, the derivative of the weights is given the sum of the rate contribution (Fig. 2 **D** – blue line) and one correlation contribution (Fig. 2 **D** – orange, green, or red line). Correlations may shift the fixed point, modulate the speed of convergence, or both. This plot illustrates that

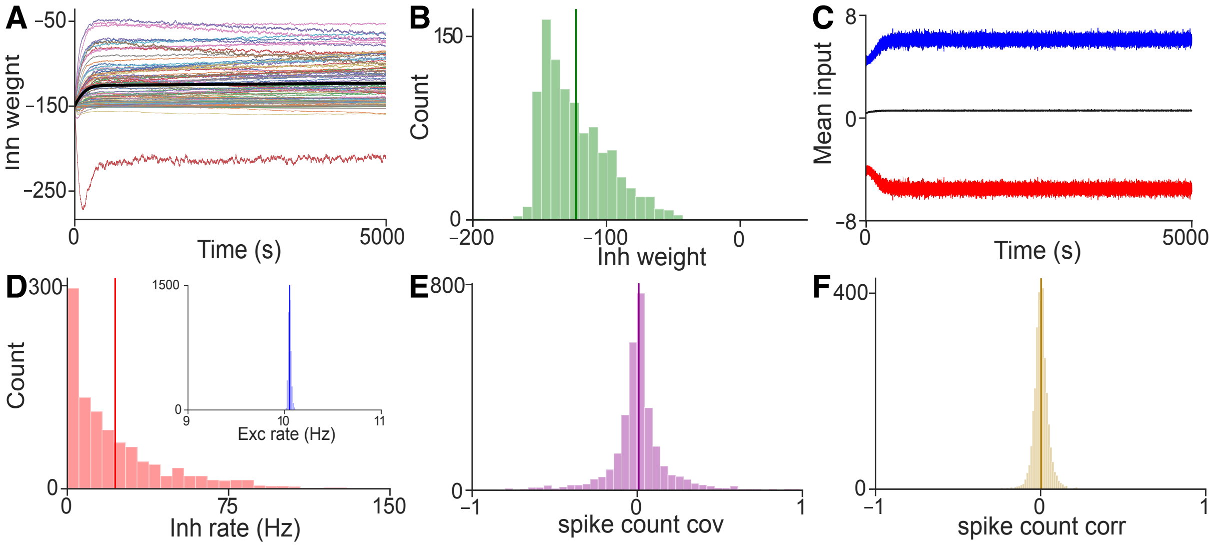

Supporting Figure 8: **Statistics and dynamics of balanced networks experiencing inhibitory STDP. A:** Evolution of *EI* synaptic weights subject to iSTDP. Mean synaptic weight in black. **B:** Distribution of *EI* synaptic weights at steady state. **C:** Evolution of synaptic currents in the network. Balance is achieved and maintained. **D:** Distribution of excitatory (inset) and inhibitory firing rates at equilibrium. Most inhibitory neurons fire at low rates, and a few neurons fire at high rates. All excitatory neurons fire at rates close to the 10Hz target rate. **E:** Distribution of spike count covariances with mean near zero (asynchronous state). **F:** Same as **E:**, but for spike count correlations. The mean is close to zero, suggesting that the network is in an asynchronous state. Color scheme as in Supporting Fig. 7.

the rate contribution typically dominates the correlation term (even at the largest value of input correlations $c_x = 1$), whether it be in determining the fixed point or modulating speed of convergence.

Even when rates are small (low values of j_{ee} in Fig. 2 **D**), we see that the rate term is still orders of magnitude larger than the correlation term. Thus our theory predicts that, generally, changes in synaptic weights in plasticity–inducing protocols (*e.g.*, optogenetic stimulation) will be mainly driven by modulation in firing rates, rather than in correlations.

We have modified text to more clearly present these findings.

How large is the impact of correlations in plastic balanced networks more generally? To address this question, we assumed that only pairwise interactions affect *EE* synapses, as first order interactions depend only on rates after averaging. We thus set $B_{\alpha,\beta} \equiv 1$, and all other coefficients to zero in Eq. (10). While the network does not reach a stable state under this arbitrary plasticity rule, it allows us to estimate the contribution of rates and covariances to the evolution of synaptic weights. Here $B_{\alpha,\beta}$ can have any nonzero value, since it

> scales both the rate and covariance terms. Under these conditions, our theory predicts that the rate term is at least an order of magnitude larger than the correlation term (even when rates themselves are small, *i.e.*, when j_{ee} is small), and so correlations only have a low impact on the dynamics of synaptic weights (Fig. 2 **D**). Therefore, our theory predicts that, in general, changes in synaptic weights will largely be driven by changes in firing rate patterns, rather than changes in pairwise correlations.

Fig. 2 C: is red E or I?

We have clarified this now in the legend of Figure 1. Unless otherwise stated, the color scheme remains the same throughout the paper.

> Unless otherwise stated, colors carry the same meaning in all figures.

Fig. 3 A: Is it possible to add a line to this panel showing the actual average synaptic weight in simulations in a way that takes into account the initial conditions? I would expect such a line to be close to 0 for a range of j values, perhaps making the result in panel B easier to understand and less confusing after looking at panel A.

Thank you for the suggestion. Yes, we have now included this in Fig. 3. See also our previous answer to the comment about convergence rates.

Fig. 3: increase font size of legend and inset figure labels.

Figure 3 has been modified with appropriate fontsizes.

257: The authors should cite Vegue Roxin 2019 here. They did take some steps in extending the MFT to cases with heterogeneous connectivity which leads to changes in the firing rate distribution (which are predictable given the weights).

We have now added the reference and the following text:

> The failure is due to correlations between weights and pre–synaptic rates which are typically ignored [13, 14, 23, 46–48], but can cause the mean–field description of network dynamics to become inaccurate. This is similar to the breakdown of balanced state theory in the presence of correlations between in– and out–degrees discussed by *Vegué and Roxin*, 2019 [69].

304: From a theoretical point of view, it is nice that introduction of II plasticity (of the same form as EI) eliminates the heterogeneity of inhibitory firing rates. However,

experimental data (e.g. Xue et al 2014, Chiu ,... Higley 2018) suggests that different inhibitory subtypes are subject to different inhibitory plasticity rules. I think it's fine to include this "trick" in a theoretical paper but I would at least mention the fact that inhibitory plasticity rules themselves are heterogeneous so in a real network we should not expect inhibitory firing rates to become similar due to homogeneity of the inhibitory plasticity rule.

This is an excellent point. Note that while one should not expect inhibitory firing rates to become uniform across subtypes, our mean-field approach can be extended to account for multiple subtypes, in which case we would only need (approximately) uniform firing rates within each subtype, or at least uncorrelated weights and rates within each subtype. But even that is probably not biologically very realistic. We have added the following text to the manuscript:

> This model of inhibitory plasticity is likely a large oversimplification. Synapses of different interneuron subtypes are likely subject to different plasticity rules operating on different timescales [17, 70], and would therefore not lead to uniform inhibitory firing rates. The mean-field theory we presented here can be extended to account for multiple inhibitory subtypes with different plasticity rules.

331: missing citation

Thank your pointing that out, we have now added the missing citation.

> Although classical mean–field theory produced singular solutions, Ebsch et al. showed that the theory can be extended, and that a non–classical balanced state is realized: Balance at the level of population averages (E and I) is maintained, while balance at the level of the three subpopulations is broken [52].

416: not sure that incapacity is a word. Consider using inability.

We agree and have modified the text now.

> Partial stimulation of a population of E neurons has been shown to break balance due to the inability of the network in cancelling inputs when weights are static [52].

416: typo and missing ref. "I Ebsch ..."

Thank you for catching this. We have now added the citation and fixed the typo.

> Ebsch et al. showed how classical balanced network theory can be modified to account for effects of input perturbations that break the classical balanced state [52]. Vogels et al. [41] (in addition to subsequent studies [35, 42, 71–74]) showed empirically using simulations that inhibitory iSTDP can restore balance. We here provide a theoretical framework that describes the evolution of rates and weights before, during, and after a perturbation that breaks balance.

426: The paragraph citing Ocker [57] should also cite Ravid-Tannenbaum Burak 2017

Thank you. We have now included the missing reference.

> Ocker et al. also studied the dynamics of mean synaptic weights in recurrent networks and derived equations for mean synaptic weights in terms of rates and covariances [44, 75].

Appendix 1, Generating correlated spike trains. I was not aware of this method. Its explanation is clear, but I lack intuition whether/why it is favorable over an alternative which seems simpler:

1. *Generating a Gaussian process with the exact desired correlations/covariances*
2. *Computing the Gaussian CDF of the random number for each neuron at each time.*
3. *Computing the inverse Poisson CDF of the result. (see a more complete description of the method in Macke Berens ... Bethge 2009)*

The method described by the reviewer is very powerful, allowing one to specify pairwise covariances for the generated spike trains. While this method is computationally feasible for hundreds of spike trains (as demonstrated by Macke, et al. [76]), it becomes computationally intractable at the numbers required for our study. For example, the simulation in Figure 3 required the generation of 10^3 spike trains over 3×10^7 time bins. The covariance matrix for the Gaussian process would contain 10^6 elements, so evaluation of the Gaussian CDF would be computationally expensive.

The method we used is much simpler, but more constrained. It produces spike trains with homogeneous pairwise correlations (all pairs have the same covariance), but it is

much more computationally efficient for generating a large number of spike trains. For example, the generation of spike trains for Figure 3 only took 24 seconds.

Appendix 3, I think the authors should expand Appendix 3 to address my major concern 1: Are there minimal modifications of the classical EE STDP rule which imply a non-trivial solution to the fixed point equation (Eq. 2 in Appendix 3)?

We have addressed this in the previous comments. Please see our answers to major concern 1, as well as extensions of S1 Appendix, including the added figures.

Referee B

The authors developed a self-consistent theory of rates, covariance and synaptic weights in balanced recurrent network that receives correlated external input. The theory can help to understand coexistence of correlated activity and dynamic excitatory-inhibitory balance. Furthermore, they found a plasticity rule for inhibitory-to-excitatory connections which have interesting property of dependence on initial condition. Finally, they showed that their theory could be applied to "out of balance" scenario of optogenetic input. Although, these are interesting and important claims we have some reservation about the amount of proof they provide for such a general claims and consequently about biological relevance of the study.

First claim is that authors developed a general theory of plasticity in balanced network which can address network dynamics with different plasticity rules and describe weight evolution with correlated activity. We have some concerns about generality, novelty and predictive power of the theory. The theory presented is far from being a general theory of plasticity in balance networks. The claim hold and have be demonstrated by authors fairly well only in the case of tightly balanced large neural network (with asymptotic 1/sqrt(N) scaling) for the class of pairwise STDP rules (ignoring other plasticity types i.e. bcm, metaplasticity, intrinsic plasticity and structural plasticity). This STDP class authors chose is one with multiplicative dependence on synaptic weights which naturally lead to "nice" fixed points and for which mean-field description works very well.

We agree with this point. We have rewritten the manuscript to clarify the limitations of the theory we develop. We believe that a similar theory can be developed for other types of balanced networks as long as a mean-field description holds, and timescales are separated. For learning rules that involve higher order interactions this will require extensions of the mean field theory.

> Higher order interactions are at the heart of triplet rules [59–61, 77], and other types of interactions may also be important, *e.g.* for calcium–based update rules [62, 63]. For simplicity we here focus on pairwise interactions between spikes and eligibility traces, and leave extensions to more complex rules for

future work.

We have also added the following text to the Discussion:

Our theory assumes a large network, $N \gg 1$, and weights that scale as $1/\sqrt{N}$ resulting in "tight balance". The theory is less accurate in smaller networks. However, our simulations show that the theory provides good approximations for biologically realistic network sizes and weights.

The main theoretical contribution of the paper is procedure to put together and solve self-consistently equations for rate, covariance and weights. This theory seems very computational efficient and simple, but it is not the only one which can threat this problem. Equations 1 and 2 for rate and covariance with tight balance condition and large networks are already derived earlier (partly by the same authors Baker et al 2019 "Correlated states in balanced neuronal networks"), and there is even a statistical field theory developments in this area e.g. Ocker et al 2016 paper "Linking structure and activity in nonlinear spiking networks". The equation for individual weight evolution with input correlation were derived already by Gutig et al 2003 in paper "Learning Input Correlations through Nonlinear Temporally Asymmetric Hebbian Plasticity". The self-consistent weight evolution was done by Ocker et al 2015 in the paper "Self-Organization of Microcircuits in Networks of Spiking Neurons with Plastic Synapses", for the case of pairwise STDP rule which allows for weight heterogeneity and structure in connectivity (they chose a rule which is very sensitive to correlation in inputs). There is also work with higher-order SPDP rule, i.e. Monangie et al 2020 paper "Autonomous emergence of connectivity assemblies via spike triplet interactions".

Thank you for the comment. We have rewritten our introduction to better place our work in the context of balanced networks and spike timing dependent plasticity. Please see the new introduction text at the beginning of the document. We have also included the missing citation to Gutig et al., 2003 and Montangie et al., 2020.

To my best of my knowledge this is the first theoretical study of effect of external input correlation on tightly-balance balanced plastic networks. Similar theoretical attempts have been done before for the cases of external and self–generated correlations, e.g. Gilson et al 2009 "Emergence of network structure due to spike-timing-dependent plasticity in recurrent neuronal networks. I. Input selectivity–strengthening correlated input pathways" and Gilson 2010 "STDP in recurrent neuronal networks". Above studies did not have condition for tight balance and this work is based on this assumption. It is not clear if prescribing a balance a priory could bring clarity to the issue, because temporal dynamics of correlation can play a big role in unstable dynamical states, e.g. trough large Fano factor.

This is a good point. Our theory relies on the assumption that the system is in quasi–equilibrium. It is likely that in some situations weight changes are driven by events that place the network out of equilibrium. On the other hand, we do point to evidence that, for instance, inhibitory plasticity may act over longer timescales to keep the network stable. There is also considerable evidence that many cortical networks are in a balanced state. As we stated above, we believe that our theory provides a basis for describing plastic balanced networks when a separation of timescale holds. These are the same conditions that underlie much of previous theoretical work. We believe that this is the first time this approach has been extended to balanced networks, as the reviewer notes.

We now point to the limitations of the theory in the following text added to the Discussion:

> It is likely that some changes in the weights are driven by transient events that place the network out of equilibrium. If plasticity is mainly driven by such fast changes, the timescale separation central to our theory does not hold. As we have shown in S1 Appendix, the predictions of the theory can break down in such cases.
>
> One of the most important issues in understanding neural dynamics is establishing conditions under which the network remains active, yet stable as synaptic weights change. The theory we developed can help us address these questions, but it does have limitations. Since we used a mean-field approach, we can only capture first moments. While mean weight stability may not imply stable network dynamics (consider the case when weight variance diverges in Classical Hebbian STDP in S1 Appendix), instability in the mean weights does imply that the network is also unstable.

Second claim is that that correlations in the input mildly, but significantly affect the evolution of synaptic weights. This claim is well demonstrated, but it is a hardly surprising, given the type of the multiplicative pairwise rule authors use.

We agree that in retrospect this may not be surprising, and it is a general consequence of these learning rules. We now note that Ocker et al., 2015, and Graupner et al., 2016 have made similar observations. This conclusion is somewhat restricted to the types of rules that we used. For instance, as also shown by Ocket, et al., if we reduce the weight dependent terms by choosing a nearly anti-symmetric STDP function, correlations can drive plasticity [44]. Our theory can be applied to this case. We can also extend the main ideas to other types of plasticity rules as long as separation of timescales holds, although this may require describing the higher order statistics of the network. We now make a note of both of these points.

> Higher order interactions are at the heart of triplet rules [59–61,77], and other types of interactions may also be important, *e.g.* for calcium–based update

rules [62, 63]. For simplicity we here focus on pairwise interactions between spikes and eligibility traces, and leave extensions to more complex rules for future work.

We have also commented about this in the Discussion:

Ocker et al. also studied the dynamics of mean synaptic weights in recurrent networks and derived equations for mean synaptic weights in terms of rates and covariances [44, 75]. They also found that the effect of correlations in synaptic weight dynamics is mild, however, their approach relied on linear response theory to obtain expressions of spike train covariances. Such a description is valid when neurons are driven by strong white noise in addition to weaker synaptic input from the network. Using networks of two neurons with varying firing patterns, Graupner et al. also found that changes in synaptic weights were dominated by firing rates, with correlations playing a secondary role [51].

Third claim is that their inhibitory-to-excitatory plasticity induce correlations between activity and synaptic weights, such that final weight depends on initial synaptic weight. This is certainly interesting result, and especially because it could explain one role of inhibitory-to-inhibitory plasticity. That being said the rule used is very peculiar. The RHS of the ODE is scaled with initial position and authors did not fully clarify what is the consequence of this choice? What is biological justification? It is very confusing for reader that initial position is a parameter of an ODE. We would be interested to see a comparison of this rule to the original Vogels at al 2011 "Inhibitory plasticity balances excitation and inhibition in sensory pathways and memory networks" to properly understand the scaling choice. More importantly temporal evolution of the weights is not shown in this case.

We understand why this can be confusing, especially since we show that weights in iSTDP depend on the initial condition later on. The only purpose of this normalization constant was to match the dimensions on both sides of the equation. The same could have been accomplished by absorbing the constant in the learning rate, η_{ab}.

When we implemented the rule we used a single constant in all simulations, rather than an initial condition. Thus the equation we provided was in error and was not consistent with the simulations we presented, and the actual simulations are closer to what the reviewers suggest above. This holdover from a previous analysis has now been corrected in the text.

The normalization does not depend critically on the specific value of the constant, but it is required to be of the same order (and sign) as synaptic weights J_{jk}^{ei}, that is $\mathcal{O}(1/\sqrt{N})$ and negative. To clarify this point have now renamed the normalizing constant J_{norm}, and addressed this in "Results: Balanced Network with Inhibitory Plasticity".

To illustrate this, we consider a balanced network subject to homeostatic plasticity [41]. This type of plasticity has been shown to stabilize the asynchronous balanced state and conjectured to play a role in the maintenance of memories [35, 41, 72]. Following [41] we assume that EI weights evolve according to:

$$\frac{dJ^{\text{ei}}_{jk}}{dt} = -\eta_{\text{ei}} \frac{J^{\text{ei}}_{jk}}{J_{\text{norm}}} \left[(x^{\text{e}}_j - \alpha_{\text{e}}) S^{\text{i}}_k + x^{\text{i}}_k S^{\text{e}}_j \right] \tag{4}$$

where α_{e} is a constant that determines the target firing rates of E cells and $J_{\text{norm}} \sim \mathcal{O}(1/\sqrt{N})$ is a normalization constant. Note that $J_{\text{norm}} < 0$ so the fraction in Eq. (4) is positive assuming $J^{\text{ei}}_{jk} < 0$. In a departure from the rule originally proposed by Vogels et al. [41], we chose to multiply the time derivative by the current weight value. This modification creates an unstable fixed point at zero, prevents EI weights from changing signs, and keeps the analysis mathematically tractable (See S1 Appendix for details). An alternative way to prevent weights from changing sign would be to place a hard bound at zero, but this would create a discontinuity in the vector field of J_{ei}, complicating the analysis.

Final claim is that this framework could be used to treat case of optogenetic input to subpopulation. We think that this claim is shown well, but it is the question if the study brings more clarity to the field than original Vogels 2011 study.

We agree this may have not been very clear. Our main result here is not that iSTDP can help maintain balance. As the reviewer points out, this was already shown by Vogels et al., 2011. Rather we provide a theory that describes the evolution of weights and rates before, during, and after stimulation. While Vogels et al. demonstrated and explained how iSTDP can restore tight balance, as far as we know, ours is the first analytical description of the evolution of the actual values of mean firing rates and synaptic weights during and after a balance–breaking perturbation. In the manuscript, this serves as a demonstrative example of how our theoretical framework can be applied, using an example that arises naturally in previous work.

To explain this point more clearly we have added the following to the Discussion:

Ebsch et al. showed how classical balanced network theory can be modified to account for effects of input perturbations that break the classical balanced state [52]. Vogels et al. [41] (in addition to subsequent studies [35, 42, 71–74]) showed empirically using simulations that iSTDP can restore balance. We here provide a theoretical framework that describes the evolution of rates and weights before, during, and after a perturbation that breaks balance.

Referee C

The manuscript addresses the dynamics of plasticity under STDP in randomly connected neural networks in the balanced regime. The manuscript provides some analytical insights into this topic, backed up by numerical simulations. The topic is important, and results seem substantial and interesting. Nevertheless, there are some significant weaknesses in the analysis and presentation, which I recommend to address before the manuscript is considered again for publication.

Since the theory is derived at the level of mean population activities, there is a hidden assumption that synaptic weights evolve uniformly. Is this true empirically in the simulations? How variable are the weights following plasticity?

Even though mean–field approximations are accurate under uniformity, they can also be accurate when weights and rates are not uniform. Our theory only requires that $\langle J_{jk}^{ab} r_k^b \rangle_{j,k} \approx \langle J_{jk}^{ab} \rangle_{j,k} \langle r_k^b \rangle_k$ where $\langle \cdot \rangle_{j,k}$ is the average over indices j and k. We provide an example of when and how this assumption breaks, and we describe a semi–analytic approach to restore the mean–field theory (Fig. 3).

We have now included supplementary figures that show the uniform evolution of randomly sampled individual weights in Kohonen's, weight–dependent Hebbian, and inhibitory STDP rules in (Supporting Figs. 6 **A**,7 **A**,8 **A**). A longer discussion of this new material is provided in the response to comments by Reviewer A.

Related to this point, there is also an implicit assumption that weights evolve independently of other quantities. One example is the correlation between presynaptic firing rate and the synaptic weight, which is discussed in the section on inhibitory plasticity. But there are other possible correlations that could develop between synaptic strength and, for example: the in-degree and out-degree of involved neurons; under some rules competition between synapses belonging to the same neuron could also break some of the assumptions of the mean field theory.

We agree with this comment. In all of our simulations, the in-degree and out-degree are almost equal, because $p_{ab} \equiv 0.1$ for all $a, b = e, i, x$ and N was large. Different extensions of the theory would have to account for other types correlations between different weights, degrees, and/or activity. We have now cited the work by *Vegué and Roxin* 2019, and commented on it in the Discussion:

> In plastic networks, correlations between weights and other features such as in–degrees, or out–degrees can emerge [69]. We have shown how the theory can capture the case in which synaptic weights and pre–synaptic rates are correlated. While we were not able to find analytical expressions for these correlations, we showed that a second–order correction is sufficient to explain the observed dynamics. Eventually, the mean–field theory would need to be extended to account for higher order network motifs and their potential correlations with synaptic weights and firing rates. This might be possible by extending our approach, but we leave these extensions for future work.

A full theory of the plasticity dynamics should address these questions, but I accept that there is value in deriving the predictions of a mean field theory that ignores such correlations, while comparing the predictions with numerical results. The presentation should acknowledge these limitations more explicitly. The appearance of a discussion of correlations between firing rates and synapses only in the section on inhibitory plasticity is unsatisfactory.

We agree with this point and have added it to the Discussion.

> In plastic networks, correlations between weights and other features such as in–degrees, or out–degrees can emerge [69]. We have shown that the theory applies to the case in which synaptic weights and pre–synaptic rates are correlated. While we were not able to find analytical expressions for these correlations, we showed that a second–order correction is sufficient to explain the observed dynamics. Eventually, the mean–field theory would need to be extended to account for higher order network motifs and their potential correlations with synaptic weights and firing rates.

We have also added the following text to the Methods:

> Higher order interactions are at the heart of triplet rules [59–61, 77], and other types of interactions may also be important, *e.g.* for calcium–based update rules [62, 63]. For simplicity we here focus on pairwise interactions between spikes and eligibility traces, and leave extensions to more complex rules for future work.

Is there a way to understand why correlations did not play an important role in earlier sections?

This is an important question, and we have added a paragraph to the Discussion to address this more clearly.

> A natural question that arises is why do correlations between weights and pre–synaptic rates only seem to play a role in iSTDP? In the examples of excitatory STDP we analyzed (Kohonen's rule and weight–dependent Hebbian rule), weights at equilibrium are determined by other parameters (Weight–dependent Hebbian rule) or rates (Kohonen's rule). Therefore weights are updated until those steady state values are achieved, yielding values independent of initial conditions. On the other hand, in the case of the inhibitory plasticity rule, inhibitory weights at equilibrium are determined by the firing rates alone. Since the firing rate vectors are lower–dimensional than the weight matrices, the equilibrium solution does not fully determine the weight matrices. This is shown in Fig 3 **C** inset, where different distributions of weights

can result in the same equilibrium firing rate when weights and pre–synaptic rates become correlated.

While the theory is derived for dynamics of the synaptic weights, the numerical analysis does not demonstrate that the dynamics indeed follow equation 6. Instead, simulations are used only to demonstrate agreement with the predictions on the steady state. Do the dynamics follow the prediction?

We agree. The system of equations can be solved iteratively over time as well, provided the timescales are separated. We have now included this in S1 Appendix: "Transient dynamics of synaptic weights".

Transient dynamics of synaptic weights. In much of this work we focused on steady state behavior. However, the system of equations (Eqs. (14,21,71)) relating rates, covariances, and weights can also be solved iteratively over time, provided the timescales are well separated. Here, we show three examples: weight–dependent Hebbian STDP, Kohonen's rule, and iSTDP. In all three cases, the observed trajectory of synaptic weights closely follows the theoretical predictions (Supporting Figs. 3 **A–C**).

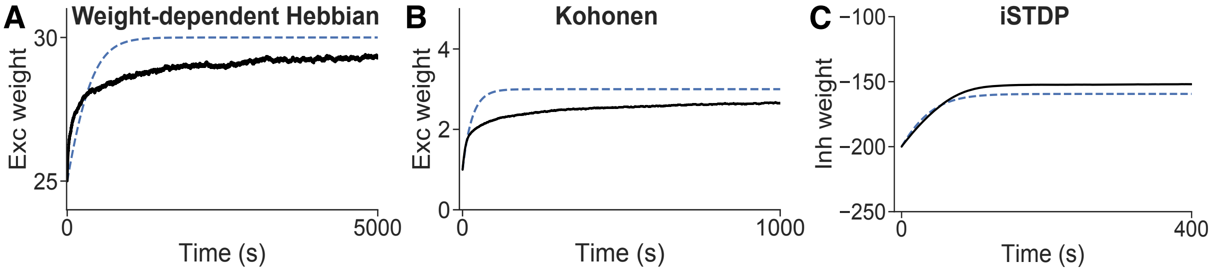

Supporting Figure 3: **Transient dynamics of synaptic weights. A:** Mean synaptic weight in a network subject to "weight–dependent" Hebbian STDP. **B:** Same as **A**, but for Kohonen STDP. **C:** Same as **A**, but for inhibitory STDP. In all panels, dashed lines represent the theory (Eqs. (14,21,68)); solid lines represent numerical simulations.

I would also like to see some numerical support for statements that the network remains in an asynchronous state following plasticity.

We agree that it is important to demonstrate this. We have added a new section to S1 Appendix, where we analyze the distribution of synaptic weights, rates, and covariances. We also show that the network is balanced throughout. Supporting Figs. 6–8 **E,F** show that mean covariances are very weak, *i.e.*, the network remains in the asynchronous state.

The figures are included earlier in the letter, and we repeat the new text here for convenience:

> **Statistics and dynamics of balanced networks under pairwise STDP rules.** Our theoretical results describe the evolution of averages (See Methods in main text). However, it is also important to understand changes in higher order statistics of individual, and collective activity under plasticity. Although we do not go beyond a mean–field theory, we provide the results of corresponding numerical simulations in three example balanced networks where: (1) excitatory weights were subject to weight–dependent Hebbian STDP; (2) *EE* weights changed according to Kohonen's rule; and (3) inhibitory weights changed according to an iSTDP rule (See Table 1 in main text for equations describing each rule).
>
> Under weight–dependent Hebbian STDP individual synaptic weights converge to a unimodal distribution. This is in agreement with experimental results [68] (Supporting Fig. 6 **A,B**). Balance is preserved throughout the simulation (Supporting Fig. 6 **C**). Distributions of rates are unimodal and skewed to the right (Supporting Fig. 6 **D** and inset), also agreeing with experimental findings [68]. Spike count covariances and correlations are distributed around zero in the asynchronous state (Supporting Fig. 6 **E,F**).
>
> *EE* weights under Kohonen's rule also converge to a unimodal distribution (Supporting Fig. 7 **A,B**). Again, the network is stable and balance is preserved (Supporting Fig. 7 **C**). The distributions of rates is again unimodal and skewed to the right (Supporting Fig. 7 **D**), and covariances and correlations are close to zero (Supporting Fig. 7 **E,F**). Lastly, *EI* weights subject to an inhibitory plasticity rule also follow a unimodal distribution (Supporting Fig. 8 **A,B**), distributions of *I* rates are unimodal and skewed to the right, and covariances and correlations are centered at zero in the balanced, asynchronous state (Supporting Fig. 8 **C–F**). However, the *E* rates are concentrated at the target rate (Supporting Fig. 8 **D** – inset), in disagreement with experimental findings [68]. In short, all three STDP rules show realistic distributions of weights, rates, and covariances, except for the iSTDP's distribution of *E* rates.

Lines 359-365: What happens under the perturbation without plasticity? How does this depend on parameters? How can we see that the network is in a balanced state following plasticity (but possibly not before plasticity)?

After external input onset, the network initially exits the balanced state when some E neurons are silenced due to excess average inhibitory input (Fig. 4 **D**). However, while targeted cells still receive optogenetic input, inhibitory weights adjust to restore detailed balance in the network. The network will remain at or return to the balanced state so

long as parameters are chosen such that W is non–singular. Thus a prolonged input will drive the network to a new balanced state, while a transient input may drive the network out of balance for a short period.

We now clarify this point in the following:

> We simulated a network of $N = 10^4$ neurons in an asynchronous state with $c_{x_1} = c_{x_2} = 0$. A subpopulation of 4000 E cells receives transient, correlated input. Solving Eqs. (27,28) predicts that inhibitory plasticity will alter EI synaptic weights so that the firing rates of both the E_{expr} and the $E_{\text{non-expr}}$ approach the target firing rate before, during, and after stimulation. Once the network reaches steady state the mean inputs to each subpopulation cancel. Thus changes in EI weights restore balance at the level of individual subpopulations or "detailed balance," consistent with previous studies [41, 72]. Simulations confirm these predictions (Fig. 4 **B–D**).
>
> When the input is removed, the inhibitory weights onto cells in the E_{expr} subpopulation converge to their pre–stimulus values, returning E_{expr} rates to the target value, and reestablishing balance (Fig. 4 **B–D**). Correlations remain low ($\mathcal{O} \sim 10^{-4}$) before, during, and after stimulation (Fig. 4 **E,F**), suggesting that at equilibrium the network is in the asynchronous state. Our theory thus describes how homeostatic inhibitory STDP increases the stability and robustness of balanced networks to perturbations by balancing inputs at a level of individual cells, maintaining balance in regimes in which non–plastic networks cannot maintain balance. We presented an example in which only one subpopulation is stimulated. However, the theory can be extended to any number of subpopulations in asynchronous or correlated balanced networks receiving a variety of transient stimulus.

In comparisons between simulations and the theory, for how long were the simulations run? What was the criterion used to identify that the weights reached steady state?

We have added these details in the captions throughout the text. We applied the Kolmogorov–Smirnov 2–sample test to determine if synaptic weights had reached equilibrium. We added text clarifying this in "Appendix: Determining when synaptic weights reach equilibrium in simulations".

> **Determining when synaptic weights reach equilibrium in simulations.** To determine if synaptic weights had reached steady state we sampled 1000 synaptic weights from the whole population (of plastic weights) at times $t = 4000$sec and $t = 5000$sec (end of simulation). We then compared the two distributions using the Kolmogorov–Smirnov 2–sample test. We found that in the three networks simulated in Supporting Figs. 6–8, the distributions were not distinguishable, and hence had reached equilibrium by the end of the simulation (Supporting Fig. 4 **A–C**)

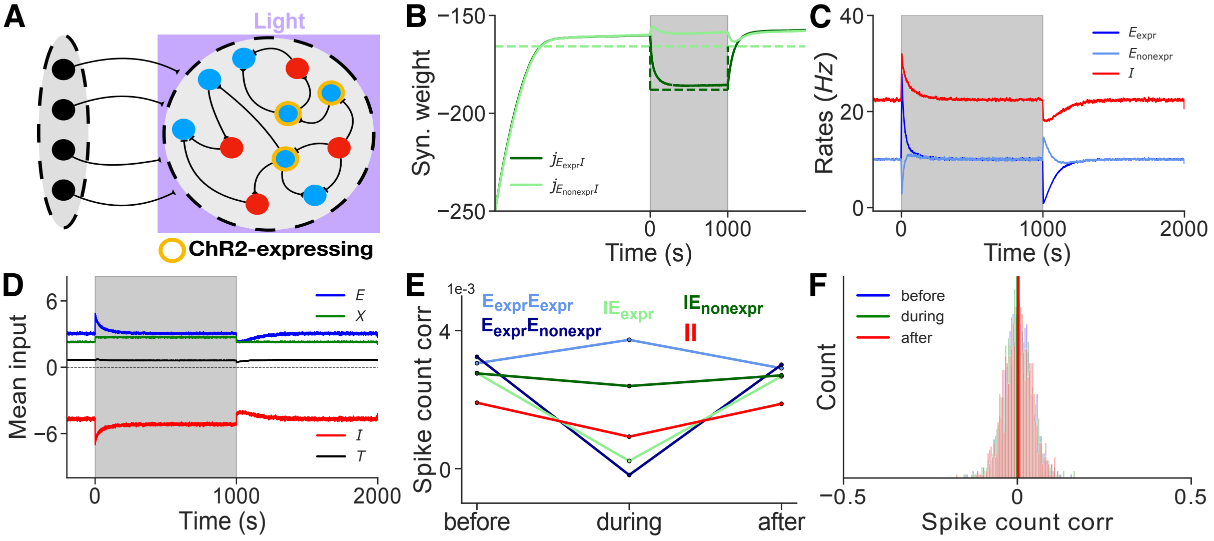

Figure 4: **General framework for STDP in balanced networks describes the dynamics of networks receiving optogenetic input.** **A:** A recurrent network of excitatory, E, and inhibitory, I, neurons is driven by an external feedforward layer X_1 of uncorrelated Poisson neurons. Neurons that express ChR2 are driven by optogenetic input, which is modeled as an extra layer of Poisson neurons denoted by X_2. **B:** Evolution of mean synaptic weights over the course of the experiment. **C:** Evolution of mean firing rates. Inhibitory STDP maintains E rates near the target, $\frac{\alpha_e}{2\tau_{\mathrm{STDP}}}$. **D:** Evolution of mean excitatory, external, inhibitory, and total currents. Balance is transiently disrupted at stimulus onset and offset, but it is quickly restored by iSTDP. **E:** Mean spike count correlations before, during, and after stimulation remain very weak for all pairs. **F:** The distribution of spike count correlations also remains nearly unchanged with weak mean correlations before, during, and after stimulation. Solid lines represent simulations, dashed lines are values obtained from theory (Eqs. (3,5,27,28)).

Line 6: it will be appropriate to cite also Shaham and Burak (2017) and Darshan et al (2017) in the context of spatially correlated activity in the balanced state.

The introduction has been rewritten to more appropriately place the work in the balanced network and synaptic plasticity literature and these references have now been added.

> Cortical neuronal activity is typically irregular, correlated, and can be dominated by a low dimensional component [1–6]. Cortical activity is also characterized by a balance between excitation and inhibition [1,7–12]. Such balance is now a widely thought to give rise to stable, irregular neural activity [1,7,12–18]. Early theoretical work has focused on irregular asynchronous dynamics, with large networks exhibiting vanishing correlations [13,19]. However, more recent extensions have shown how correlated dynamics can be generated both endogenously and exogenously, while preserving irregular single cell activity [20–28], showing the existence of both asynchronous and correlated states in balanced

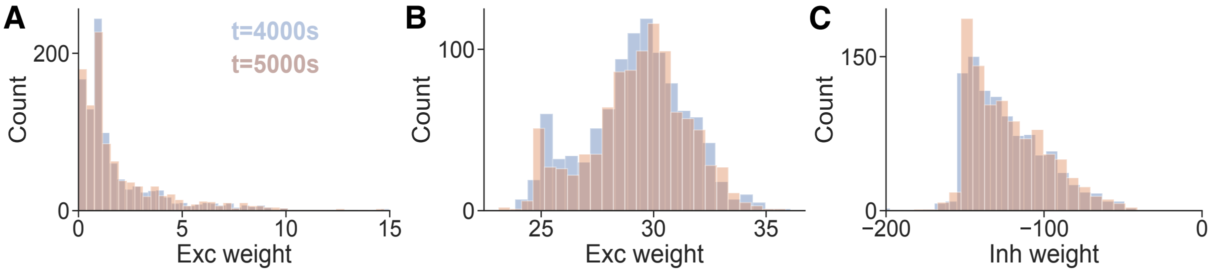

Supporting Figure 4: **Distributions of synaptic weights are stationary. A:** Distributions of EE synaptic weights for a balanced network with excitatory weights evolving according to Kohonen's rule at times $t = 4000$sec and $t = 5000$sec. The two distributions are not different according to the Kolmogorov–Smirnov 2–sample test ($D_n = 0.029$, p–value$= 0.8$). Hence, EE weights are at steady state. **B:** Same as **A**, but for a network with weights that follow weight–dependent Hebbian STDP showing excitatory weights are in equilibrium ($D_n = 0.045$, p–value$= 0.26$). **C:** Same as **A**, but for a network where EI weights are subject to iSTDP. Inhibitory weights are in equilibrium ($D_n = 0.024$, p–value$= 0.94$). All simulation parameters as in Supporting Figs. 6–8.

> networks.

In the discussion, it will be appropriate to cite Ravid-Tannenbaum and Burak (2016) in the context of the relationship between covariances and STDP dynamics.

Thank you. We have now included the missing reference.

> Ocker et al. also studied the dynamics of mean synaptic weights in recurrent networks and derived equations for mean synaptic weights in terms of rates and covariances [44, 75].

Lines 58-59: why is the assumption not essential? How does this agree with Ref. 34, which shows that heterogeneity in the connection probabilities can destroy the balanced state?

We meant that this assumption is not essential as long as the matrix W is non–singular. Balance can be maintained or destroyed depending on several factors, and heterogenous connectivity may not necessarily disrupt it (Pyle PRE, 2016). We have modified our statement to clarify this.

> For simplicity we assume that both the probabilities, p_{ab}, and weights, $j_{ab} \sim \mathcal{O}(1)$ are constant across pairs of subpopulations. This assumption is not essential as long as the balance condition remains valid. This is equivalent to having a non–singular mean–field connectivity matrix $\overline{W}$, which is defined next.

PRESENTATION

It is inconvenient that only some of the equations are numbered. This makes it difficult to refer to the unnumbered equations when discussion the manuscript.

We have now numbered all the equations for the readers' convenience.

Equation 2: please define Gamma precisely, not only in limits, and either derive this result or provide a clear path from the results of previous papers to this expression, possibly in an appendix.

We now point to detailed derivations of Γ already presented in Baker et al., 2019. A closed form for finite N is not tractable for spiking neural networks.

> The matrix Γ has the same structure as C and represents the covariance between external inputs (See Baker et al., 2019 Appendix A for a detailed derivation of this term [23]).

The role of Twin in equation 2 is not sufficiently clear. If I understood this correctly, Twin should be introduced already when defining the spike count covariance.

We agree with this comment and now define T_{win} earlier.

> We define the mean spike count covariance matrix as:
>
> $$C = \begin{bmatrix} C_{\text{ee}} & C_{\text{ei}} \\ C_{\text{ie}} & C_{\text{ii}} \end{bmatrix}$$
>
> where C_{ab} is the mean spike count covariance between neurons in populations $a = \text{e}, \text{i}$ and $b = \text{e}, \text{i}$ respectively, counted over time windows of size T_{win}. Throughout all simulations and theoretical predictions, we set $T_{\text{win}} = 250\text{ms}$, however the theory is flexible to other time window sizes.

Lines 73-74: the statement that the second term in Equation 2 accounts for intrinsically generated variability is a bit confusing since the elements in the diagonal are negative. It will be helpful to clarify this.

We realize how this was confusing. We now remind the reader that in the spike count covariance matrix the diagonal terms are not variances, but the mean covariances between spike trains in the same population. We have clarified this in the following added text:

In Eq. (5), F_a is the Fano factor of the spike counts averaged over neurons in populations $a = \mathrm{e}, \mathrm{i}$ over time windows of size T_{win}. The second term in Eq. (5) is $\mathcal{O}(1/N)$ and accounts for intrinsically generated covariability [23] within excitatory or inhibitory populations (note that this term does not refer to variances, but instead to mean covariances between spike trains in the same subpopulations).

The logic of how x and S combine to generate the STDP rule is mathematically simple, yet for many readers it will be difficult to understand the structure of the rule from the equations. It will be helpful to provide a graphical representation of the STDP rules in the different cases listed in Table 1.

We agree with this comment. We have now added Supporting Fig. 1 to show the STDP window for each rule presented in Table 1.

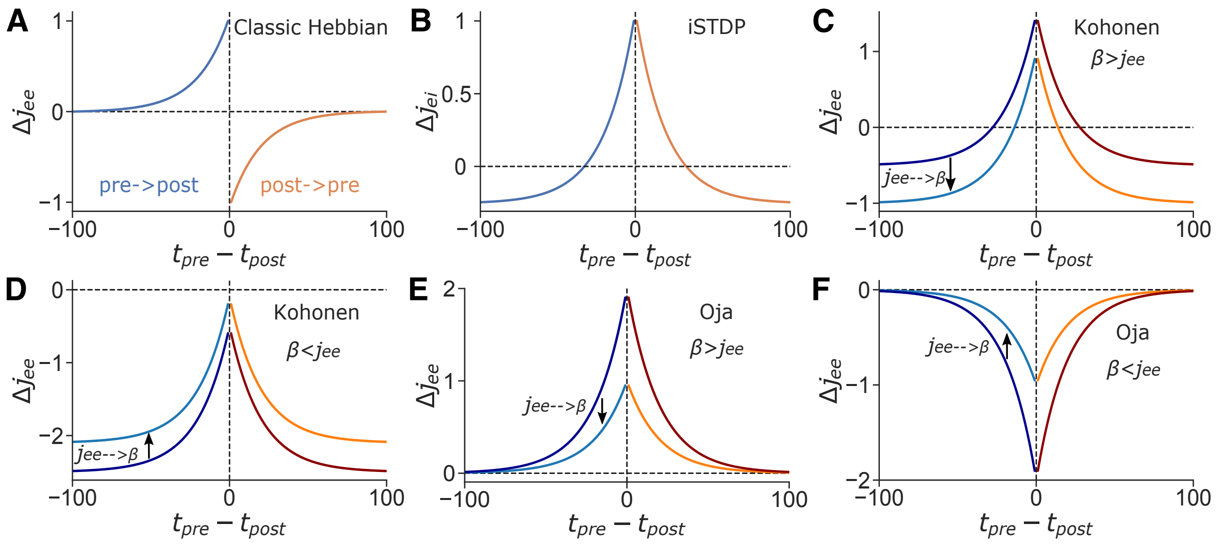

Supporting Figure 1: **STDP windows of different plasticity rules.** **A:** Change in synaptic weights as a function of the relative timing of pre– and post–synaptic spikes in Classical Hebbian STDP (same as weight–dependent Hebbian). **B:** Same as **A**, but for inhibitory STDP. **C:** Same as **A**, but for Kohonen's rule when weights are below parameter β. **D:** Same as **C**, but for the case when weights are above β. **E:** Same as **A**, but for Oja's rule when weights are below parameter β. **F:** Same as **E**, but for the case when weights are above β.

Lines 140-148: I didn't understand why root finding is involved in the numerical solution of the differential equations. Doesn't equation 6 simply provide the required update of J, based on the values of r anc C that are obtained from equations 1-2?

We realize this was not clear before and have addressed this now.

> This process can be repeated iteratively at each time step to obtain the evolution of firing rates, spike count covariances, and synaptic weights provided our assumption about the separation of timescales holds (See S1 Appendix: "Transient dynamics of synaptic weights" for more details).

Figure 3: "and the system predicts a stable fixed point". Should "system" be replaced by "theory"?

Yes, thank you for pointing that out.

> Fig 3: **A:** The rate of change of EI weights as a function of the weights themselves. The contributions of the covariance (blue) is considerably smaller than the contribution of the rate (red), and the theory predicts a stable fixed point.

Line 339: sentence ends prematurely.

Thank you for catching this.

> We show that inhibitory STDP, as described by Eq. (22), can restore balance in the inputs to the stimulated and unstimulated subpopulations.

The last equation in page 1 of SI1 includes parameters whose meaning is not defined.

Thank you for noticing that. We have now pointed to the specific table containing these parameters.

> See Supporting Table 1 for a description and values of the parameters of the voltage dynamics. In our simulations, this equation was integrated using Forward Euler's method with step size $dt = 0.1$ms.

In line 26 of SI1, units for dt are missing.

Thank you for pointing that out. We now specify units: $dt = 0.1$ms

In line 53 of SI1, in the equation $rm = r_x/cx$, should cx be c_x? (where _ stands for subscript).

Yes, thank you. We have now fixed this typo:

To generate N_{x} processes, all having firing rate r_{x} and pairwise correlation c_{x}, we first generate a "mother" process with rate $r_m = r_{\mathrm{x}}/c_{\mathrm{x}}$.

Lines 53-56: It is not easy to understand from the description how the daughter process spikes are obtained. I suggest to clarify that each daughter process is obtained by first taking all the spikes of the mother process, followed by a deletion of spikes with probability $1 - c_x$, and to mention explicitly that deletion is performed independently in each one of the daughter processes.

We agree with this comment, and have made this point more clear in the text.

To generate N_{x} processes, all having firing rate r_{x} and pairwise correlation c_{x}, we first generate a "mother" process with rate $r_m = r_{\mathrm{x}}/c_{\mathrm{x}}$. Then, to generate each of the N_{x} "daughter" processes, we first take all the spikes in the mother process and then delete each spike independently with probability $1 - c_{\mathrm{x}}$. In other words, each spike time from the mother process is included in each daughter process independently with probability c_{x}. The rates of the daughter processes are $r_m c_{\mathrm{x}} = r_{\mathrm{x}}$, as desired. Also, two daughter processes share a proportion c_{x} of spike times (since the probability that a spike time in one process also appears in the other process is c_{x}).

It will be more convenient to group all the supporting text in one file with several sections.

Thank you for the suggestion. We have now combined all Supporting Information into one file.

References

[1] Okun M, Lampl I. Instantaneous correlation of excitation and inhibition during ongoing and sensory-evoked activities. Nature Neuroscience. 2008;11:535–537.

[2] Cohen MR, Kohn A. Measuring and interpreting neuronal correlations. Nat Neurosci. 2011;14:811–819.

[3] Smith MA, Jia X, Zandvakili A, Kohn A. Laminar dependence of neuronal correlations in visual cortex. J Neurophysiol. 2013;109:940–947.

[4] Ecker A, Berens P, Cotton RJ, Subramaniyan M, Denfield GH, Cadwell C, et al. State dependence of noise correlations in macaque primary visual cortex. Neuron. 2014;82:235–248.

[5] Tan A, Chen Y, Scholl B, Seidemann E, Priebe NJ. Sensory stimulation shifts visual cortex from synchronous to asynchronous states. Nature. 2014;509:226–229.

[6] McGinley MJ, Vinck M, Reimer J, Batista-Brito R, Zagha E, Cadwell CR, et al. Waking state: rapid variations modulate neural and behavioral responses. Neuron. 2015;87:1143–1161.

[7] Atallah B, Scanziani M. Instantaneous modulation of gamma oscillation frequency by balancing excitation with inhibition. Neuron. 2009;19:1690–96.

[8] Barral J, Reyes AD. Synaptic scaling rule preserves excitatory-inhibitory balance and salient neuronal network dynamics. Nat Neurosci. 2016;19:1690–1696.

[9] Dehghani N, Peyrache A, Telenczuk B, Quyen MLV, Halgren E, S Cash NH, et al. Dynamic Balance of Excitation and Inhibition in Human and Monkey Neocortex. Sci Rep. 2016;6:23176.

[10] Galarreta M, Hestrin S. Frequency-dependent synaptic depression and the balance of excitation and inhibition in the neocortex. Nature Neurosci. 1998;1:587–94.

[11] Yizhar O, Fenno L, Prigge M, Schneider F, Davidson T, O'Shea D, et al. Neocortical excitation/inhibition balance in information processing and social dysfunction. Nature. 2011;477:171–8.

[12] Zhou M, Liang F, Xiong X, Li L, Li H, Xiao Z, et al. Scaling down of balanced excitation and inhibition by active behavioral states in auditory cortex. Nature Neurosci. 2014;17:841–50.

[13] van Vreeswijk C, Sompolinsky H. Chaotic Balanced State in a Model of Cortical Circuits. Neural Computation. 1998 jul;10(6):1321–1371.

[14] Van Vreeswijk C, Sompolinsky H. Chaos in Neuronal Networks with Balanced Excitatory and Inhibitory Activity. Science. 1996;274(5293):1724–1726. Available from: `www.sciencemag.org`.

[15] Haider B, Duque A, Hasenstaub A, McCormick D. Neocortical network activity in vivo is generated through a dynamic balance of excitation and inhibition. J Neurosci. 2006;26:4535–45.

[16] Heiss J, Katz Y, Ganmor E, Lampl I. Shift in the balance between excitation and inhibition during sensory adaptation of S1 neurons. The Journal of Neuroscience. 2008;28:13320–30.

[17] Xue M, Atallah B, Scanziani B. Equalizing excitation-inhibition ratios across visual cortical neurons. Nature. 2014;511:596–600.

[18] Okun M, Lampl I. Balance of excitation and inhibition. Scholarpedia. 2009;4:7467.

[19] Renart A, De La Rocha J, Bartho P, Hollender L, Praga N, Reyes A, et al. The Asynchronous State in Cortical Circuits. Science. 2010 feb;327(2):587–590.

[20] Wimmer K, Compte A, Roxin A, Peixoto D, Renart A, de la Rocha J. The dynamics of sensory integration in a hierarchical network explains choice probabilities in MT. Nat Commun. 2015;6:1–13.

[21] Mochol G, Hermoso-Mendizabal A, Sakata S, Harris KD, de la Rocha J. Stochastic transitions into silence cause noise correlations in cortical circuits. Proc Natl Acad Sci USA. 2015;112.

[22] Huang C, Ruff DA, Pyle R, Rosenbaum R, Cohen MR, Doiron B. Circuit Models of Low-Dimensional Shared Variability in Cortical Networks. Neuron. 2019;101(2):337–348. Available from: https://doi.org/10.1016/j.neuron.2018.11.034.

[23] Baker C, Ebsch C, Lampl I, Rosenbaum R. Correlated states in balanced neuronal networks. Phys Rev E. 2019;99:052414.

[24] Rosenbaum R, Smith MA, Kohn A, Rubin JE, Doiron B. The spatial structure of correlated neuronal variability. Nature Neuroscience. 2017;20(1):107–114.

[25] Landau I, Sompolinsky H. Coherent chaos in a recurrent neural network with structured connectivity. PLOS Computational Biology. 2018;14.

[26] Mastrogiuseppe F, Ostojic S. Linking Connectivity, Dynamics, and Computations in Low-Rank Recurrent Neural Networks. Neuron. 2018;99:609–23.

[27] Darshan R, van Vreeswijk C, Hansel D. How strong are correlations in strongly recurrent neuronal networks? Physical Review X. 2018;8.

[28] N Shaham YB. Slow diffusive dynamics in a chaotic balanced neural network. PLoS Comp Biol. 2017;.

[29] Morrison A, Aertsen A, Diesmann M. Spike-timing-dependent plasticity in balanced random networks. Neural Computation. 2007;19:1437–1467.

[30] Kempter R, Gerstner W, Hemmen LV. Hebbian learning and spiking neurons. Physical Review E. 1999;59:4498–4514.

[31] Bi G, Poo M. Synaptic modification of correlated activity: Hebb's postulate revisited. Annu Rev Neurosci. 1998;24:139–66.

[32] Markram H, Lubke J, Frotscher M, Sakmann B. Regulation of synaptic efficacy by coincident postsynaptic aps and epsps. Science. 1997;275:213.

[33] Izhikevich E, Gally J, Edelman G. Spike-timing dynamics of neuronal groups. Cerebral Cortex. 2004;14:933–44.

[34] Babadi B, Abbott L. Pairwise Analysis Can Account for Network Structures Arising from Spike-Timing Dependent Plasticity. PLoS Comput Biol. 2013;9:1002906.

[35] Litwin-Kumar A, Doiron B. Formation and maintenance of neuronal assemblies through synaptic plasticity. Nature Communications. 2014;5:5319.

[36] Gütig R, Aharonov R, Rotter S, Sompolinsky H. Learning input correlations through nonlinear temporally asymmetric Hebbian plasticity. J Neurosci. 2003;23:3697–714.

[37] Burkitt A, Gilson M, van Hemmen J. Spike-timing-dependent plasticity for neurons with recurrent connections. Biological Cybernetics. 2007;96:533–546.

[38] Gilson M, Burkitt A, Grayden D, Thomas D, van Hemmen J. Emergence of network structure due to spike-timing-dependent plasticity in recurrent neuronal networks. II. Input selectivity-symmetry breaking. Biol Cybernetics. 2009;101:103–114.

[39] Gilson M, Burkitt A, Grayden D, Thomas D, van Hemmen J. Emergence of network structure due to spike-timing-dependent plasticity in recurrent neuronal networks. III. Partially connected neurons driven by spontaneous activity. Biol Cybernetics. 2009;101:411–26.

[40] Gilson M, Burkitt A, Grayden D, Thomas D, van Hemmen J. Emergence of network structure due to spike-timing-dependent plasticity in recurrent neuronal networks. V: self-organization schemes and weight dependence. Biol Cybernetics. 2010;103:365–386.

[41] Vogels TP, Sprekeler H, Zenke F, Clopath C, Gerstner W. Inhibitory Plasticity Balances Excitation and Inhibition in Sensory Pathways and Memory Networks. Science. 2011 dec;334(6062):1569–1573.

[42] Sprekeler H. Functional Consequences of inhibitory plasticity: homeostasis, the excitation-inhibition balance and beyond. Current Opinion in Neurobiology. 2017;49:198–203.

[43] Trousdale J, Hu Y, Shea-Brown E, Josic K. A generative spike train model with time-structured higher order correlations. Frontiers in Computational Neuroscience. 2013;7:84.

[44] Ocker G, Litwin-Kumar A, Doiron B. Self-organization of microcircuits in networks of spiking neurons with plastic synapses. PLOS Computational Biology. 2015;11.

[45] Gilson M, Burkitt A, Grayden D, Thomas D, van Hemmen J. Emergence of network structure due to spike-timing-dependent plasticity in recurrent neuronal networks. I. Input selectivity-strengthening correlated input pathways. Biol Cybernetics. 2009;101:81–102.

[46] Rosenbaum R, Doiron B. Balanced Networks of Spiking Neurons with Spatially Dependent Recurrent Connections. Phys Rev X. 2014;4:021039.

[47] Landau I, Egger R, Dercksen V, Oberlaender M, Sompolinsky H. The Impact of Structural Heterogeneity on Excitation-Inhibition Balance in Cortical Networks. Neuron. 2016;92:1106–21.

[48] Pyle R, Rosenbaum R. Highly connected neurons spike less frequently in balanced networks. Phys Rev E. 2016;93:040302.

[49] Hebb D. The Organization of Behavior. Wiley; 1949.

[50] Kohonen T. Self-Organization and Associative Memory. Springer-Verlag; 1984.

[51] Graupner M, Wallisch P, Ostojić S. Natural Firing Patterns Imply Low Sensitivity of Synaptic Plasticity to Spike Timing Compared with Firing Rate. J Neurosci. 2016;36:11238–58.

[52] Ebsch C, Rosenbaum R. Imbalanced amplification: A mechanism of amplification and suppression from local imbalance of excitation and inhibition in cortical circuits. PLoS Computational Biology. 2018 mar;14(3).

[53] Ocker G, Josić K, SheaBrown E, Buice M. Linking structure and activity in nonlinear spiking networks. PLoS Comput Biol. 2017;13:e1005583.

[54] Kohonen T. Physiological interpretation of the Self-Organizing Map algorithm. Neural Networks. 1993;6:895–905.

[55] Kohonen T. The self-organizing map. Proceedings of the IEEE. 1990;78:1464–80.

[56] Gerstner W, Kistler W, Naud R, Paninski L. Neuronal Dynamics: From single neurons to networks and models of cognition and beyond. Cambridge University Press; 2014.

[57] Zenke F, Gerstner W. Hebbian plasticity requires compensatory processes on multiple timescales. Philos Trans R Soc Lond B Biol Sci. 2017;372:1715.

[58] Zenke F, Gerstner W, Ganguli S. The temporal paradox of Hebbian learning and homeostatic plasticity. Current Opinion in Neurobiology. 2017;43:166–176.

[59] Bienenstock E, Cooper L, Munro P. Theory for the development of neuron selectivity: orientation specificity and binocular interaction in visual cortex. J Neurosci. 1982;2:32–48.

[60] Pfister J, Gerstner W. Triplets of Spikes in a Model of Spike-Timing Dependent Plasticity. J Neurosci. 2006;26:9673–82.

[61] Gjorgjieva J, Clopath C, Audet J, Pfister J. A triplet spike-timing-dependent plasticity model generalizes the Bienenstock-Cooper-Munro rule to higher-order spatiotemporal correlations. PNAS. 2011;108:19383–8.

[62] Shouval H, Bear M, Cooper L. A unified model of NMDA receptor-dependent bidirectional synaptic plasticity. PNAS. 2002;99:10832–6.

[63] Graupner M, Brunel N. Calcium-based plasticity model explains sensitivity of synaptic changes to spike pattern, rate, and dendritic location. PNAS. 2012;109:3991–6.

[64] Froemke RC. Plasticity of Cortical Excitatory-Inhibitory Balance. Annual Review of Neuroscience. 2015;38:195–219.

[65] Petersen C, Malenka R, Nicoll R, Hopfield J. All-or-none potentiation at CA3-CA1 synapses. PNAS. 1998;95:4732–7.

[66] Froemke R, Tsay I, Raad M, Long J, Dan Y. Contribution of Individual Spikes in Burst-Induced Long-Term Synaptic Modification. J Neurophys. 2006;95:1620–9.

[67] Mongillo G, Rumpel S, Loewenstein Y. Inhibitory connectivity defines the realm of excitatory plasticity. Nature Neuroscience. 2018;21(10):1463–1470. Available from: https://doi.org/10.1038/s41593-018-0226-x.

[68] Song S, Sjöström P, Reigl M, Nelson S, Chklovskii D. Highly Nonrandom Features of Synaptic Connectivity in Local Cortical Circuits. PLOS Biology. 2005;3:10.

[69] Vegue M, Roxin A. Firing rate distributions in spiking networks with heterogeneous connectivity. Phys Rev E. 2019;100.

[70] Chiu C, Martenson J, Yamazaki M, Natsume R, Sakimura K, Tomita S, et al. Input-Specific NMDAR-Dependent Potentiation of Dendritic GABAergic Inhibition. Neuron. 2018;97:368–377.

[71] Agnes E, Luppi A, Vogels T. Complementary inhibitory receptive fields emerge from synaptic plasticity and create an attentional switch in sensory circuits. bioRxiv. 2019;.

[72] Hennequin G, Agnes EJ, Vogels TP. Inhibitory Plasticity: Balance, Control, and Codependence. Annual Review of Neuroscience. 2017;40:557–579. Available from: https://doi.org/10.1146/annurev-neuro-072116-.

[73] J Bono CC. Synaptic plasticity onto inhibitory neurons as a mechanism for ocular dominance plasticity. PLoS Comp Biol. 2019;.

[74] Wilmes K, Clopath C. Inhibitory microcircuits for top-down plasticity of sensory representations. Nature Communications. 2019;10:5055.

[75] Tannenbaum NR, Burak Y. Shaping Neural Circuits by High Order Synaptic Interactions. PLOS Comp Biol. 2016;.

[76] Macke J, Berens P, Ecker A, Tolias A, Bethge M. Generating spike trains with specified correlation coefficients. Neural Comput. 2009;21:397–423.

[77] Montangie L, Miehl C, Gjorgjieva J. Autonomous emergence of connectivity assemblies via spike triplet interactions. PLoS Comp Biol. 2020;.
