# Supplementary material for: Balanced networks under spike-time dependent plasticity
Source: PLoS Comput Biol. 2021 May 12;17(5):e1008958. doi: 10.1371/journal.pcbi.1008958 (PMC8143429; doi:10.1371/journal.pcbi.1008958)
Supplement: Attachment — Submitted filename: Referee C comments.pdf [file pcbi.1008958.s003.pdf]

The manuscript addresses the dynamics of plasticity under STDP in randomly connected neural networks in the balanced regime. The manuscript provides some analytical insights into this topic, backed up by numerical simulations. The topic is important, and results seem substantial and interesting. Nevertheless, there are some significant weaknesses in the analysis and presentation, which I recommend to address before the manuscript is considered again for publication.

Since the theory is derived at the level of mean population activities, there is a hidden assumption that synaptic weights evolve uniformly. Is this true empirically in the simulations? How variable are the weights following plasticity?

Even though mean–field approximations are accurate under uniformity, they can also be accurate when weights and rates are not uniform. Our theory only requires that $\langle J^{ab}_{jk} r^b_k \rangle_{j,k} \approx \langle J^{ab}_{jk} \rangle_{j,k} \langle r^b_k \rangle_k$ where $\langle \cdot \rangle_{j,k}$ is the average over indices j and k. We provide an example of when and how this assumption breaks, and we describe a semi–analytic approach to restore the mean–field theory (Fig. 3).

We have now included supplementary figures that show the uniform evolution of randomly sampled individual weights in Kohonen's, weight–dependent Hebbian, and inhibitory STDP rules in (Supporting Figs. 6 **A**,7 **A**,8 **A**). A longer discussion of this new material is provided in the response to comments by Reviewer A.

Related to this point, there is also an implicit assumption that weights evolve independently of other quantities. One example is the correlation between presynaptic firing rate and the synaptic weight, which is discussed in the section on inhibitory plasticity. But there are other possible correlations that could develop between synaptic strength and, for example: the in-degree and out-degree of involved neurons; under some rules competition between synapses belonging to the same neuron could also break some of the assumptions of the mean field theory.

We agree with this comment. In all of our simulations, the in-degree and out-degree are almost equal, because $p_{ab} \equiv 0.1$ for all $a, b = e, i, x$ and N was large. Different extensions of the theory would have to account for other types correlations between different weights, degrees, and/or activity. We have now cited the work by *Vegué and Roxin* 2019, and commented on it in the Discussion:

> In plastic networks, correlations between weights and other features such as in–degrees, or out–degrees can emerge [69]. We have shown how the theory can capture the case in which synaptic weights and pre–synaptic rates are correlated. While we were not able to find analytical expressions for these correlations, we showed that a second–order correction is sufficient to explain the observed dynamics. Eventually, the mean–field theory would need to be extended to account for higher order network motifs and their potential correlations with synaptic weights and firing rates. This might be possible by extending our approach, but we leave these extensions for future work.

A full theory of the plasticity dynamics should address these questions, but I accept that there is value in deriving the predictions of a mean field theory that ignores such correlations, while comparing the predictions with numerical results. The presentation should acknowledge these limitations more explicitly. The appearance of a discussion of correlations between firing rates and synapses only in the section on inhibitory plasticity is unsatisfactory.

We agree with this point and have added it to the Discussion.

> In plastic networks, correlations between weights and other features such as in–degrees, or out–degrees can emerge [69]. We have shown that the theory applies to the case in which synaptic weights and pre–synaptic rates are correlated. While we were not able to find analytical expressions for these correlations, we showed that a second–order correction is sufficient to explain the observed dynamics. Eventually, the mean–field theory would need to be extended to account for higher order network motifs and their potential correlations with synaptic weights and firing rates.

We have also added the following text to the Methods:

> Higher order interactions are at the heart of triplet rules [59–61, 77], and other types of interactions may also be important, *e.g.* for calcium–based update rules [62, 63]. For simplicity we here focus on pairwise interactions between spikes and eligibility traces, and leave extensions to more complex rules for future work.

Is there a way to understand why correlations did not play an important role in earlier sections?

This is an important question, and we have added a paragraph to the Discussion to address this more clearly.

> A natural question that arises is why do correlations between weights and pre–synaptic rates only seem to play a role in iSTDP? In the examples of excitatory STDP we analyzed (Kohonen's rule and weight–dependent Hebbian rule), weights at equilibrium are determined by other parameters (Weight–dependent Hebbian rule) or rates (Kohonen's rule). Therefore weights are updated until those steady state values are achieved, yielding values independent of initial conditions. On the other hand, in the case of the inhibitory plasticity rule, inhibitory weights at equilibrium are determined by the firing rates alone. Since the firing rate vectors are lower–dimensional than the weight matrices, the equilibrium solution does not fully determine the weight matrices. This is shown in Fig 3 **C** inset, where different distributions of weights

can result in the same equilibrium firing rate when weights and pre–synaptic rates become correlated.

While the theory is derived for dynamics of the synaptic weights, the numerical analysis does not demonstrate that the dynamics indeed follow equation 6. Instead, simulations are used only to demonstrate agreement with the predictions on the steady state. Do the dynamics follow the prediction?

We agree. The system of equations can be solved iteratively over time as well, provided the timescales are separated. We have now included this in S1 Appendix: "Transient dynamics of synaptic weights".

Transient dynamics of synaptic weights. In much of this work we focused on steady state behavior. However, the system of equations (Eqs. (14,21,71)) relating rates, covariances, and weights can also be solved iteratively over time, provided the timescales are well separated. Here, we show three examples: weight–dependent Hebbian STDP, Kohonen's rule, and iSTDP. In all three cases, the observed trajectory of synaptic weights closely follows the theoretical predictions (Supporting Figs. 3 **A–C**).

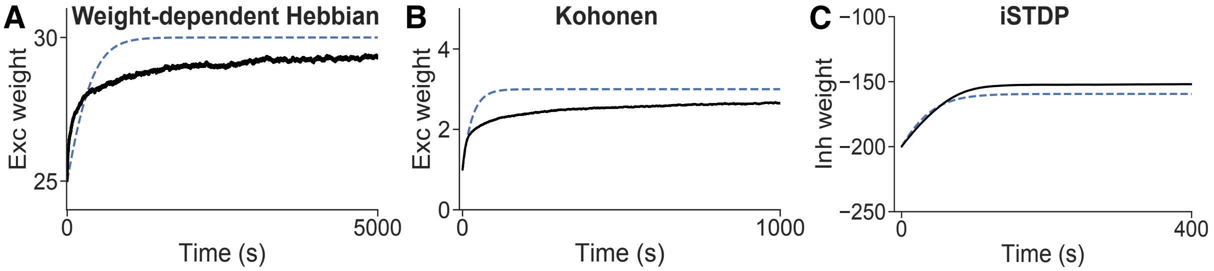

Supporting Figure 3: **Transient dynamics of synaptic weights. A:** Mean synaptic weight in a network subject to "weight–dependent" Hebbian STDP. **B:** Same as **A**, but for Kohonen STDP. **C:** Same as **A**, but for inhibitory STDP. In all panels, dashed lines represent the theory (Eqs. (14,21,68)); solid lines represent numerical simulations.

I would also like to see some numerical support for statements that the network remains in an asynchronous state following plasticity.

We agree that it is important to demonstrate this. We have added a new section to S1 Appendix, where we analyze the distribution of synaptic weights, rates, and covariances. We also show that the network is balanced throughout. Supporting Figs. 6–8 **E,F** show that mean covariances are very weak, *i.e.*, the network remains in the asynchronous state.

The figures are included earlier in the letter, and we repeat the new text here for convenience:

> **Statistics and dynamics of balanced networks under pairwise STDP rules.** Our theoretical results describe the evolution of averages (See Methods in main text). However, it is also important to understand changes in higher order statistics of individual, and collective activity under plasticity. Although we do not go beyond a mean–field theory, we provide the results of corresponding numerical simulations in three example balanced networks where: (1) excitatory weights were subject to weight–dependent Hebbian STDP; (2) *EE* weights changed according to Kohonen's rule; and (3) inhibitory weights changed according to an iSTDP rule (See Table 1 in main text for equations describing each rule).
>
> Under weight–dependent Hebbian STDP individual synaptic weights converge to a unimodal distribution. This is in agreement with experimental results [68] (Supporting Fig. 6 **A,B**). Balance is preserved throughout the simulation (Supporting Fig. 6 **C**). Distributions of rates are unimodal and skewed to the right (Supporting Fig. 6 **D** and inset), also agreeing with experimental findings [68]. Spike count covariances and correlations are distributed around zero in the asynchronous state (Supporting Fig. 6 **E,F**).
>
> *EE* weights under Kohonen's rule also converge to a unimodal distribution (Supporting Fig. 7 **A,B**). Again, the network is stable and balance is preserved (Supporting Fig. 7 **C**). The distributions of rates is again unimodal and skewed to the right (Supporting Fig. 7 **D**), and covariances and correlations are close to zero (Supporting Fig. 7 **E,F**). Lastly, *EI* weights subject to an inhibitory plasticity rule also follow a unimodal distribution (Supporting Fig. 8 **A,B**), distributions of *I* rates are unimodal and skewed to the right, and covariances and correlations are centered at zero in the balanced, asynchronous state (Supporting Fig. 8 **C–F**). However, the *E* rates are concentrated at the target rate (Supporting Fig. 8 **D** – inset), in disagreement with experimental findings [68]. In short, all three STDP rules show realistic distributions of weights, rates, and covariances, except for the iSTDP's distribution of *E* rates.

Lines 359-365: What happens under the perturbation without plasticity? How does this depend on parameters? How can we see that the network is in a balanced state following plasticity (but possibly not before plasticity)?

After external input onset, the network initially exits the balanced state when some E neurons are silenced due to excess average inhibitory input (Fig. 4 **D**). However, while targeted cells still receive optogenetic input, inhibitory weights adjust to restore detailed balance in the network. The network will remain at or return to the balanced state so

long as parameters are chosen such that W is non–singular. Thus a prolonged input will drive the network to a new balanced state, while a transient input may drive the network out of balance for a short period.

We now clarify this point in the following:

> We simulated a network of $N = 10^4$ neurons in an asynchronous state with $c_{x_1} = c_{x_2} = 0$. A subpopulation of 4000 E cells receives transient, correlated input. Solving Eqs. (27,28) predicts that inhibitory plasticity will alter EI synaptic weights so that the firing rates of both the E_{expr} and the $E_{\text{non-expr}}$ approach the target firing rate before, during, and after stimulation. Once the network reaches steady state the mean inputs to each subpopulation cancel. Thus changes in EI weights restore balance at the level of individual subpopulations or "detailed balance," consistent with previous studies [41, 72]. Simulations confirm these predictions (Fig. 4 **B–D**).
>
> When the input is removed, the inhibitory weights onto cells in the E_{expr} subpopulation converge to their pre–stimulus values, returning E_{expr} rates to the target value, and reestablishing balance (Fig. 4 **B–D**). Correlations remain low ($\mathcal{O} \sim 10^{-4}$) before, during, and after stimulation (Fig. 4 **E,F**), suggesting that at equilibrium the network is in the asynchronous state. Our theory thus describes how homeostatic inhibitory STDP increases the stability and robustness of balanced networks to perturbations by balancing inputs at a level of individual cells, maintaining balance in regimes in which non–plastic networks cannot maintain balance. We presented an example in which only one subpopulation is simulated. However, the theory can be extended to any number of subpopulations in asynchronous or correlated balanced networks receiving a variety of transient stimulus.

In comparisons between simulations and the theory, for how long were the simulations run? What was the criterion used to identify that the weights reached steady state?

We have added these details in the captions throughout the text. We applied the Kolmogorov–Smirnov 2–sample test to determine if synaptic weights had reached equilibrium. We added text clarifying this in "Appendix: Determining when synaptic weights reach equilibrium in simulations".

> **Determining when synaptic weights reach equilibrium in simulations.** To determine if synaptic weights had reached steady state we sampled 1000 synaptic weights from the whole population (of plastic weights) at times $t = 4000$sec and $t = 5000$sec (end of simulation). We then compared the two distributions using the Kolmogorov–Smirnov 2–sample test. We found that in the three networks simulated in Supporting Figs. 6–8, the distributions were not distinguishable, and hence had reached equilibrium by the end of the simulation (Supporting Fig. 4 **A–C**)

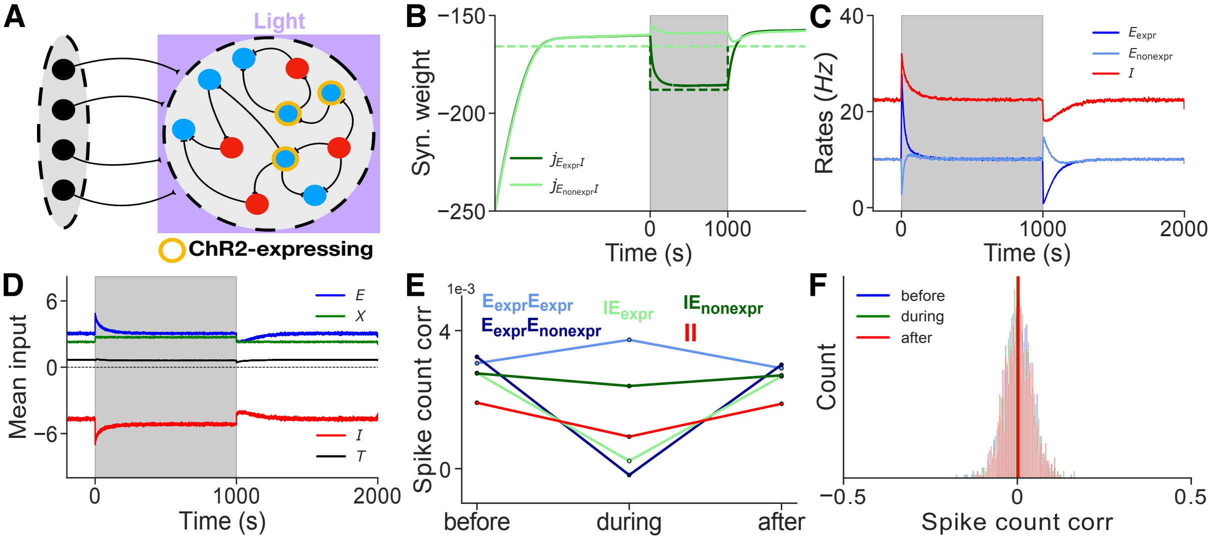

Figure 4: **General framework for STDP in balanced networks describes the dynamics of networks receiving optogenetic input.** **A:** A recurrent network of excitatory, E, and inhibitory, I, neurons is driven by an external feedforward layer X_1 of uncorrelated Poisson neurons. Neurons that express ChR2 are driven by optogenetic input, which is modeled as an extra layer of Poisson neurons denoted by X_2. **B:** Evolution of mean synaptic weights over the course of the experiment. **C:** Evolution of mean firing rates. Inhibitory STDP maintains E rates near the target, $\frac{\alpha_e}{2\tau_{\mathrm{STDP}}}$. **D:** Evolution of mean excitatory, external, inhibitory, and total currents. Balance is transiently disrupted at stimulus onset and offset, but it is quickly restored by iSTDP. **E:** Mean spike count correlations before, during, and after stimulation remain very weak for all pairs. **F:** The distribution of spike count correlations also remains nearly unchanged with weak mean correlations before, during, and after stimulation. Solid lines represent simulations, dashed lines are values obtained from theory (Eqs. (3,5,27,28)).

Line 6: it will be appropriate to cite also Shaham and Burak (2017) and Darshan et al (2017) in the context of spatially correlated activity in the balanced state.

The introduction has been rewritten to more appropriately place the work in the balanced network and synaptic plasticity literature and these references have now been added.

> Cortical neuronal activity is typically irregular, correlated, and can be dominated by a low dimensional component [1–6]. Cortical activity is also characterized by a balance between excitation and inhibition [1,7–12]. Such balance is now a widely thought to give rise to stable, irregular neural activity [1,7,12–18]. Early theoretical work has focused on irregular asynchronous dynamics, with large networks exhibiting vanishing correlations [13,19]. However, more recent extensions have shown how correlated dynamics can be generated both endogenously and exogenously, while preserving irregular single cell activity [20–28], showing the existence of both asynchronous and correlated states in balanced

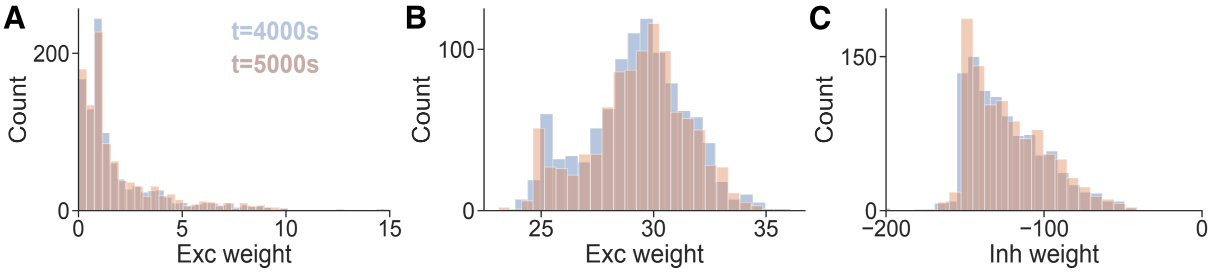

Supporting Figure 4: **Distributions of synaptic weights are stationary.** **A:** Distributions of EE synaptic weights for a balanced network with excitatory weights evolving according to Kohonen's rule at times $t = 4000$sec and $t = 5000$sec. The two distributions are not different according to the Kolmogorov–Smirnov 2–sample test ($D_n = 0.029$, p–value$= 0.8$). Hence, EE weights are at steady state. **B:** Same as **A**, but for a network with weights that follow weight–dependent Hebbian STDP showing excitatory weights are in equilibrium ($D_n = 0.045$, p–value$= 0.26$). **C:** Same as **A**, but for a network where EI weights are subject to iSTDP. Inhibitory weights are in equilibrium ($D_n = 0.024$, p–value$= 0.94$). All simulation parameters as in Supporting Figs. 6–8.

networks.

In the discussion, it will be appropriate to cite Ravid-Tannenbaum and Burak (2016) in the context of the relationship between covariances and STDP dynamics.

Thank you. We have now included the missing reference.

Ocker et al. also studied the dynamics of mean synaptic weights in recurrent networks and derived equations for mean synaptic weights in terms of rates and covariances [44, 75].

Lines 58-59: why is the assumption not essential? How does this agree with Ref. 34, which shows that heterogeneity in the connection probabilities can destroy the balanced state?

We meant that this assumption is not essential as long as the matrix W is non–singular. Balance can be maintained or destroyed depending on several factors, and heterogenous connectivity may not necessarily disrupt it (Pyle PRE, 2016). We have modified our statement to clarify this.

For simplicity we assume that both the probabilities, p_{ab}, and weights, $j_{ab} \sim \mathcal{O}(1)$ are constant across pairs of subpopulations. This assumption is not essential as long as the balance condition remains valid. This is equivalent to having a non–singular mean–field connectivity matrix $\overline{W}$, which is defined next.

PRESENTATION

It is inconvenient that only some of the equations are numbered. This makes it difficult to refer to the unnumbered equations when discussion the manuscript.

We have now numbered all the equations for the readers' convenience.

Equation 2: please define Gamma precisely, not only in limits, and either derive this result or provide a clear path from the results of previous papers to this expression, possibly in an appendix.

We now point to detailed derivations of Γ already presented in Baker et al., 2019. A closed form for finite N is not tractable for spiking neural networks.

> The matrix Γ has the same structure as C and represents the covariance between external inputs (See Baker et al., 2019 Appendix A for a detailed derivation of this term [23]).

The role of Twin in equation 2 is not sufficiently clear. If I understood this correctly, Twin should be introduced already when defining the spike count covariance.

We agree with this comment and now define T_{win} earlier.

> We define the mean spike count covariance matrix as:
>
> $$C = \begin{bmatrix} C_{\text{ee}} & C_{\text{ei}} \\ C_{\text{ie}} & C_{\text{ii}} \end{bmatrix}$$
>
> where C_{ab} is the mean spike count covariance between neurons in populations $a = \text{e}, \text{i}$ and $b = \text{e}, \text{i}$ respectively, counted over time windows of size T_{win}. Throughout all simulations and theoretical predictions, we set $T_{\text{win}} = 250\text{ms}$, however the theory is flexible to other time window sizes.

Lines 73-74: the statement that the second term in Equation 2 accounts for intrinsically generated variability is a bit confusing since the elements in the diagonal are negative. It will be helpful to clarify this.

We realize how this was confusing. We now remind the reader that in the spike count covariance matrix the diagonal terms are not variances, but the mean covariances between spike trains in the same population. We have clarified this in the following added text:

In Eq. (5), F_a is the Fano factor of the spike counts averaged over neurons in populations $a = \mathrm{e}, \mathrm{i}$ over time windows of size T_{win}. The second term in Eq. (5) is $\mathcal{O}(1/N)$ and accounts for intrinsically generated covariability [23] within excitatory or inhibitory populations (note that this term does not refer to variances, but instead to mean covariances between spike trains in the same subpopulations).

The logic of how x and S combine to generate the STDP rule is mathematically simple, yet for many readers it will be difficult to understand the structure of the rule from the equations. It will be helpful to provide a graphical representation of the STDP rules in the different cases listed in Table 1.

We agree with this comment. We have now added Supporting Fig. 1 to show the STDP window for each rule presented in Table 1.

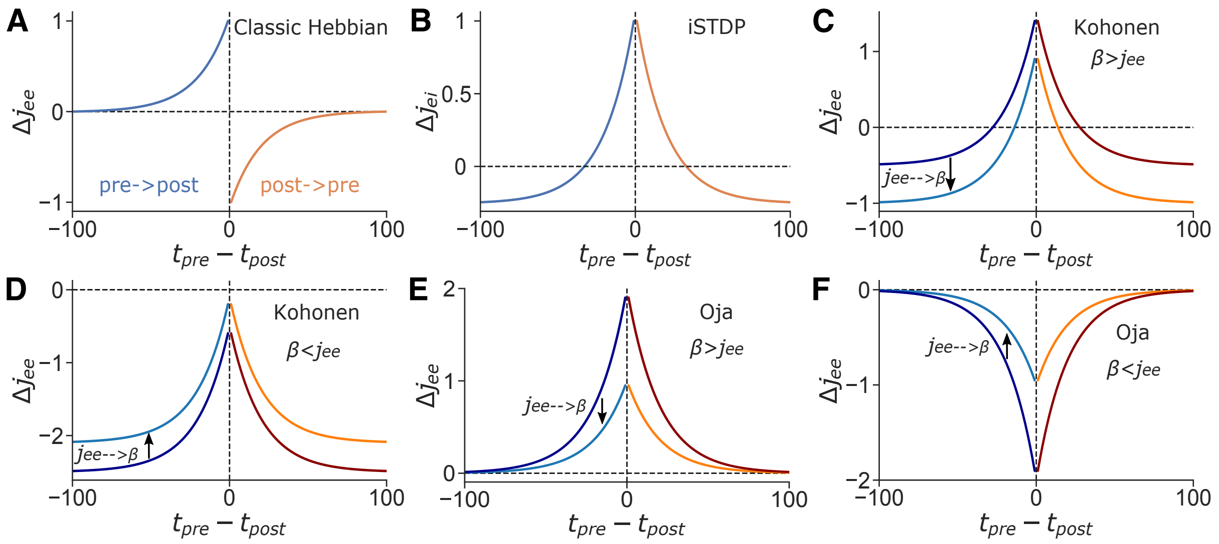

Supporting Figure 1: **STDP windows of different plasticity rules. A:** Change in synaptic weights as a function of the relative timing of pre– and post–synaptic spikes in Classical Hebbian STDP (same as weight–dependent Hebbian). **B:** Same as **A**, but for inhibitory STDP. **C:** Same as **A**, but for Kohonen's rule when weights are below parameter β. **D:** Same as **C**, but for the case when weights are above β. **E:** Same as **A**, but for Oja's rule when weights are below parameter β. **F:** Same as **E**, but for the case when weights are above β.

Lines 140-148: I didn't understand why root finding is involved in the numerical solution of the differential equations. Doesn't equation 6 simply provide the required update of J, based on the values of r anc C that are obtained from equations 1-2?

We realize this was not clear before and have addressed this now.

This process can be repeated iteratively at each time step to obtain the evolution of firing rates, spike count covariances, and synaptic weights provided our assumption about the separation of timescales holds (See S1 Appendix: "Transient dynamics of synaptic weights" for more details).

Figure 3: "and the system predicts a stable fixed point". Should "system" be replaced by "theory"?

Yes, thank you for pointing that out.

Fig 3: **A:** The rate of change of EI weights as a function of the weights themselves. The contributions of the covariance (blue) is considerably smaller than the contribution of the rate (red), and the theory predicts a stable fixed point.

Line 339: sentence ends prematurely.

Thank you for catching this.

We show that inhibitory STDP, as described by Eq. (22), can restore balance in the inputs to the stimulated and unstimulated subpopulations.

The last equation in page 1 of SI1 includes parameters whose meaning is not defined.

Thank you for noticing that. We have now pointed to the specific table containing these parameters.

See Supporting Table 1 for a description and values of the parameters of the voltage dynamics. In our simulations, this equation was integrated using Forward Euler's method with step size $dt = 0.1$ms.

In line 26 of SI1, units for dt are missing.

Thank you for pointing that out. We now specify units: $dt = 0.1$ms

In line 53 of SI1, in the equation $rm = r_x/cx$, should cx be c_x? (where _ stands for subscript).

Yes, thank you. We have now fixed this typo:

> To generate N_x processes, all having firing rate r_x and pairwise correlation c_x, we first generate a "mother" process with rate $r_m = r_x/c_x$.

Lines 53-56: It is not easy to understand from the description how the daughter process spikes are obtained. I suggest to clarify that each daughter process is obtained by first taking all the spikes of the mother process, followed by a deletion of spikes with probability $1 - c_x$, and to mention explicitly that deletion is performed independently in each one of the daughter processes.

We agree with this comment, and have made this point more clear in the text.

> To generate N_x processes, all having firing rate r_x and pairwise correlation c_x, we first generate a "mother" process with rate $r_m = r_x/c_x$. Then, to generate each of the N_x "daughter" processes, we first take all the spikes in the mother process and then delete each spike independently with probability $1 - c_x$. In other words, each spike time from the mother process is included in each daughter process independently with probability c_x. The rates of the daughter processes are $r_m c_x = r_x$, as desired. Also, two daughter processes share a proportion c_x of spike times (since the probability that a spike time in one process also appears in the other process is c_x).

It will be more convenient to group all the supporting text in one file with several sections.

Thank you for the suggestion. We have now combined all Supporting Information into one file.

References

[1] Okun M, Lampl I. Instantaneous correlation of excitation and inhibition during ongoing and sensory-evoked activities. Nature Neuroscience. 2008;11:535–537.

[2] Cohen MR, Kohn A. Measuring and interpreting neuronal correlations. Nat Neurosci. 2011;14:811–819.

[3] Smith MA, Jia X, Zandvakili A, Kohn A. Laminar dependence of neuronal correlations in visual cortex. J Neurophysiol. 2013;109:940–947.

[4] Ecker A, Berens P, Cotton RJ, Subramaniyan M, Denfield GH, Cadwell C, et al. State dependence of noise correlations in macaque primary visual cortex. Neuron. 2014;82:235–248.
