# Supplementary material for: Balanced networks under spike-time dependent plasticity
Source: PLoS Comput Biol. 2021 May 12;17(5):e1008958. doi: 10.1371/journal.pcbi.1008958 (PMC8143429; doi:10.1371/journal.pcbi.1008958)
Supplement: Attachment — Submitted filename: rebuttal_letter_second_round.pdf [file pcbi.1008958.s004.pdf]

Summary

We thank the editor and the referees for their careful reading of the manuscript and helpful suggestions. We have further revised the manuscript in answer to these comments. Below we provide detailed answers to the remaining questions, and describe changes to the manuscript in response to these comments.

The reviewers made important points about the need to better compare our work with existing literature, and to correct typos, redundancies, and missing references. We have changed the text and the Supplementary Material to address these concerns. We hope that the updated manuscript is now suitable for publication in PLoS Computational Biology.

Response to Reviewer's General Comments

Reviewer comments are in italics. Our responses are in plain text. New text added to the manuscript is in blue. The corresponding new text in the manuscript is also highlighted in blue.

Editors

As you can see, the three reviewers are mostly positive about your manuscript and find that it has improved. However, one of the reviewers still has the concern that the approach taken in this work is weaker than in previous works (especially the assumptions of infinite size networks and precise balance, which are more limiting that the assumptions made in other works). Therefore, the authors should make a clear case for the benefits of their approach.

In particular:

- Although the Introduction has improved in terms of putting this manuscript into the context of other published work, the link between descriptions of previous work and the goal of the current work is missing. This could be helped by elaborating more on existing work – beyond just listing specific conditions under which previous frameworks hold (see also next comment).

- There are multiple references missing, some are incorrectly cited, and some equation numbers do not appear correctly (see reviewer comments). For example, the authors write in their response letter that they cited Montangie et al. in the Introduction but they do not. It's certainly more relevant than the studies of triplet STDP in feedforward networks with a single postsynaptic neuron that the authors currently cite, because it focuses on the plasticity of recurrent networks. It could and should be mentioned also in the Discussion where they discuss how their work can be extended to include the impact of second and higher order moments on the evolution of the weights. Another example is the Ravid Tannenbaum and Burak (2016) reference which similarly to Montangie et al (2020) considers correlations generated intrinsically within the network – that other works (e.g. Gilson et al.) do not. These are just some examples of how the authors could better explain how their work fits in the context of published work.

We agree with this point. We have included references to the papers by Ravid Tannenbaum and Burak 2016, and Montangie et al. 2020 in our Introduction, and have pointed out how our work differs from those in the Discussion. The manuscript now has over 100 references, and we tried to give an overview of the most relevant related work.

We only quote here the one paragraph of the Introduction that has considerably changed.

> However, we still lack a general theory that relates STDP to changes in neural activity, and the resulting neural computations. Hence, often the analysis of the effects of STDP relies on simulations [1–4]. Analytical treatments have been proposed in a number of cases, starting with the description of mean synaptic dynamics on a single integrate–and–fire neuron receiving feed–forward input from a collection of Poisson neurons [5]. These results have been extended to small networks [3], and recurrent networks of Poisson neurons [6–9]. Other work provided analytical treatments of specific plasticity rules, such as homeostatic inhibitory plasticity [10, 11]. Using linear response and motif resumming techniques [12], Ocker et al. developed a theory describing the evolution of mean weights in recurrent neural networks of noisy integrate–and–fire neurons under STDP [13]. This approach relies on the assumption that the input to individual cells is dominated by white noise, local synaptic input is weak, and integral of the STDP function is small. Related results were obtained by treating neural firing as a Poisson process [6–8, 14]. In particular, Ravid Tannenbaum et al. showed that in networks of Poisson neurons synfire chains and self connected assemblies can emerge autonomously in recurrent networks [15]. Montangie et al. showed that a more realistic form of STDP based on spike triplets also leads to autonomous emergence of assemblies [16].

And we explained how our theory differs from previous ones in the Discussion:

> A number of mathematical theories have been proposed to describe the co-evolution of weights, rates, and the structure of correlations under STDP in recurrent neural networks [6–8, 13–17]. All of these approaches require knowledge of neurons' transfer functions (f-I curves and/or correlation susceptibility functions). Often neurons are assumed to be Poissonian, and their responses to inputs (f-I curves) are prescribed [6–8, 14–17]. Other work [13] uses Fokker–Planck techniques to compute transfer functions. These approaches rely on an assumption that the input to each neuron is relatively weak or dominated by Gaussian white noise [18, 19] and efficient, direct Fokker–Planck approaches are not available for two–dimensional integrate–and–fire models such as those with adaptation currents, though one–dimensional approximations have been derived [20, 21]. Some previous work [13] also assumes that STDP curves are approximately anti–symmetric, *i.e.*, there is a cancellation between the positive and negative parts of the curves (as in Supporting Figure 1**A**).

> Our approach uses balanced network theory to avoid the computation of neurons' transfer functions. As such, the resulting theory does not require an assumption of weak synaptic interactions or dominant Gaussian white noise input, but can be applied to networks with highly non–Gaussian, temporally correlated input (such as the networks in the correlated state considered here). Moreover, the balanced network theory we used is accurate for a range of neuron models, including those with adaptation currents [22, 23], and for non–anti–symmetric STDP curves (as in Supporting Figure 1**B–F**). However, the balanced network theory used in our approach relies on large N asymptotics, which yielded accurate approximations for $N \sim 10,000$ in our case (Figure 1), but are inaccurate for smaller networks. Moreover, our approach would not be accurate for modeling neural circuits that do not exhibit excitatory–inhibitory balance, such as in some disease states, some developmental stages, and in some sub–cortical neuronal networks. Finally, we used a mean–field approach that only yields approximations to population–averaged firing rates, synaptic weights, and covariances, while other approaches [6–8, 13–17] give approximations to these quantities at the level of individual neurons. Despite these limitations, our analytical approach was sufficient for answering the questions related to the interaction between excitatory–inhibitory balance, correlated neuronal activity, and plasticity that we considered.

We also realized that we failed to add some references in the revised version of the manuscript, although we intended to do so. This was an error on our part which we have now corrected.

For example, we have added the missing references to the following text that proceeds Eqn. (10):

> Higher order interactions are at the heart of triplet rules [16, 24–26], and other interaction types may also be important, *e.g.* for calcium–based update rules [27, 28]. For simplicity we here focus on pairwise interactions between spikes and eligibility traces, and leave extensions to more complex rules for future work.

- *Regarding notation and referencing to equations and figures, it might be a good idea to ensure that the entire paper is consistent as new things are added during the revision.*

Thank you for the note. We have made sure that in this new version the notation and referencing are accurate.

- *Rather than stating all the results at the start of the Results section, the authors should work on providing a map of what they do (and why), rather than what they find. The specifics of what they find are best left to the individual sections where the specific figures are discussed and are currently confusing for such an introductory paragraph. I realize this*

was done in request of reviewer A, but I believe the authors were asked to clarify/outline their approach and goals (rather than state the results before actually presenting them).

The reason for this paragraph was the following comment of referee A from the previous review: "Currently the different sections and applications of the theory come as a surprise." We agree that we took this too literally in the previous version. We have now rewritten this paragraph to describe the goals of the manuscript, rather than recapitulating the results:

> We next apply the theory described in the Methods to show how synaptic weights coevolve with firing rates in balanced networks under different plasticity rules. We start with an example of excitatory plasticity which has been the main focus of experimental and theoretical studies, and show that our theory can be used to determine the stability of balanced networks under commonly used excitatory STDP rules. More recently, inhibitory plasticity has been proposed to play an important role in regulating the dynamics of neural networks. Our approach provides a theoretical foundation for some of these findings. Finally, we show that our theory can be used to make experimental predictions by considering a plastic network under optogenetic stimulation, and demonstrating that our framework can describe the dynamics of networks in such biologically relevant regimes.

- The authors highlighted parts of the paper in blue to indicate that they made changes but often times, only a single sentence in a paragraph is changed, or the order of two words is swapped. This leaves the false impression that many significant changes were made, when in fact, very few, if any changes were made. A clear example is the first paragraph of the Results, where only the first sentence was added and yet the entire paragraph is highlighted in blue. There are many such instances in the paper. It would be better and much clearer for the reviewers and editor to have only specific sentences (or words) that were changed highlighted, as the authors do for example near lines 326-328 and for the remainder of that section.

Our intention was to help reviewers find the paragraphs that were quoted in the rebuttal letter. We therefore sometimes highlighted a paragraph that did not change much. However, we did not intend to give the impression that the revision was more substantial than it was.

We agree that this was confusing, especially considering the large number of edits in response to the earlier comments. In the current revision we have only highlighted text that was changed since the previous version of the manuscript.

Minor:

- What is meant here: "Analytical treatments have been proposed for a number of cases, initially describing the impact of STDP on individual synaptic weights (Guettig)?" Do the

authors mean in feedforward networks, or under some other special condition? Because any plasticity rule describes the impact of STDP on individual synaptic weights. This comment is related to the first comment above, namely to be clear about what other works did, and how this work is different from them.

Thanks for pointing this out. Indeed, we meant in feedforward networks. We have now clarified this point in the Introduction which we quoted above.

> Analytical treatments have been proposed in a number of cases, starting with the description of mean synaptic dynamics on a single integrate–and–fire neuron receiving feed–forward input from a collection of Poisson neurons [5].

- Shouldn't the Results section where the authors introduce the Kohonen rule (around line 221) have a subsection titled something like "Dynamics of balanced networks under the Kohonen rule" to match the corresponding section starting in line 252?

We agree that subsections titles could be improved. We have thus split the section "Balanced networks under excitatory plasticity" into two subsections: "Equilibria of correlated balanced networks under excitatory STDP"; and "Dynamics of correlated balanced networks under excitatory STDP". Please note that in the entirety of the section titled "Balanced networks under excitatory plasticity", we present results on Kohonen's rule only, as stated at the beginning of the section.

- Some redundancies (there are probably others) that should be eliminated to make the narrative clear: e.g. (1) line 218: "Our theory explains the effect of such plasticity rules in balanced networks" and line 220: "the impact of the plasticity rule on the dynamics of the network" (2) lines 37 and 47: "how synaptic plasticity impacts (shapes) network dynamics" (3) lines 131 and 137: "focus on pairwise interactions" vs "considered interaction up to second order".

Thank you for the comment. We have now eliminated such redundancies in the text. Changed text is quoted below.

(1)

> Our theory predicts the stability of the balanced state, the fixed point of the system, and the effect the plasticity rule on the dynamics of the network.

(2) We removed the repetition on line 37.
(3) We erased the repetition on line 137.

We cannot make any decision about publication until we have seen the revised manuscript and your response to the reviewers' comments. Your revised manuscript is also likely to be sent to reviewers for further evaluation.

Referee A

The authors have addressed all my comments from the original review. In the revised form the manuscript is an important contribution to the field, so support publishing it in PLoS CB.

Minor corrections:
Supporting figure 2 C Please change the graphics to indicate more clearly that $\eta = 1$ is shown in the inset only, similar to panel B.

Thank you for the suggestion, we have changed that figure accordingly.

Supporting figure 3 Missing equation cross reference

We fixed this too. Thank you for pointing out these corrections.

Referee B

I think the manuscript has been greatly improved by the authors and my technical concerns were fully answered. Therefore I have no issues with the presented theory which gives us an alternative way of calculating rate-correlation-weights self-consistency, but it is hard for me to see how this approach can be better than previous attempts. In my opinion authors still have to provide a case study to show that this approach could give us a new insight to an open problem or could do the same tasks as the previous models, but better (more efficiently or more correctly).

The reason I believe that this theory cannot do better than previous attempts is because it has a lot of constraints. The most problematic ones are the assumption of limit N tends to infinity, the assumption of precise balance and the assumption that there is a clear separation of time-scales. Those constraints could contradict the essential requirement of biological plausibility.

The theory does not capture dynamics of finite size networks, which are the case of biological neural networks. Does the author assume that the transfer function of single neurons don't play any role? In this respect I am yet to be convinced that this approach would be better then Ocker et al 2015. Especially for calculating rates and correlation self-consistently (Ocker et al. 2016). Is the assumption of infinite networks really weaker than the assumption on the structure of input noise and spike statistics? Until there is a good use case I am inclined to believe that this approach mostly ignores single neuron dynamics and it would be less useful.

Although biological networks keep the EI balance, there is a fundamental question on how do we model it? It is not a priori necessary to keep balance with $1/\sqrt{K}$ scaling. This is the case for current based synapses and this scaling was first introduced for the purpose of theoretical simplification. If we have more biological realistic neurons with conductance based synapses, the balance is kept dynamically. Furthermore, there are networks which

are not necessarily balanced, e.g. in development or diseases. Therefore, I would put more utility in the approach which can treat the problem of self-consistency without the balance condition, but can handle a balanced network as a special case. On this point I would like authors to present a case where their framework works better than Burkit AN et al. 2007, Gilson et al. 2009 or Ocker et. al 2015 approach.

The reviewer makes several related points, which we will address separately first before describing the corresponding changes we made to the manuscript.

> "*The reason I believe that this theory cannot do better than previous attempts is because it has a lot of constraints.*"

All analytical approaches of STDP in spiking networks rely on assumptions, and work under different constraints. The important question, as the reviewer mentions below, is whether our approach offers any advantages over previous approaches, given the constraints of each. We address this question below.

> "*The most problematic ones are the assumption of limit N tends to infinity*"

We used an analytical approach that relies on large N asymptotics. This is quite common in analytical approaches to neuronal network models. For example, it underlies almost all mean-field theories. This does not mean that we assume the networks we are studying are infinite in size, of course, but only that our approximations should become more accurate when N is larger. The relevant question, therefore, is whether the approximations are sufficiently accurate for reasonable values of N. We demonstrated that the approximations are accurate for spiking networks of $N \approx 10,000$ neurons, which is well within a biologically relevant regime. Moreover, previous work on balanced network theory [22,23,29–37] has also verified the accuracy of these approximations for biologically relevant parameters.

> "*the assumption of precise balance*"

Balance is an emergent property of networks with large N when J is scaled so that $J > \mathcal{O}(1/N)$ (or, more precisely, when $J > \mathcal{O}(1/K)$ which is equivalent when $K = Np \sim \mathcal{O}(N)$, where p is the connection probability). Hence, balance should not be considered an assumption of our approach, but a consequence of other assumptions, specifically large N with synaptic weights that are larger than $\mathcal{O}(1/K)$. Moreover, balance becomes more precise in such networks with increasing N. Specifically, the magnitude of excitation and inhibition each grow like $JK > \mathcal{O}(1)$ while their sum (the total input) remains $\mathcal{O}(1)$. Therefore, the precise balance in our asymptotic theory should not be interpreted as an assumption or prediction that balance should be *perfectly* precise in real networks, but only that balance becomes more precise with larger N. Our spiking network simulations are finite so balance is not perfectly precise in those simulations, but our asymptotic theory is still accurate for those simulations, demonstrating the usefulness of large N, precise-balance asymptotics for realistic networks in which balance is not perfectly precise.

"*and the assumption that there is a clear separation of time-scales*"

We assumed that the timescale of long-term plasticity was slower than the timescale of neuronal dynamics. This is a widely used assumption in almost all analytical studies of STDP in spiking networks. Indeed, the same assumption underlies the approaches used in other work cited by the referee such as Burkit et al. 2007, Gilson et al. 2009, and Ocker et. al 2015, and is consistent with the timescales observed in many STDP experiments.

"*Those constraints could contradict the essential requirement of biological plausibility.*"

We verified our analytical approach with spiking network simulations having parameters within a biologically plausible range and the predictions of our theory were accurate for those parameter regimes. We therefore feel that we sufficiently demonstrated the biological plausibility of our approach.

"*The theory does not capture dynamics of finite size networks, which are the case of biological neural networks.*"

As mentioned above, our analysis (like nearly all other mean–field approaches) relies on large N asymptotics, but this does not mean that it cannot capture the dynamics of finite size networks. We explicitly demonstrated that our theory accurately captures the statistics of finite sized spiking network simulations.

"*Does the author assume that the transfer function of single neurons don't play any role? In this respect I am yet to be convinced that this approach would be better then Ocker et al 2015. Especially for calculating rates and correlation self-consistently (Ocker et al. 2016). Is the assumption of infinite networks really weaker than the assumption on the structure of input noise and spike statistics? Until there is a good use case I am inclined to believe that this approach mostly ignores single neuron dynamics and it would be less useful.*"

We address the comparisons to Ocker et al. [13] below and here address the references to single neuron transfer. Specifically, the referee makes reference to the fact that transfer functions do not appear in our analytical expressions for firing rates and correlations. This is a well–known property of balanced network theory that has been used in numerous studies, but it is not an assumption. Rather, it emerges naturally from the mean–field analysis of large networks having synaptic weights that are larger than $\mathcal{O}(1/N)$ (or $\mathcal{O}(1/K)$). To demonstrate how this works, consider the fixed point equation for a rate model,

$$\vec{r} = f(J\vec{r} + \vec{X}),$$

where $\vec{r}$ is the vector of firing rates of every neuron, f is the transfer function, and $\vec{X}$ is the vector of inputs to each cell. Note the average entry in the vector $J\vec{r}$ is a sum of $\mathcal{O}(K)$ non–zero elements each having magnitude $\mathcal{O}(J)$ where K and J are the mean

in-degree and synaptic weight. Similarly, if $\vec{X} = J_{\rm x}\vec{r}_{\rm x}$ comes from an external neural population then it will have a similar scaling. Hence, upon taking averages over e and i sub-populations, one obtains a formula of the form

$$\mathbf{r} = f\left(\overline{JK}[W\mathbf{r} + \mathbf{X}]\right)$$

where $W, \mathbf{X} \sim \mathcal{O}(1)$, $\mathbf{r} = [\overline{r_e} \quad \overline{r_i}]^T$ is a 2×1 vector of mean excitatory and inhibitory firing rates, and similarly for external input $\mathbf{X} = [\overline{X_e} \quad \overline{X_i}]^T$. This can be re-written as

$$W\mathbf{r} + \mathbf{X} = \frac{1}{\overline{JK}} f^{-1}(\mathbf{r})$$

so that, when $\overline{JK}$ is large, the f-I curve has a vanishing effect and

$$\mathbf{r} \approx -\mathbf{W^{-1}X}.$$

A similar approach can be used to show that the transfer functions have vanishing influence on mean–field *covariance* when $\overline{JK}$ is large. Further details about this derivation and its extensions are given in our manuscript, and in previous texts on balanced network theory we cited [22, 23, 29–37]. This is not to say that single–neuron transfer does not "play any role" as asked by the referee, but only that it has a vanishing influence at the mean–field level when $\overline{JK}$ is large to produce an accurate approximation. Importantly, this is not an assumption that we are making, but an emergent property of large networks that was first noted by van Vreeswijk and Sompolinsky [29, 30] and by Brunel [38] over 20 years ago. The comparisons to spiking network simulations (in which single–neuron transfer is present) in the manuscript and in many other studies of balanced network theory [22, 23, 29–37] provide verification of these analytical results.

> "*Although biological networks keep the EI balance, there is a fundamental question on how do we model it? It is not a priori necessary to keep balance with $1/\sqrt{K}$ scaling. This is the case for current based synapses and this scaling was first introduced for the purpose of theoretical simplification.*"

We agree with the referee that $1/\sqrt{K}$ scaling is not the only way to get EI balance. Indeed, as pointed out above any scaling larger than $1/K$ will produce balance as well. The widely used $1/\sqrt{K}$ scaling was introduced for theoretical simplification, but similar scaling laws have since been observed in cortical cultures [39]. Moreover, real cortical circuits have a fixed size, so the precise scaling of biological networks in a $K \to \infty$ limit might not even be a well–posed question. Instead, one should ask how accurate a given asymptotic theory is for fixed, biologically plausible parameters. Our spiking network simulations (along with many other studies using balanced network theory [22, 23, 29–37]) demonstrate that the asymptotic theory can be accurate for many biologically plausible parameter ranges.

> "*If we have more biological realistic neurons with conductance based synapses, the balance is kept dynamically.*"

We are not sure exactly what the referee is referring to here. Balance is kept dynamically in networks with current–based synapses in the sense that perturbations of the weights or inputs away from a previous balanced point are re-balanced dynamically (if external input, X, or synaptic weights, J, are changed in a balanced network then balance is transiently disturbed, but is re–established on a fast timescale). Moreover, our previous work demonstrates that the balanced network theory used here also works for models with conductance–based synapses and similar, biologically plausible parameter regimes (see, *e.g.*, Supplementary Materials in [22, 23]).

> "*Furthermore, there are networks which are not necessarily balanced, e.g. in development or diseases.*"

We agree that there are some neuronal networks in the brain for which excitatory–inhibitory balance is not established and therefore balanced network theory should not be applied. This includes networks during development, in diseased states, and also some sub–cortical neuronal networks. We did not mean to suggest that our approach should be applicable to these cases. We have added text to the manuscript to clarify this point (see below).

> "*I am yet to be convinced that this approach would be better then Ocker et al 2015. Especially for calculating rates and correlation self-consistently (Ocker et al. 2016). Is the assumption of infinite networks really weaker than the assumption on the structure of input noise and spike statistics?*"

and

> "*Therefore, I would put more utility in the approach which can treat the problem of self-consistency without the balance condition, but can handle a balanced network as a special case. On this point I would like authors to present a case where their framework works better than Burkit AN et al. 2007, Gilson et al. 2009 or Ocker et. al 2015 approach.*"

The referee correctly notes that previous work has developed alternative analytical approaches for STDP in recurrent network models, and the referee is concerned that the methods used in our work might not offer advantages over these other methods. All analytical approaches to STDP in recurrent networks make some assumptions, use some approximations, and have some constraints on where they can be accurately applied. In this sense, all approaches have some advantages and disadvantages. As we describe below, our assumptions are complementary to most of the assumptions made in previous work and each approach offers advantages and disadvantages.

To summarize some of the advantages of approaches in previous work over our approach: Previous studies give approximations to the full vector of firing rates and the full matrix of spike train covariances, while our mean–field approach only gives approximations to population–averaged rates and covariances. Also, previous approaches

do not rely on large N, balanced asymptotics so they can be applied to smaller networks whereas our approximations are accurate for networks of size $N \sim 10,000$ or larger. Moreover, unlike our approximations, previous approximations can be applied to networks without excitatory–inhibitory balance, such as networks in diseased states in sub–cortical brain regions that do not have inhibitory neurons for instance.

To summarize some of the advantages of our approach over approaches in previous work: Previous approaches require that neurons' transfer functions (f-I curves and/or correlation susceptibility functions) can be computed analytically. In some cases, this is achieved by using Poisson–spiking neurons with transfer functions that are prescribed [6–8,14–17]. These approaches are not applicable to networks of integrate–and–fire neuron models such as the ones that we consider. In other work, such as Ocker et al. [13], transfer functions are computed using Fokker-Planck techniques. The accuracy of Fokker–Planck techniques requires that input to neurons is dominated by Gaussian white noise. For this reason, Ocker et al. used Gaussian white noise as external input to neurons in their spiking networks and they needed to assume that synaptic input from the recurrent network was weak compared to this external Gaussian noise input. Moreover, efficient, direct Fokker–Planck approaches are not available for two–dimensional integrate–and–fire models such as those with adaptation currents, though one–dimensional approximations have been derived [20, 21]. In contrast, our approach allows for non-Gaussian, temporally correlated (i.e., non–white) external input. This is the case, for example, for the external input model we used that is generated by a presynaptic population of *correlated* Poisson processes. Such input is highly non-Gaussian and temporally correlated. Moreover, while we used one-dimensional EIF neuron models, our approach is applicable to any type of integrate–and–fire neuron model, including those with adaptation currents.

In addition, Ocker et al. assumes that the integral of the STDP curve be $\mathcal{O}(\epsilon)$, *i.e.* that the positive and negative part of the function nearly cancel, whereas we made no such assumption and successfully applied our approach to networks that strongly violate this assumption.

Finally, our approach is computationally more efficient because it does not require solving Fokker–Planck equations or any other nonlinear equations to compute the approximation. Our approximations are written solely in terms of linear equations of model parameters, so they are more efficient to compute and more amenable to mathematical analysis.

In summary, our approach and previous approaches each offer distinct advantages and disadvantages and are therefore applicable in different settings. The referee asks for a case where our framework works better than a previous approach:

> "*On this point I would like authors to present a case where their framework works better than Burkit AN et al. 2007, Gilson et al. 2009 or Ocker et. al 2015 approach.*"

To address this request, we note that **we have already considered multiple cases where the approach considered in Ocker et al. is not applicable.** For example,

in our networks in the correlated state, external input to the network is highly non–Gaussian and temporally correlated (non–white) in part because of synaptic filtering, but also because the external population of Poisson processes itself exhibited temporal correlations. The approach in Ocker et. al could not be applied to these examples because the neural transfer functions cannot be computed analytically for temporally correlated inputs (What would one use for the σ parameter from the Ocker et al. paper?).

Moreover, we considered learning rules that violate the assumption of approximate anti–symmetry of the STDP curve made in Ocker et al. Specifically, results shown in our manuscript use iSTDP and Kohonen's rules, which strongly violate the assumption that the integral of the STDP curve is approximately zero (see Supporting Figure 1 for an illustration of the STDP curves for those rules). Hence, the approach presented in Ocker et al. is not applicable to these examples that we consider.

One could, of course, attempt to apply the approach in Ocker et al. to the examples in our work, for example by treating the non–white noise in our examples with white noise having some intensity, σ, and by ignoring the errors introduced by non–anti–symmetric plasticity rules. Even if this approach were to yield accurate approximations to our spiking network simulations, we do not think that this would invalidate our work. We used an arguably simpler approximation, which does not require the solution of Fokker-Planck equations, and it yielded accurate approximations to our spiking network simulations. We used these methods to make novel observations about the interaction between EI balance, correlated variability, and synaptic plasticity. Hence, our approach was proven sufficient for answering the questions we sought to answer.

We agree with the reviewer that we were not sufficiently clear in making the distinctions between our approach and previous approaches. To correct this, we have now added the following text to the discussion:

> A number of mathematical theories have been proposed to describe the co-evolution of weights, rates, and the structure of correlations under STDP in recurrent neural networks [6–8, 13–17]. All of these approaches require knowledge of neurons' transfer functions (f-I curves and/or correlation susceptibility functions). Often neurons are assumed to be Poissonian, and their responses to inputs (f-I curves) are prescribed [6–8, 14–17]. Other work [13] uses Fokker–Planck techniques to compute transfer functions. These approaches rely on an assumption that the input to each neuron is relatively weak or dominated by Gaussian white noise [18, 19] and efficient, direct Fokker–Planck approaches are not available for two–dimensional integrate–and–fire models such as those with adaptation currents, though one–dimensional approximations have been derived [20, 21]. Some previous work [13] also assumes that STDP curves are approximately anti–symmetric, *i.e.*, there is a cancellation between the positive and negative parts of the curves (as in Supporting Figure 1**A**).
>
> Our approach uses balanced network theory to avoid the computation of neurons' transfer functions. As such, the resulting theory does not require an assumption of weak synaptic interactions or dominant Gaussian white noise

input, but can be applied to networks with highly non–Gaussian, temporally correlated input (such as the networks in the correlated state considered here). Moreover, the balanced network theory we used is accurate for a range of neuron models, including those with adaptation currents [22, 23], and for non–anti–symmetric STDP curves (as in Supporting Figure 1**B–F**). However, the balanced network theory used in our approach relies on large N asymptotics, which yielded accurate approximations for $N \sim 10,000$ in our case (Figure 1), but are inaccurate for smaller networks. Moreover, our approach would not be accurate for modeling neural circuits that do not exhibit excitatory–inhibitory balance, such as in some disease states, some developmental stages, and in some sub–cortical neuronal networks. Finally, we used a mean–field approach that only yields approximations to population–averaged firing rates, synaptic weights, and covariances, while other approaches [6–8, 13–17] give approximations to these quantities at the level of individual neurons. Despite these limitations, our analytical approach was sufficient for answering the questions related to the interaction between excitatory–inhibitory balance, correlated neuronal activity, and plasticity that we considered.

Referee C

The revision addresses my comments, but in a few cases there are remaining minor issues that should be taken care of. Please see the attached file. My comments are posted over the pdf (five comments overall, in pages 26, 29, 30, 30, 32).

In the actual supporting info file, these equation numbers do not appear correctly. (referring to eqn numbers in SFig 3 of first rebuttal).

Thank you for pointing that out. We have now fixed it.

Line 6: it will be appropriate to cite also Shaham and Burak (2017) and Darshan et al (2017) in the context of spatially correlated activity in the balanced state. Both references appear incorrectly in the reference list - errors in author names, manuscript name, missing page numbers etc. Please check carefully all the references.

Thank you for the comment. We have now carefully corrected our full reference list.

"In the discussion, it will be appropriate to cite Ravid Tannenbaum and Burak (2016) in the context of the relationship between covariances and STDP dynamics." Again, the citation in the reference list is incorrect. Author names and other details should be fixed. This manuscript should be cited also in the introduction, together with the citations of works by Gilson on Poisson networks, especially since the latter works did not take into account correlations generated intrinsically within the network. They only took into account correlations generated by Poisson inputs to the network.

Thank you for bringing this up. We have included this in the Introduction, and have explained in the Discussion how our work differs from previous theories.
New text in Introduction:

> In particular, Ravid Tannenbaum et al. showed that in networks of Poisson neurons synfire chains and self connected assemblies can emerge autonomously in recurrent networks [15]. Montangie et al. showed that a more realistic form of STDP based on spike triplets also leads to autonomous emergence of assemblies [16].

New text in Discussion:

> A number of mathematical theories have been proposed to describe the co-evolution of weights, rates, and the structure of correlations under STDP in recurrent neural networks [6–8, 13–17]. All of these approaches require knowledge of neurons' transfer functions (f-I curves and/or correlation susceptibility functions). Often neurons are assumed to be Poissonian, and their responses to inputs (f-I curves) are prescribed [6–8, 14–17]. Other work [13] uses Fokker–Planck techniques to compute transfer functions. These approaches rely on an assumption that the input to each neuron is relatively weak or dominated by Gaussian white noise [18, 19] and efficient, direct Fokker–Planck approaches are not available for two–dimensional integrate–and–fire models such as those with adaptation currents, though one–dimensional approximations have been derived [20, 21]. Some previous work [13] also assumes that STDP curves are approximately anti–symmetric, *i.e.*, there is a cancellation between the positive and negative parts of the curves (as in Supporting Figure 1**A**).
>
> Our approach uses balanced network theory to avoid the computation of neurons' transfer functions. As such, the resulting theory does not require an assumption of weak synaptic interactions or dominant Gaussian white noise input, but can be applied to networks with highly non–Gaussian, temporally correlated input (such as the networks in the correlated state considered here). Moreover, the balanced network theory we used is accurate for a range of neuron models, including those with adaptation currents [22, 23], and for non–anti–symmetric STDP curves (as in Supporting Figure 1**B–F**). However, the balanced network theory used in our approach relies on large N asymptotics, which yielded accurate approximations for $N \sim 10,000$ in our case (Figure 1), but are inaccurate for smaller networks. Moreover, our approach would not be accurate for modeling neural circuits that do not exhibit excitatory–inhibitory balance, such as in some disease states, some developmental stages, and in some sub–cortical neuronal networks. Finally, we used a mean–field approach that only yields approximations to population–averaged firing rates, synaptic weights, and covariances, while other approaches [6–8, 13–17] give approximations to these quantities at the level of individual neurons. Despite these

limitations, our analytical approach was sufficient for answering the questions related to the interaction between excitatory–inhibitory balance, correlated neuronal activity, and plasticity that we considered.

"This assumption is not essential as long as the balance condition remains valid. This is equivalent to having a non–singular mean–field connectivity matrix W, which is defined next." I do not understand: why is the non-singularity of this 2x2 matrix equivalent to validity of the balance condition? How is this matrix even defined if probabilities are not uniform? and how is this statement consistent with Landau et al (2016) where it is argued that with sufficient heterogeneity balance cannot be maintained?

We agree with the comment. It has been shown that balance is broken when in–degrees are heterogeneous [33, 34]. However, balance can be achieved when homeostatic plasticity is introduced [33], or when out-degrees are also heterogeneous and correlated with in–degrees [34]. The mean–field connectivity matrix would be defined in a similar way, but with more subpopulations [34]. We have modified our statement as follows.

> For simplicity we assume that both the probabilities, p_{ab}, and weights, $j_{ab} \sim \mathcal{O}(1)$ are constant across pairs of subpopulations.

We have also added this text to the Discussion.

> We have assumed that connection probabilities are homogeneous which translates to a narrow distribution of in–degrees. Cortical networks are heterogeneous, and a broad distributions of in–degrees can break the classical balanced state [33, 34]. Balance can be restored with the introduction of homeostatic plasticity [33], or by including heterogeneous out–degrees correlated with in–degrees [34]. As we mentioned previously, in such cases our theory would need to be extended to account for possible emerging correlations between weights and in-degrees or out–degrees. We relegate such extensions to future work.

"Lines 140-148: I didn't understand why root finding is involved in the numerical solution of the differential equations. Doesn't equation 6 simply provide the required update of J, based on the values of r anc C that are obtained from equations 1-2?" The meaning of "finding the roots" and "root of Eq. 12" at the top of page 7 is still unclear. Equation 12 is a differential equation for J, defining its evolution in time. What do you mean by the roots of this equation? what exactly is being solved, for which variable?

Upon another reading, we agree that we could have been clearer here. We have now changed this text as follows:

Eqs. (3,5,12) thus self–consistently describe the macroscopic dynamics of the balanced network. There are two approaches to analyzing this coupled system of ordinary differential equations: (1) solve directly for the steady–states of the system; or (2) apply numerical

integration to obtain the evolution of the system in time. To obtain the equilibria, we first find the firing rates and covariances (both in terms of plastic weight J_{ab}) obtained using the mean–field description of the balanced network, Eqs. (3,5). We next substitute the results into Eq. (12), set $\frac{dJ_{ab}}{dt} = 0$, and find the roots. We denote the solution by J^*_{ab}. We then use the mean synaptic weight (root of Eq. (12), J^*_{ab}) to obtain the corresponding rates and covariances using Eqs. (3,5). Alternatively, we can solve the system iteratively over time and obtain the time evolution of rates, covariances, and weights. Starting at an arbitrary value of $J_{ab}(t)$, we proceed in the same way as in the first approach, but instead of setting $\frac{dJ_{ab}}{dt} = 0$, we use $J_{ab}(t)$ to compute the value of the derivative at time t, $\frac{dJ_{ab}}{dt}|_t$, and use it to update the mean weight at the next time step, $J_{ab}(t + \Delta t)$. We then use $J_{ab}(t + \Delta t)$ to update rates and covariances. We repeat this process until convergence (See S1 Appendix: "Transient dynamics of synaptic weights" for sample trajectories under different rules, and for our criterion to determine stationarity of synaptic weights).

References

[1] Izhikevich E, Gally J, Edelman G. Spike-timing dynamics of neuronal groups. Cerebral Cortex. 2004;14(8):933–44.

[2] Morrison A, Aertsen A, Diesmann M. Spike-timing-dependent plasticity in balanced random networks. Neural Computation. 2007;19(6):1437–1467.

[3] Babadi B, Abbott L. Pairwise Analysis Can Account for Network Structures Arising from Spike-Timing Dependent Plasticity. PLoS Comput Biol. 2013;9(2):e1002906.

[4] Litwin-Kumar A, Doiron B. Formation and maintenance of neuronal assemblies through synaptic plasticity. Nature Communications. 2014;5(5319).

[5] Gütig R, Aharonov R, Rotter S, Sompolinsky H. Learning input correlations through nonlinear temporally asymmetric Hebbian plasticity. J Neurosci. 2003;23(9):3697–714.

[6] Burkitt A, Gilson M, van Hemmen J. Spike-timing-dependent plasticity for neurons with recurrent connections. Biological Cybernetics. 2007;96(5):533–546.

[7] Gilson M, Burkitt A, Grayden D, Thomas D, van Hemmen J. Emergence of network structure due to spike-timing-dependent plasticity in recurrent neuronal networks. II. Input selectivity-symmetry breaking. Biol Cybernetics. 2009;101(2):103–114.

[8] Gilson M, Burkitt A, Grayden D, Thomas D, van Hemmen J. Emergence of network structure due to spike-timing-dependent plasticity in recurrent neuronal networks. III. Partially connected neurons driven by spontaneous activity. Biol Cybernetics. 2009;101(5):411–26.

[9] Gilson M, Burkitt A, Grayden D, Thomas D, van Hemmen J. Emergence of network structure due to spike-timing-dependent plasticity in recurrent neuronal networks. V: self-organization schemes and weight dependence. Biol Cybernetics. 2010;103(5):365–386.

[10] Vogels TP, Sprekeler H, Zenke F, Clopath C, Gerstner W. Inhibitory Plasticity Balances Excitation and Inhibition in Sensory Pathways and Memory Networks. Science. 2011;334(6062):1569–1573.

[11] Sprekeler H. Functional Consequences of inhibitory plasticity: homeostasis, the excitation-inhibition balance and beyond. Current Opinion in Neurobiology. 2017;49:198–203.

[12] Trousdale J, Hu Y, Shea-Brown E, Josic K. A generative spike train model with time-structured higher order correlations. Frontiers in Computational Neuroscience. 2013;7(84):1–21.

[13] Ocker G, Litwin-Kumar A, Doiron B. Self-organization of microcircuits in networks of spiking neurons with plastic synapses. PLOS Computational Biology. 2015;11(8):e1004458.

[14] Gilson M, Burkitt A, Grayden D, Thomas D, van Hemmen J. Emergence of network structure due to spike-timing-dependent plasticity in recurrent neuronal networks. I. Input selectivity-strengthening correlated input pathways. Biol Cybernetics. 2009;101(2):81–102.

[15] Ravid Tannenbaum N, Burak Y. Shaping Neural Circuits by High Order Synaptic Interactions. PLOS Comp Biol. 2016;12(8):e1005056.

[16] Montangie L, Miehl C, Gjorgjieva J. Autonomous emergence of connectivity assemblies via spike triplet interactions. PLoS Comp Biol. 2020;16(5):e1007835.

[17] Gilson M, Burkitt A, Grayden D, Thomas D, van Hemmen J. Emergence of network structure due to spike-timing-dependent plasticity in recurrent neuronal networks IV. Biol Cybernetics. 2009;101(5):427–444.

[18] Lindner B, Longtin A, Bulsara A. Analytic expressions for rate and CV of a type I neuron driven by white gaussian noise. Neural computation. 2003;15(8):1761–1788.

[19] Richardson MJ. Firing-rate response of linear and nonlinear integrate-and-fire neurons to modulated current-based and conductance-based synaptic drive. Physical Review E. 2007;76(2):021919.

[20] Hertäg L, Durstewitz D, Brunel N. Analytical approximations of the firing rate of an adaptive exponential integrate-and-fire neuron in the presence of synaptic noise. Frontiers in Computational Neuroscience. 2014;8.

[21] Rosenbaum R. A diffusion approximation and numerical methods for adaptive neuron models with stochastic inputs. Frontiers in Computational Neuroscience. 2016;10.

[22] Ebsch C, Rosenbaum R. Imbalanced amplification: A mechanism of amplification and suppression from local imbalance of excitation and inhibition in cortical circuits. PLoS Computational Biology. 2018;14(3):e1006048.

[23] Baker C, Zhu V, Rosenbaum R. Nonlinear stimulus representations in neural circuits with approximate excitatory–inhibitory balance. PLoS Comput Biol. 2020;16(9):e1008192.

[24] Bienenstock E, Cooper L, Munro P. Theory for the development of neuron selectivity: orientation specificity and binocular interaction in visual cortex. J Neurosci. 1982;2(1):32–48.

[25] Pfister J, Gerstner W. Triplets of Spikes in a Model of Spike-Timing Dependent Plasticity. J Neurosci. 2006;26(38):9673–82.

[26] Gjorgjieva J, Clopath C, Audet J, Pfister J. A triplet spike-timing-dependent plasticity model generalizes the Bienenstock-Cooper-Munro rule to higher-order spatiotemporal correlations. PNAS. 2011;108(48):19383–8.

[27] Shouval H, Bear M, Cooper L. A unified model of NMDA receptor-dependent bidirectional synaptic plasticity. PNAS. 2002;99(16):10832–6.

[28] Graupner M, Brunel N. Calcium-based plasticity model explains sensitivity of synaptic changes to spike pattern, rate, and dendritic location. PNAS. 2012;109(10):3991–6.

[29] van Vreeswijk C, Sompolinsky H. Chaos in Neuronal Networks with Balanced Excitatory and Inhibitory Activity. Science. 1996;274(5293):1724–1726.

[30] van Vreeswijk C, Sompolinsky H. Chaotic Balanced State in a Model of Cortical Circuits. Neural Computation. 1998;10(6):1321–1371.

[31] Renart A, De La Rocha J, Bartho P, Hollender L, Praga N, Reyes A, et al. The Asynchronous State in Cortical Circuits. Science. 2010;327(2):587–590.

[32] Rosenbaum R, Doiron B. Balanced Networks of Spiking Neurons with Spatially Dependent Recurrent Connections. Phys Rev X. 2014;4(2):021039.

[33] Landau I, Egger R, Dercksen V, Oberlaender M, Sompolinsky H. The Impact of Structural Heterogeneity on Excitation-Inhibition Balance in Cortical Networks. Neuron. 2016;92(5):1106–21.

[34] Pyle R, Rosenbaum R. Highly connected neurons spike less frequently in balanced networks. Phys Rev E. 2016;93:040302.

[35] Rosenbaum R, Smith MA, Kohn A, Rubin JE, Doiron B. The spatial structure of correlated neuronal variability. Nature Neuroscience. 2017;20(1):107–114.

[36] Darshan R, van Vreeswijk C, Hansel D. Strength of Correlations in Strongly Recurrent Neuronal Networks. Physical Review X. 2018;8:031072.

[37] Baker C, Ebsch C, Lampl I, Rosenbaum R. Correlated states in balanced neuronal networks. Phys Rev E. 2019;99:052414.

[38] Brunel N. Dynamics of sparsely connected networks of excitatory and inhibitory spiking neurons. Journal of computational neuroscience. 2000;8(3):183–208.

[39] Barral J, Reyes AD. Synaptic scaling rule preserves excitatory-inhibitory balance and salient neuronal network dynamics. Nat Neurosci. 2016;19(12):1690–96.
